# The Essays, Cases, Intelligence, &c.: A—B

**Published:** 1820

**Authors:** 


### GENERAL INDEX

TO THE

### LONDON MEDICAL AND PHYSICAL JOURNAL.

### *The ESSAYS, CASES, INTELLIGENCE, &c.*

ABDOMEN, case of a tumour on the, i. 421; account of an uncommon tumour of the, ii. 362; directions for opening the, v. 580; on the different organs of the, vi. 78; extraordinary abscess in the, 294; instance of one enlarged in an infant, vii. 223; case of extraordinary swelling of the, viii. 230; case of a wound penetrating the thorax and, x. 250; case of dropsical enlargement of the, xii. 249; case of fungous tumour of the, xiv. 79; a fœtus found in that of an old woman, xv. 276, observations on that case, 277; cases of preternatural pulsation in the, xvii. 188; on tapping the, 210; extraordinary appearances in the dissections of, xviii. 488; xix. 159; instance of recovery from a wound of the, xxi. 414; a fœtus found in that of a boy, xxii. 166; xxiv. 7; case of tumour in the cavity of the, xxiv. 105; on wounds in the, xxviii. 8; case of tumour of the, from lumbar abscess, xxxi. 164; inflammation of it brought on by excess in eating and drinking, xxxi. 220; case of a fœtus found in that of a boy, xxxii. 81; case of extravasation of the bile into the, 152; history of an enormous tumour of the, xxxiii. 426; fœtus found in that of a child, xxxiv. 80; particulars of a fœtus found in that of a young man, xxxiv. 317—323.

ABDOMINAL INFLAMMATION, effects of cold water in four cases of, xxx. 512; another case proceeding from debauchery, xxxi. 371.

——————— MUSCLES, case of abscess in the, xvi. 77.

——————— PREGNANCY, account of a case of, xxx. 318.

——————— VISCERA, distorted by stays, ii. 47; application of frictions to swelled, iii. 167; case of disorganised, 168; on diseases of the, v. 580; on variations in the, xvi. 454; Dr. Pemberton's treatise on the diseases of the, xv. 481, 573; xviii. 19, 20; fatal case of inflammation of the, xxxi. 372; xxxii. 89; case of paralysis of the, xxxii. 448.

ABEL, Clarke, on the analogy between gastritis and hydrophobia, xxix. 454; Dr. Kinglake's acknowledgment to, xxx. 96; his translation of Portal on apoplexy, 193, 370; Mr. Borrett's letter to, xxxi. 1; his observations on hydrophobia, 6; reply to Dr. Adams by, 281.

ABERCROMBIE, Sir Ralph, inquiry concerning the death of, xii. 173; the ball found in the cervix femoris, 243; observations on his wound, 421; encouraged vaccination, v. 354.

ABERDEEN, on the air round, vii. 321; account of the influenza at, x. 232; on the diseases of the Highlanders in the county of, xiii. 28; vaccination in the town of, 52; epidemic measles at, xxi. 359, 366; state of the university of, xxx. 267, 273, 287.

ABERDOUR, Dr. on strictures of the urethra, iv. 387; his cases of vaccination, xi. 131.

ABERGAVENNY, influenza at, x. 228.

ABERNETHY, John, on the cow-pox, ii. 400; on patients who have died of consumption, v. 309; his case of aneurism, vii. 97; particulars of the dissection, 102; on perspirable air, 318; his practice of tying an artery in aneurism, viii. 1; his method of curing the psoas abscess, 206; description of the gorget for lithotomy, invented by him, ix. 393, 566; on the classification of tumours, xii. 176, 266; on tying the artery, xiv. 112; on the digestive organs, xvi. 272; case of omental hernia, 273; on disorders of the head, 275; on spasmodic strictures of the œsophagus, 277; on diseases of the skin, 278; his case of femoral aneurism, xvii. 191, 193; on diseases resembling syphilis, 231, 536; on the language of, 568; on the disorders accompanying local diseases, xviii. 21; cases of malformation of the heart, xxii. 71, 72; on schirrhous tumour, 81; on the origin and treatment of local diseases, xxiii. 242, 254; on tying the external iliac artery, xxiv. 17; his observations on cancer, 307; remarks on his mode of treating spina bifida, 341; on his improvements in surgery, xxvi. 33; on bloodletting in disorders of the head, xxviii. 375; on the subject of tumours, xxix. 297; his lecture at the College of Surgeons, xxxi. 170; on the speculative opinions of, 409; his surgical practice correspondent with that of Celsus, xxxii. 297; his lectures on Hunter's theory of life, 419; observations on his lectures, 456; anecdote of, xxxiii. 58; his lecture for 1815, xxxiv. 65.

ABERYSTWYTH, account of the influenza at, x. 294.

ABICH, M. his analysis of the honey-stone, i. 398.

ABILDGAARD, M. on the composition of human blood, i. 300; on respiration, iv. 274; on injecting medicines into the veins of horses, v. 399; on the quantity of carbon in the blood, vi. 192.

ABINGDON, mortality among the poor at, vii. 154.

ABLUTION, observations on acetous, vii. 337; viii. 51, *note*; advantages of cold in ardent fever, xi. 21; in scarlatina, 27; benefit of tepid, in hydrophobia, xxvii. 251.

ABORTION, remarkable trial on a charge of procuring, xxiv. 38; arising from diseased structure of the placenta, xxvii. 168.

ABRUS PRECATORIUS, on the change of colour in the, xxiii. 350.

ABSCESS after childbirth, case of, ii. 17; in the groin, v. 219; an extraordinary one on the abdomen, vi. 293; case of lumbar, viii. 44; method of curing the psoas, 206; of the liver, on a case of, x. 1; in the breast, occasioned by a fractured rib, 414; of the liver, with hydatids, 489; the several methods of opening an, xi. 327; case of, in the chest, xiii. 21; in vaccination, case of, xiv. 566; in the abdominal muscles, xvi. 77; of the liver terminating favourably through the lungs, xvii. 500; case of local inflammation of the lungs with, xxi. 265, 7; of the brain, case of, xxiii. 376; appearances upon dissection, 377; of the liver successfully treated, xxvii. 95, 101; another case, with remarks, 278, 281; observations on a case of, xxviii. 95, 100; of the liver, Mr. Semple's case of, xxxi. 8; on abdominal tumor originating in lumbar, 164; xxxii. 370; case of one produced by injury of the head, xxxii. 119, 370; of the brain, case of, xxxiii. 60.

ABSCESSES, various cases of, xix. 184; succeeding to wounds of the head, sympathetic, xxii. 499; on the treatment of, xxiv. 84.

ABSORBENT SYSTEM, on air received into the, iv. 86.

ABSORBENTS, on their use in disorders of the stomach, viii. 289; on the supposed retrograde action of the, xxvi. 467.

ABSORPTION, on the affections produced in the human frame by, i. 403; observations and experiments on, iv. 468; on aqueous, from the skin, x. 570; experiments on cutaneous, xv. 274, 275; memoir on the organs of, xxv. 44; experimental inquiries into, 46; probability of that of the veins, 47; on the removal of cataract by, xxix. 422; experiments on cutaneous, xxxi. 80; of the water in hydrocele, case of, xxxiv. 515.

ABSTINENCE, recommended in catarrhous complaints, i. 20; beneficial effects of it in the approach of diseases, 43; account of a young woman said to have lived without food two years, iv. 86; but proved to be an impostor, 87, 187; the danger attending rigorous, 147; another history of pretended, vii. 190; remarkable instance of voluntary, xv. 510, 516; from bodily exercise, xxi. 227; case of Ann Moore, of Tutbury, xx. 402; remarks on that case, 527, 531; farther account of her, xxi. 61; xxii. 337; xxiv. 309; xxix. 109; detection of the imposture, 403; Mr. Richmond's statement of her case, 469; memorial on her case, xxx. 187; remarks on the case, 189; observations on the utility of, xxii. 110; memoirs of cases of, xxv. 85; recommended in cancerous complaints, xxvii. 72; historical instances of extraordinary, xxix. 113; case of Anna Maria Kinker, 409; remarkable history of Martha Taylor, the Derbyshire damsel, xxx. 103; cases of, in various animals, 108; singular history of Mary Thomas, xxi. 96; xxx. 157; extraordinary circumstance in the reign of Edward III. xxxi. 50.

ACACIA, substitute for the Egyptian, xvi. 167; new species of three thorned, xxvi. 477.

ACADEMICUS, on a case of fatal diarrhœa, xi. 461; reply to, 518; observations on his case, xii. 51; remarks of, 73; on dysodontophysis, xiii. 515, doubts suggested on the case, xiv. 1, 5.

ACARUS, description of various kinds of, xxi. 8.

ACCOUCHEURS, ancient term for, xxvii. 117; origin of regular, xxviii. 102; observations on the necessity of, xxxiv. 450.

ACCUM, Mr. his chemico-physical aphorisms, i. 90; system of mineralogy xviii. 388; his analysis of Cheltenham waters, xxii. 15.

ACETIC ACID, difference between the acetous and the, iii. 172; preparation of the, vii. 476; its effect on chyle, xxviii. 389; its effect on the colouring matter of the blood, 397; the most efficacious remedy for the poison of alkalies, xxxiii. 476; objections to that article in the London pharmacopœia, xxxiv. 490.

ACETITE OF LEAD, its uses in cases of hæmoptysis, vii. 507; benefit of it in ulcers, ix. 173; efficacious in uterine hemorrhage, xix. 6; in profuse hæmorrhage, xxii. 350.

ACETITE OF ZINC, its benefit in gonorrhœa, ix. 53.

ACETOUS ABLUTION in typhus, efficacy of, vii. 337; in synochus, cases of, viii. 51, *note*.

ACETOUS ACID, difference between the acetic and, iii. 172; found in urine, vii. 476; its agency in pus, xxv. 333; its effects in counteracting vegetable poisons, xxviii. 185.

ACETOUS ÆTHER, method of preparing the, iii. 267; an excellent anodyne and dissolvent, iv. 82; its effects in rheumatic complaints, 369; vi. 273.

ACHARD, M. on the effects of electricity on animal bodies, i. 200; on the extraction of sugar from beet-root, ii. 188; observations on his process, iii. 8; explanation of the same, iv. 276; his experiments on mercury, xiv. 274.

ACHARIUS, Eric, on the lichens of Sweden, iii. 578; his case of pulmonary polypus, viii. 201; on tar-water in venereal complaints, ix. 582.

ACHILLINI, Alexander, account of his works, ii. 61; his discoveries in anatomy, 160; on the existence of hymen, iii. 158; pathetic nerves supposed to be known to, 359.

ACHILLIS, case of rupture of the tendo, xiii. 494.

ACHROMATIC glasses, beneficial to the sight, xiv. 12.

ACHOR, definition of pustulous, xxiii. 130; xxx. 415.

ACID, a new one found in animal substances, i. 85, 505; composition of that of tartar, 98; disoxygenation of the sulphuric, 300; effects of drinking the mineral, 423; combination of the muriatic and sulphuric, ii. 81; ether extracted from the sulphuric, ibid; on the camphoric, iii. 89; a new metallic one in red lead, 173; the dysentery cured by the nitric, 413; on that of the honeystone, 583; carbonic contained in atmospheric air, iv. 85; on the virtues of the citric, iv. 154; of tartar, method of obtaining, 181; in mildew, 372; decomposition of red-lead in, 374; on obtaining phosphoric, 447; efficacy of sulphuric, 482; on the concrete citric, v. 75; tartarous, how separated from vegetables, 365; beneficial in fever, vi. 87; on the internal use of the phosphoric, 92; analysis of the mellilithic, 189; change of tartarous into saccharic, 475; a new one found in bituminous wood, vii. 381; found in urine, 476; preparation of the acetic, ibid.; on the mutallic combinations of muriatic, 546; nature of the sebacic, ix. 78; virtue of the phosphoric, 388; characters of the uric, 491; on preparing muriatic ether with the simple, x. 94; experiments to ascertain the existence of cobaltic, ibid; method of obtaining prussic, 479; poisonous qualities of prussic, xi. 93; crystallization of phosphoric, 177; method of preparing pure gallic, 275; on the preparation of arsenic, 382; on treating paper with nitric, 288; changes of the septic, 404; combination of antimony and muriatic, 497; new one in the white mulberry-tree, xii. 92; Mr. Fiedler's method of preparing gallic, 382; use of the nitrous, in diabetes, xiii. 153; on the sulphuric, 110; fracture cured by nitric, xiv. 28; on the composition of muriatic, 272; inefficacy of attempts to decompose the muriatic, xv. 294; benefit of fumigations of, xvi. 190; lithonthriptic powers of the muriatic, 570; effect of nitric in icterical affections, xvii. 17; in elephantiasis, 189; on the use of nitric in hepatitis, xviii. 301; constituent qualities of the oxalic, xix. 287; formed in indigestion, xx. 193; on the internal use of the arsenical, 494, 498; found in ginger, xxii. 349; beneficial effects of the muriatic in fevers, xxiii. 51, 54; decomposition of the fluoric, 292, 299; effects of magnesia on uric, xxiv. 203; use of the nitric in ulcerated tongue, 356; on the lithonthriptic power of the muriatic, xxv. 446; improvement in preparing and applying wood, xxviii. 427; experiments on malic, xxxiv. 81; a new vegetable, 164; whether animals can live without, 171; on the preparation of carbonic, xxvi. 95; experiments on the camphoric, xxvii. 127; anti-contagious properties of muriatic, 408; on some unknown combinations of the chromic, xxxiii. 33. See *Nitrous* Acid.

ACIDS, common in manures, i. 46; on the anti-venereal powers of, 100, 102, 103; iii. 72, 565; iv. 183; v. 71, 73; vi. 105, 165; their efficacy in diabetes mellitus, i. 102; nature of the simple vegetable, 159; basis of the same, 172, 272; their virtue in sick head-ache, 286; utility of them in typhus, 394; cases of the poisonous effects of mineral, 500; inflammation of ether by, ii. 77; on the reduction of vegetable, 81; difference between acetous and acetic, iii. 172; on the mixture of the three, iv. 24; on extracting vegetable, v. 365; mineral ones beneficial in fever, 180; how to be used in fumigations, viii. 537; on the arseniated, xi. 428; beneficial effects of the mineral in fever, xiii. 112; on preserving copper vessels from the, 288; their use in purifying the atmosphere, xiv. 205; on the composition of, 272; beneficial in scarlatina, 373; injurious to health in manufactories, xvi. 103; their effects on calculous concretions, 153, 221, 305; analysis of animal, xix. 124, 133; inefficacious as solvents of urinary calculi, xxii. 52; on the combination of animal and vegetable substances with, xxiv. 321, 328; their action on various kinds of calculi, xxv. 256; on the decomposition of the, xxvi. 168; their action on vegetable wax, xxvii. 74; on the phosphorous and sulphuric, xxviii. 157; their effects on the colouring matter of blood, 395; their action on the liquid gum of Botany Bay, xxix. 479; their influence on urine, xxx. 328; on the antimonial, 344; discovery of two new, xxxii. 348; their action on hyperoxymuriate of potash, xxxiv. 79.

ACKERMANN, Dr. on wind in the intestinal tube, iv. 556; his Galvanic experiments on a decapitated person, x. 576.

ACKWORTH, on the treatment of scarlatina anginosa in the Friend's boarding-school at, xiv. 374; xxxii. 481.

ACONITE, its use in chronic rheumatism, i. 385; beneficial in rheumatic cardialgy, iv. 184; its efficacy in gout, xi. 218, 225; experiments with the leaves of, xxvi. 133, 139.

ACORNS, extraordinary produce of, xxvi. 474.

ACOROIDES RESINEFERA, efficacious in cholera morbus, vi. 429.

ACORUS CALAMUS, or myrtle flag, description of, xiv. 67; recommended in vertigo and intermittents, 68.

ACOSTA, Joseph, a remarkable anecdote of, viii. 390.

ACOUSTIC NERVE, known to the old anatomists, iii. 361.

ACOUSTIC TUBES, description of improved, iv. 378; vi. 75.

ACRID PRINCIPLE in plants, on the, i. 158, 268.

ACT, observations on the medicine, viii. 127; xxviii. 233; on the amendments in the, x. 165, 258, 347; for regulating the practice of physic, abstract of the, xxiii. 188; objects of it, xxiv. 85, 303; observations on the, 494; xxv. 109, 216, 402, 492; preventing small-pox inoculation, announced, xxx. 85; amendment of the, 171; for regulating mad-houses, remarks on the, xxxii. 1; farther observations on the same, 4, 8; passing of the apothecaries', xxxiv. 169; abstract of the apothecaries', 344; observations on it, 346.

ACTION, fatal cases of translated diseased, xxiv. 178, 183; on the duration of muscular, 485; observations on associated, xx. 111; of the vital power, xxvi. 1; of vessels in inflammation, 353.

ACTIONS of the human frame, on the, ii. 11; on sympathetic or associated, viii. 313; of the muscles, observations on the, xix. 289; on the doctrine of two diseased, existing at the same time, xiv. 262; xxi. 109, 120; interrupted by voluntary power, xxvi. 98; on stimulated, 100.

ACUPUNCTURATION, account of the operation of, vii. 236.

ADAM, John, his case illustrating the effects of a vegetable poison, xxxii. 364.

ADAMS, Dr. on pulmonary consumption, v. 307; on the climate of Madeira, 309; on morbid poisons, 498; ix. 309; xvii. 567; his cautions to consumptive patients, vi. 415; on a case of aneurism, 535; on the cancerous breast, vii. 82, 163; his defence of Hunter's physiological ideas and language, viii. 412, 413; account of the introduction of the cow-pox in Madeira, ix. 310; his description of Madeira, ibid; description of the Lestes, 315; his observations on gout, xii. 141; on cutaneous eruptions, 193; description of a peculiar itch, 194; proposal on the cow-pox, 195; Dr. Walker in reply to, 332; observations on his cases of cow-pox, 358; on the formation of hydatids, 552; in answer to Dr. Walker, xiii. 1; reply to Mr. Ring, 2; remarks on Dr. Macdonald, 3; on small-pox, 6; Mr. Ring's rejoinder, 168; on the slough in small-pox, 400; chosen physician to the Small-pox Hospital, 575; on the cancerous breast, xiv. 82; his tract on the Cow-pox analysed, 96, 377; his reply to the reviewers on vaccination, 193; account of cases at the small-pox hospital, 194, 199; particulars of a case of small-pox, 353; on inoculation at the small-pox hospital, 355, 361; his answers to objections against cow-pox, 377; his report on vaccination, xv. 195; account of quackery in France, 334; his account of the Yaws, 382; on the leprosy of the Jews, 385; Mr. Young's letter to him on the hydatid existence of cancer, 393, 408; Mr. Carmichael's remarks on his opinions, 479; his observations at Berkeley on cow-pox matter, 582; his letter to Mr. Young on hydatids, xvi. 97, 100; on medical disputes, 247; on herpes in vaccination, 248; on the cold treatment of small pox, 249; on the slough, ibid; Mr. Young in reply to, 403, 409; his oration before the Medical Society of London, xvii. 390; on medical reasoning, 567; on the analogy between variolous and vaccine disease, 569; on the cure of morbid poison, 572; on the venereal disease, 573; on empiricism, xviii. 3; on cases of small-pox after vaccination, 28; on sivvens, 82; on diseases confounded with syphilis, ibid.; on leprosy, 84; on morbid poisons attended with fever, ibid.; on infection, 66; on the prevention of fever, 90; his popular view of vaccine inoculation, xix. 377; on the history of the small-pox, 378; his popular view of vaccine inoculation recommended, xx. 25; his directions for vaccination, 26, *note*; reports on vaccination, published by, 282; on blood-letting in acute organic affections, 536; account of opium produced in Madeira, 567; his report of the small-pox hospital, xxi. 172; account of the progress of a carcinoma, xxii. 80, *note*; distinction conferred on him by the College of Physicians, 87; on the history of leprosy, 407, 414; on small-pox inoculation, xxiii. 27, 32; inquiry into the contagious property of hooping-cough, 177, 183; on epidemics, xxiv. 22; on the digestion of the stomach after death, 399, 413; his commentary on Hunter's Treatise on the Venereal Disease, xxv. 78; on the origin of the gonorrhœa, 80; his description of the Arabian leprosy, 84; correction of an error of, xxvi. 381; on the nature of croup, xxvii. 354; on the treatment of that complaint, 363; case of, 365; plan of his lectures, 523; on his opinion respecting the origin of the venereal disease, xxviii. 494; on inflammation in hydrophobia, xxx. 201; reply of Mr. Borrett to, xxxi. 1; Mr. Abel's animadversions on, 6; on the appearance of the stomach after death, 177; reply of Mr. Abel to, 281; on hereditary diseases, xxxii. 140, 312; on the vitality of the blood, 30, 36; on insects as the cause of the itch, 496; etymology of the word scrofula, 499; on goitres and cretinism, xxxiii. 281, 433; his apology for the supposed obscurity of John Hunter, xxxiv. 29; on periodical blood-letting, 110; character of his work on morbid poisons, 228; on the sloughing phagedæna, 231.

ADAMS, George, on medical electricity, ix. 286.

———, Michael, his discovery of a mammoth in Siberia, xix. 287.

———, Robert, on the violent operation of the eau de medicinale in gout, xxiv. 364.

———, William, of Rochester, his case of pins swallowed, v. 245.

———, Sir William, on the origin of his instrument for the cure of cataract, xxix. 89, 100; on the Egyptian ophthalmia, 302; his observations on the diseases of the eye, 312; on different modes of operating for the cataract, 325; his operation for the cure of ectropium, 432; his explanation on the cure of ophthalmia, xxx. 46; reply to, xxxi. 17; his success in operating for the cataract at Greenwich-Hospital, 82; vindication of his treatment of ophthalmia, 189; observations on his operation, 295; official account of his operations in Greenwich-Hospital, 326; his letter to the directors, 332; remarks on his practice, 355; explanation respecting, 382; French translation of his work, xxxii. 168; strictures on the report of his operations for cataract, 487; his objections to Mr. Saunders, 490; exposure of his conduct, 328; his skill in the art of puffing, 331, 332.

ADDENBROOKE'S HOSPITAL, Cambridge, history of the foundation of, xiv. 344.

ADDER'S TONGUE, ophioglossum vulgatum, botanical description of, xix. 541; used as a vulnerary, ibid.

ADDINGTON, John, his observations on the cow-pox, v. 292; his evidence on Dr. Jenner's petition, vii. 494; on his mode of treating gonorrhœa, x. 283; an error in his report to the Jennerian Society, xiv. 433; on apparent death, xxiii. 333.

ADHESION, on the doctrine of, ix. 85; case of thoracic, 359; on the modern practice of, xix. 563.

ADHESIONS in the peritoneum and intestines, how produced, v. 581.

ADHESIVE PLASTERS, their benefit in ulcers, i. 183; ii. 92; ix. 113.

ADHESIVE PASTE in tinea capitis, benefit of, xxi. 178.
ADHESIVE STRAPS, improper for scrofulous patients, xx. 110; correction respecting, 322.
ADIPOCIRE, an oily concrete found in animal compounds, ix. 497; calculi in the gall-bladder, composed of, xxiv. 519; instances of human bodies speedily converted into, xxix. 507; xxxi. 45; account of an encysted tumour of, xxxiv. 438.
ADIPOSE tumour in a schirrhous ovary, xxiii. 237.
——— membrane of the kidneys, inflammation and mortification of the, xxxi. 156.
———, sarcoma on the, xiii. 388.
ADVERTISEMENTS, observations on quack, vii. 396; singular ones, xii. 138;
ADYE'S, W. F. case of fractured skull, xi. 36.
ÆGINETA, his knowledge of the ligature, xiii. 392; recommends a cool and diuretic regimen in gout, xxvi. 479; his age and character, xxxii. 303; his treatment of aneurism, 304.
ÆNEAS, on the case of, xxxii. 113.
ÆROLITES, see *Meteoric Stones.*
ÆRIFORM FLUIDS, hypothesis accounting for the elasticity of, v. 550; observations on the, xiii. 109.
ÆSCULAPIUS, his age and history, xxxii. 112.
ÆTHER. See *Ether.*
ÆTHIOPIA, pestilential winds in, xix. 116.
ÆTHIOPS ANTIMONIALIS, preparation of, ii. 186; short and easy method of preparing, vi. 15.
ÆTHIOPS MINERAL, method of preparing the, iii. 269.
ÆTHIOPS VEGETABLE, an excellent dentrifice obtained from that of fucus, xx. 350.
ÆTHUSA CYNAPIUM, or lesser hemlock, botanical description of the, xii. 368.
ÆTIUS, his practice of bleeding in gout, xxvi. 479; his use of leeches in liver complaints, 409; his age and character, xxxii. 302.
AFFECTATION, animadversions on professional, xviii. 253.
AFFECTIONS, on inflammatory, xx. 78; case of singular nervous, 424; another remarkable instance of, xxvi. 458.
AFFINITIES of medical substances, caution concerning, xxxi. 341.
AFFUSION, cold, advantages of it in fever, iii. 51; iv. 25, 402; v. 12, 75; vi. 105, 382; viii. 158, 356; ix. 9, 206, 210, 282; xi. 21, 27; xiv. 263, 226; xvi. 237; xix. 175, 321; xxiii. 416; xxvi. 444; xxxiii. 358; historical review of the application of it in medicine, iv. 327; its benefit in gout, ix. 116, 124, 205, 362, 442; xi. 521; in cholic, xiii. 18; in trismus, xiv. 267; in tetanus, xv. 496, 500; benefit of it in strangulated hernia, xvii. 55; alledged not to be a new remedy, xx. 52, 53, 81, 89; observations on the practice of it, 164, 171; its efficacy in epilepsy, xxiii. 20; in scarlatina, 413; unsuccessful in military practice, 159; on the stimulating power of it, xxvi. 398; cautions to be used in the use of it, xxxi. 495; beneficial in chorea, xxxiii. 273.
AFRICA, account of Broussonet's travels in, i. 198; varieties among the natives of, 407; intermittents prevalent in, x. 85; wretchedness of the towns in, xiii. 118; introduction of vaccine inoculation in, xviii. 147; unhealthy settlements on the coast of, xix. 114; additions to the natural history of, xxvii. 22; pestilential winds in, xxviii. 329.
AGARIC, deleterious effects of a species of, iii. 41; description of the, 43; case of poison by, xii. 512; botanical account of, ibid.; case of recovery after eating, xv. 247; remarks on it, 248; account of two species of, xx. 352, 353; fatal effects of, at Mitcham, 460; description of the agaricus glutinosus, 566; five hundred species of the, xxi. 13; employed in the north of Europe to excite intoxication, 14; on the narcotic or stimulating power of three species of, xxv. 123, *note*; on the curative properties of, xxvii. 380; *volvaceus*, analysis of, xxix. 521; *Agaricus Acris*, description of, ibid.
AGATE, on the nebulous light of, xxx. 427.
AGE, observations on advanced, i. 493; regimen recommended in old, 494; change of air beneficial in, iii. 507; on certain effects of, xx. 154; means of attaining old, xxi. 96; on the diseases peculiar to, xxxi. 163.
AGED PATIENTS, observations on, xvi. 93; seamen, on the diseases of, xxii. 373.
AGEN, account of a shower of stones at, xxxiii. 215.
AGRICULTURE, proceedings of the French society of, xxv. 455; its influence on man, xxx. 13; chemistry applied to the improvement of, ibid.
AGRIMONY, botanical description of the common, xv. 269; its astringent qualities, 270; description and virtues of the hemp, or Dutch, xix. 262; its effects in taenia, xxiv. 517.

AGUE, substitutes for the bark in, i. 187; benefit of calomel in, 236; on the causes of, 258; belladonna recommended after, ii. 182; cured by opium, iv. 81; flores arnicae and bark useful in, 85; case of tertian, v. 226; on the treatment of, 231; on the varieties of, vii. 376; in Ireland, observations on, ix. 213; method of treating, by Dr. Marcus, 305; in those of hot climates, x. 85; use of gelatine in, 323; case of a variety of, 427, 541; use of white vitriol in, 496; xi. 129, 246, 247; cuprum ammoniacum in, 247; efficacy of willow bark in, 444; why frequent in the country, xii. 3; good effects of opium in, 4; common plantain said to be beneficial in, 30; efficacy of tartarized antimony in, 340; common masterwort useful in, 371; on the efficacy of animal jelly in, 431; use of emetics in, 499; carraway seeds good in tertian, 556; on zinc vitriol in, xiii. 138; garlic serviceable in, xiv. 65; water-flag good in, 67; frequent in the Isle of Sheppy, 99; efficacy of arsenic in, 100; on sea-bathing in, 101; oak bark recommended in, xv. 69; sedum acre, or pepper stonecrop beneficial in, 265; cured by the ammonia preparata, 333; bark of the sloe recommended in, xvi. 167; root of the tormentil good in, 266; root of common avery *(geum turbanum)* recommended in, 267, 557; on the inspiration of oxygen in, xvii. 447, 449; common germander ministered in, 467; cured by calomel, xix. 15; benefit of chamomile in, 459; instance of one cured by nasal haemorrhage, 53; of another suspended by a scald, ibid.; use of the bark in obstinate cases of, xx. 478; observations on the influence of the air in producing, xxi. 92, 93; virtue of spider's web in, 353, 359; xxii. 94, 96, 218, 369, 370, 376, 457; on the practice of giving the solution of arsenic in, xxi. 441; on the marshy origin of, xxii. 190; how imported into London, 191; extraordinary case of, 496; a burning one in England, xxiii. 283; among the soldiers in Walcheren, xxv. 1; treatment of, 4; on the influence of the moon in, xxvii. 400; account of the jumping, 486; on the use of ligatures at the extremities in, xxviii. 368; on the nature of soils productive of, xxx. 161; on the efficacy of glue in, xxxi. 392; beneficial effects of bleeding in, xxxii. 545; on the frequency of, in marshy districts, xxxiv. 375; the effects of temperature on, 162.

AGUST EARTH, account of the discovery of, vi. 473; xi. 285.

AIKIN, C. R. his experiments on certain sensations of the eye, ii. 227; on the cow-pox, v. 383.

AIR, cold, its benefit in fevers, i. 18; analysis of the atmospherical, 27; nature of inflammable, 68; not a simple body, 167; of sick chambers, on purifying, 245; substance of vital, 269; procured by caustic alkali, 299; its effects on ulcers, 344; on ascertaining the purity of the, 396; composition of atmospheric, ii. 386; proportion of oxygen in the, 387; benefits of change of, in asthma, iii. 507; on the use of vital in malignant fever, 562; quantity of carbonic acid in atmospheric, iv. 83, 179; researches concerning dephlogisticated, 263; observations on the atmospheric, 265; its influence in diseases, 449; received into the absorbent system, 86; carbonic acid in the, 85; on the salubrity of, vi. 10; experiments on land and sea, 203, 359; on the quantity of, in the lungs, 464; its influence on different seeds, vii. 470; on the means of purifying infected, viii. 185; on the decomposition of fixed, 306, 308; method of clearing apartments of foul, x. 575; on contagious matter in the, xi. 82, 85; in hospitals, instructions for purifying, 97, 103; on heat disengaged by the compression of, xii. 575; portable apparatus for purifying the, xiv. 190; on the influence of the, 570; on purifying the, xv. 375; on the foul air of cisterns, 391; method of conveying it in coal mines, xvii. 199; on pestilential constitutions of the, xviii. 86; vitiated by exhalations from plants, 288; effects of vegetation on the, xix. 370; on the change produced in it by the blood, 548; on changes induced in the, 371; on vibrations of the, 403; fire produced by the compression of the, xx. 287; on examining the state of the, xxi. 91; effects of fixed, 385; on changes produced in the, xxii. 15; effects of respiration in, 48; simple method of purifying it in large rooms by fumigation, xxiii. 84; the best remedy for fevers, 117; contained in luminous wood, 388; on the exotics which endure the open, xxv. 248; on the alteration produced in meat by, 252; destitute of solidity and colour, xxviii. 333; on its expansibility, 415; on its influence in smelling, 458, *note*; changes produced by the breathing of animals on the, xxix. 142; discoveries of Mr. Cavendish on factitious, 400; nature of fixed, 401; on the phlogistication of, 404; on swallowing, xxx. 210; explosion of inflammable, 427 ; on exposing wounds to the, xxxi. 450 ; on the chemical influence of the temperature of the, xxxii. 132 ; on its influence in shut sacs, 208 ; its influence in the small-pox, xxxiv. 390 ; in hospitals, on the state of it, 468 ; changes in meat exposed to different kinds of, 474.

AIR BATHS, improvements in, xxi. 85.

AIR BLADDER of fishes, observations on the, ii. 360 ; xxiii. 454.

AIR BUBBLES in dead bodies, on the, xxvi. 405.

AIR PUMP VAPOUR-BATH, account of the, viii. 54 ; beneficial effects of the, ix. 175 ; xii. 67. See *Bath*.

AIX, remarkable phenomenon in the waters of, iv. 458.

AKERLEY, Dr. on the cure of phthisis by mercury, xix. 224.

ALBERS, Dr. his case of preternatural pulsation, xvii. 188 ; his case of hydrophobia healed by bleeding, xxxiv. 426.

ALBERTI discovers the ossa wormiana, ii. 161.

ALBICORE, on the poisonous qualities of the, xxv. 398.

ALBINOS, account of one in France, xviii. 95.

ALBINUS, prices of the anatomical tables of, i. 298; his publication of Eustachius, ii. 65.

ALBUCASIS on lithotomy, xi. 4.

ALBUGO, description of that disease, with a case, viii. 399; on the treatment of, x. 562.

ALBUMEN in vegetables, property of, i. 76; how separated, iii. 371, 372; coagulated by alum, xiv. 264; on the fermenting principle of, xvii. 198; observations on, 302; of the egg, its uses and description, xxiv. 168; in the human blood, xxviii. 67; on the varieties of coagulated, 393; in the pus of ulcers, xxxiv. 399.

ALBUMINOUS matter in vegetables, how ascertained, i. 267 ; tumours in the liver, observations on, xxviii. 5; concretion in the cavity of the thorax, account of, xxx. 73.

ALCAREZ, Dr. Joseph, on the use of oil in yellow fever, xiii. 17.

ALCAZARRAS, porous vases used in Spain, iii. 174.

ALCHEMY, contents of an extraordinary manuscript on, xiv. 91; account of a modern martyr to, xxiv. 431.

ALCOHOL, not essential to the formation of ether, i. 301; method of depriving ether of, iii. 86 ; its being a sedative questioned, viii. 505; experiments to prove the affirmative, ix. 40, 46 ; deoxygenates the blood, xii. 86; retards the healing of wounds, 87 ; distinctions of, xiii. 220 ; its affinity with oxygen, xvi. 533; case of its counteracting the effects of opium, xix. 497; utility of, as a topical application, xxiv. 98 ; its efficacy in burns and scalds, xxiv. 99; how it destroys inflammation, 101 ; effects observable in its evaporation, 102; method of conducting the evaporation of, 103; its effects on the stomach, xxvi. 129, 132 ; xxvii. 4 ; in what way it affects vegetable wax, 73; of wine, its agency in pus, xxv. 332; its effects on the alimentary canal, xxvi. 129; experiments to prove it a production of distillation, 168 ; its effects on belladonna, xxviii. 208 ; procured from fermented liquors without distillation, xxix. 173 ; on a method of freezing, 431 ; of sulphur, discovery of, 409 ; xxx. 79 ; treatment of the human brain with, 56 ; its manner of existence in fermented liquors, 392.

ALCOHOMETER, a new instrument for determining the quantity of spirit in liquids, xxii. 351.

ALCONORQUE, a medicinal tree of uncommon virtue in Martinique, described, xxv. 546.

ALCYONIA, varieties of them found in the Isle of Wight, xxviii. 237 ; discovery of two fossil, xxxi. 340.

ALDER, Dr. Thomas, his account of a work on the disordered states of the lungs, xii. 238 ; his observations on the contagious fever at Jamaica, xv. 40, 43 ; reply to, 560 ; on contagion and fever, xvii. 505 ; xviii. 423 ; observations on quarantine, 497 ; on the source of fevers, 498 ; xix. 113; on endemic diseases, ibid. ; causes of epidemics, 115; on noxious winds, 116 ; on endemic air, 199; on sporadic diseases, 201 ; on the proximate causes of diseases, 334; on the oxygenatic doctrine, 336 ; proofs of his theory, xxi. 102, 296, 302, 449 ; xxii. 36, 42 ; from the practice of physic, 296 ; from the treatment of diseases, 297, 458, 464 ; on the proximate causes of diseases, xxi. 102, 107, 449, 456 ; proofs of his theory from the practice of physic, xxii. 227, 232 ; xxiii. 116, 122, 281, 288, 469, 477 ; proofs from the remote causes of diseases, xxiii. 281, 288, 469, 477.

ALDER TREE, botanical description of the, xii. 35; purgative qualities of the, 222.

ALDERSON, Dr. his case of lusus naturæ, iii. 402 ; on the efficacy of fictitious tractors, iv. 100; account of the influenza at Hull, x. 125 ; on the belief of apparitions, xxiv. 161.

ALDINI, his experiments on a criminal, ix. 195 ; observations on it, 447.

ALDIS, Charles, his case of fissure of the cranium, x. 244; his case of preternatural enlargement of the heart, xxii. 513.

ALE, brewed from the epilobium angustifolium, xiv. 171; brewed from heath, 542.

ALEPPO, account of the cutaneous disease called Mal de, xxxi. 282, 372.

ALEPPO SCAMMONY, analytical essay on the, xxv. 157.

ALETRIS FARINOSA, a remarkable plant of Virginia, described, xxvi. 515.

ALEXANDER THE GREAT exhaled the odour of musk, vi. 342; on his immersion in the Cydnus, xx. 169.

——————, Dr. his experiments on opium, ix. 346.

——————, Mr. his three cases of chorea sancti viti, xv. 125.

ALEXANDRE, M. his method of filtering water, xxii. 439.

ALEXIS, M. proposes a hospital for hydrophobia, iii. 489.

————, of Piedmont, his remedies for the plague, xxxiii. 483.

ALFRED, King, the founder of Oxford, i. 256.

ALGIERS, vaccination introduced in, v. 317.

ALIBERT, M. on the gastric juice, ii. 468; his memoir on odours, 469; on the cholic at Madrid, iii. 167; on medicinal substances used in frictions, ibid; on intermittent fevers, vii. 376.

ALIMENTARY CANAL, use of alkalis in diseases of the, viii. 293; case of disorder in the, by swallowing cotton, xxiii. 431; effects of alcohol on the, xxvi. 129; on the structure and morbid anatomy of the, xxvii 497; of obstructions in the, 501; on fungous tumours in the, 507; on stricture in the, 508; deposition of cartilage and bone on the villous coat of the, 508; derangement of the cellular coats of the, 509; derangement of the muscular coat of the, ibid; on malformations in the, 512; on worms in the, ibid; deposition of albuminous matter in the, xxviii. 3; inflammation of the mucous membrane of the, xxxiv. 18, 100.

ALIMENTS, remarks on the variety of animal, iii. 65, 153, 249.

ALKANET, the juice of its roots used for colouring red, xii. 124.

ALKALI, atmospheric air procured from caustic, i. 299; on vegetable, 300; in the salsulla soda, 301; decomposition of muriated mineral, ibid; vegetable, ii. 182; common salt separated from mineral, 390; overcomes putrefaction, 385; experiments on fixed vegetable, iii. 91; on the mineral, 376; beneficial in convulsive affections, iv. 183; use of vegetable in spasmodic asthma, v. 561; use of the volatile in scarlet fever, ix. 161; benefit of the fixed in hooping cough, 176; bite of a snake cured by volatile, xi. 332, 334, 483; efficacious in hydrophobia, xvii. 293; effects of electricity on fixed, 93; its use in tic doloureux, 388.

ALKALINE MATTER, in dropsical fluids, account of the, xxviii. 62, 63.

—————— SALTS, febrifuge virtues of, i. 275; dissimilarity of fixed vegetable, xi. 464; method of separating them from the acid, xxviii. 425.

—————— SUBSTANCES, antiseptic powers of, ii. 383; their effects on calculous concretions, xvi. 153, 221, 305.

ALKALIS, common in manures, i. 46; their effects on pestilential vapours, i. 98; venereal ulcers cured by, 185; the febrifuge virtues of, 274; on saturating water with, ii. 80; on rendering them caustic, iii. 86; difficulty of decomposing the fixed, 92; in fistulæ lachrymales, 168; good effects of, in poisons, 535, xiv. 478; their use in accidents by lightning, iv. 370; effects of them on stone and gravel, 467; on the medicinal uses of, v. 470, 557; their use in disorders of the stomach and bowels, viii. 289; their efficacy in diseases of the alimentary canal, 293; on the virtues of the fixed, ix. 176; antiseptic qualities of the, x. 475; their effects on poisons, xiv. 478; efficacious in fever, 373; metallic oxyds precipitated in, xv. 295; experiments with the, xvi. 499; account of Mr. Davy's discoveries for the decomposition of fixed, xix, 93, 190; his experiments repeated by Mr. Pepys, 286; a farther exhibition of the discovery, xx. 95; description of the apparatus employed by Mr. Davy, 254; his experiments confirmed in France, 568; on the metallic bases of, xxi 27, 29; method of decomposing the, 350; general view of Mr. Davy's discoveries, xxii. 12, 14; prevent the increase of calculus in the bladder, 52; action of, on animal concretions, xxiii. 47; their effects on rhubarb, xxv. 499; on the metals of fixed, xxvi. 18; on the decomposition of acids, and when united, 168; their effects on the colouring principle of the blood, xxviii. 397; their action on the liquid gum of Botany Bay, xxix. 479; acetic acid, the remedy for poison by, xxxiii. 476.

ALLAN, Mr. on the rocks near Edinburgh, xxvi. 18; on salt of tartar in puerperal fever, 80, 264.

ALLANITE, a new metal, description of the, xxv. 121.

ALLEN, J. his account of Anne Moore, the fasting woman of Tutbury, xxi. 61.

———, John, on blood-letting in continued fever, xxxiv. 249.

———, R. his cases of phthisis treated with digitalis, ix. 263.

———'s Mr. experiments on atmospheric air, xxii. 48.

ALLEY, Dr. George, his case of diabetes mellitus successfully treated by diet and bark, xix. 181.

ALLIUM AMPELOPRASUM, supposed to be the origin of the common leek, xxvi. 486.

——— ARENARIUM, or sand garlic, description and uses of, xiv. 64; A. ursinum, a good remedy for the gravel, 65.

——— CERNUUM, an unusual species of, described, xxiv. 529; *flavium*, characteristics of the, ibid.; *A. cepa*, or the tree onion, description of, xxviii. 164.

——— OBLIQUUM, a very rare species of garlic described, xxvi. 513; *pallens*, account of, xxvii. 83; *paniculatum* described, 259.

ALMANACK, account of a chemical, iv. 80; proposed improvement for an, vi. 336; prospectus of a new, 438.

ALMONDS, oil of sweet, recommended in dolores post partum, vi. 71; beneficial against the tape-worm, 378; cases of prussic acid in the infusion of bitter, x. 95; children poisoned by bitter, xi. 92; experiments with the essential oil of bitter, xxvi. 132, 138; xxvii. 4, 6.

ALOE, on the aromatic wood of the, ii. 293; varieties of the, xxiv. 528; xxv. 187, 370; xxvi. 514; xxvii. 526; xxviii. 163, 340.

ALOES, an efficacious remedy for ascarides, vii. 256; on the extract of socotrine, xxxiv. 496.

ALPINIAS, eight species of those plants described, xxiv. 437.

ALPINUS, on the plants of Egypt, i. 37; on the datisca, 188; on the leprosy of Egypt, iii. 153; on the effects of opium, viii. 393; on the Egyptian method of extracting the stone, xi. 18; his account of the practices of the Egyptians, xxxii. 206.

ALPS, nature and origin of the rocks of the, iii. 375; remarkable plants on the, viii. 282; on the structure of the, xxiii. 525; on the swelled throat peculiar to the inhabitants of the, xxxiii. 283, 437.

ALRUCO SETOSA, description of the, xxviii. 341.

ALSTON, Dr. on the internal virtues of opium, vii. 359.

ALSTROLOMA HUMIFURUM, a native plant of New Holland, described, xxvii. 260.

ALTHÆA, or marsh mallow, botanical description of, xviii. 457; useful in coughs and in calculous complaints, 458.

ALTRŒMERIA EDULIS, a West-Indian plant, described, xxvii. 347.

ALUM, its use in dying cotton, i. 166; discovered in urine, 193; coagulates albumen, xiv. 264.

ALUMINA, specimen of pure, xxx. 84.

ALUMINE, description of the fluat of, iii. 272; its effects on the colouring matter of the blood, xxviii. 398.

ALUMINOUS EARTH, analysis of the, vi. 373.

ALVINE CONCRETIONS, on the nature of human, xxvii. 502.

ALYON, M. experiments on nitrous acid, i. 145; on the theory and practice of chemistry, 204; on the medicinal properties of oxygen, ii. 38.

AMARANTHUS BLITUM, found in the neighbourhood of London, xxv. 93.

AMARYLLIS PURPUREA, description of the, xxvii. 259; *blanda*, a magnificent plant from the Cape of Good Hope, 525.

AMATUS, a Portuguese surgeon, account of, ii. 58.

AMAUROSIS, advantage of exciting vertigo in, i. 24; on the application of cold water in, iv. 179; corrosive sublimate serviceable in, 184; benefit of galvanism in, vii. 247; viii. 323; ix. 131; x. 330; cases of, 138, 142; successfully treated by electricity, xi. 135; xiv. 481; observations on the causes of, 482, 3; benefit of the anemone in, xvii. 74; successfully treated with cayenne pepper, xxii. 353, 355; attended with a diminution of voice and hearing, xxv. 510; reflections in that case, 512; effects of electricity on a case of, xxxiii. 468; case of, cured by active treatment, xxxiv. 425.

AMBER, on the origin of, vii. 92; a curious specimen of, xi. 287.

AMBERGRIS, history and analysis of, xi. 167; found in the lower intestines of whales, xxix. 507.

AMBLYOPIA, instance of the effects of galvanism in, x. 58.

AMBOY, in New Jersey, account of a remarkable disease at, xxviii. 143, 150.

AMELIA FRIGATE, medical observations and cases on-board the, xv. 17.

AMENTIA, characteristics of, vi. 296.

AMENORRHŒA, bleeding injurious in, v. 338; on the causes and treatment of, 392; confused opinions on exposed, xv. 141; objections to Cullen's practice in cases of, xxi. 31; difficulties attending the treatment of, xxii. 46; benefits of electricity in, 323; on the connexion between phthisis pulmonalis and, xxiii. 238, 242; efficacy of rubia tinctorum in, xxvi. 107.

AMERICA, North, ravages of the yellow fever in, i. 78; regulation of the colleges in, 88; materia medica of, 100; splendid flora of, 197; iron ore found in, 198; causes of its unhealthiness, 256; benevolent institutions in, ii. 190; instances of emigration to, vii. 381, 383; on the poisonous serpents of, 478; on the introduction of the yellow fever into, ix. 97; success of vaccination in, 196; history of the autumnal fever in, x. 365; quantity of distilled spirits consumed in, 476; on the population of, 363; on the epidemics of, 309; observations on the yellow fever of, xiv. 79, 313; on vaccination in, xv. 313; elephant in, 486; report of a malignant disease in, xvii. 97; on the climate of, 120; on the progress of medicine in, xviii. 40, 41; on the pestilence of, 121; remarkable pyramids found in, 483; other ancient remains discovered in, 484; ornithology of, 580; preposterous practice of medicine in, xix. 414; the epidemic of that country not contagious, xx. 28; Dr. Chisholm's reasons for the contrary opinion, xxi. 241, 249; bilious plague in, xxiii. 287; mortality in the army of, xxiv. 435; the arts, manners, and superstitions of the aborigines, xxv. 125, 126; medical topography cultivated in, xxvi. 481; philosophical register of, 250; medical topography of, 421; account of a flora of, xxvii. 344; books published in, xxviii. 432; on the spotted fever of, xxix. 14, 328; account of a remarkable disorder in, xxxiii. 27; particulars of an epidemic there in 1812, 92.

———, South, account of travels into the interior of, xiv. 187, 190; earthquakes and volcanic eruptions in, xxiii. 287; account of the diseases of, xxv. 242, 248; general view of the country, xxvi. 9; instances of longevity in, 10; mountainous nature of, 11; account of the plants of, 12; its animals, 13; climate, 14; earthquakes in, xxviii. 161; small-pox extinguished by vaccination in, xxx. 116, 121; account of the animals of, 432.

AMERICAN mineralogical society, proceedings of the, i. 86; philosophical society, letter of, 87; epidemics, history of, xi. 309; observations on the, xiv. 79; xviii. 41, 42; literature, state of, xvi. 479; dispensatory, account of, 570; medical practice censured, xix. 414, 416; army, sickness in the, xxiv. 431.

AMERICANS, on the complexion of the, xxviii. 490.

AMICUS on a diseased testicle, xiv. 529; on chronic rheumatism, xviii. 430.

AMIEL, R. his account of the epidemic at Gibraltar, xxxiv. 301, 309.

AMMONIA, the volatile spirit of, used in cancer, i. 285; on the flowers of, ii. 82; pure, recommended in cases of poison, 182; its utility in pregnancy, 205; incontinence of urine cured by hepatised, i. 218; ii. 288; its alexipharmic powers, 182; its use in gangrene and sphacelus, 342; checks the luminous property of phosphorous, 382, 389; preparation of the sulphat of, iii. 169; obtained from oxygenated pomatum, iv. 470; acetated water of, recommended in gout, vi. 456; on the lithat of, 532; generated in urine, vii. 475; efficacious in ague, xv. 333; its effects in tic douloureux, xvii. 579; its use in narcotic poisons, xviii. 19; oxygen, an alkalizing principle of, xx. 96; scrofula relieved by the carbonate of, xxviii. 17; observations on the liquor of, in the Pharmacopœia Londinensis, 228; on the aromatic spirit of, 229, 231; why it yields little flavour, 460; on the spontaneous formation of, xxx. 260; its effects in preventing contagion, xxxi. 83; a remedy for the bite of vipers, 172; benefits of the muriate of, in hospital gangrene, 518; preparata of the New Pharmacopœia, on the names of, xxxii. 11; coagulates the blood in the veins, xxxiii. 475; on the preparation of the sub-carbonate of, xxxiv. 491.

AMMONIAC, sal, its virtues in cancer, i. 285; combined with oxygenated muriatic acid, 300; crystals found in the decomposition of, 301; phenomenon observed in the martial flowers of, ii. 82; analysis of the urat of, ix. 492; use of the solution of it in vinegar, xiii. 497; injury arising from the distillation of, xvi. 105.

AMMONIACAL GAS, singular experiments on, xxix. 516.

AMMONIACO MAGNESIAN PHOSPHAT, analysis of the, ix. 494.

AMMONIACO NITRATE OF SILVER, preparation of, xxviii. 286.
AMMONIACUM, its effects in chorea, i. 22; beneficial in epilepsy, 23; formed in urine, 193; combined with muriatic acid, 300; loses its efficacy in pills, xxii. 360.
AMMONIATED IRON, preparation of the, xii. 192.
AMMONUS, the lithotomist, account of, xi. 19.
AMOMUM, four species of, enumerated, xxix. 436.
AMPHIBIA, observations on, xiii. 370; on the seat of the heart in, 430.
AMPUTATION, on the application of the tourniquet in, i. 23; on that of the uterus, 392; uncommon occurrence after, iii. 4; of the articular extremities, iv. 406; of the hand, 415; of the thigh, case of, viii. 3; in case of aneurism, observations on, ix. 180; another case successfully performed, 499; of the extremities of the bones in caries of the joints, x. 557; reasons for avoiding, xi. 68; in cases of gun-shot wounds, 393; historical account of the practice of, xiii. 96, 103; on the introduction of the ligature in, xiv. 40, 103; case of the operation at the shoulder joint, 108, 111; on the article Amputation in the New Cyclopædia, 111; on the ligature of arteries after, xvi. 79; of the uterus mistaken for a polypus, 472; of the hip-joint, xvii. 469; on the difficulties attending, 537; fatal case of, xxii. 120; case of lock-jaw cured by, 123; at the joint, cases of, with observations, xxiii. 108, 111; method of securing the arteries in, 253; in stopping the current of the blood in, 329, 479; directions in sawing the bone, xxv. 146; at the shoulder, successfully treated, xxvii. 378; fatal termination of, xxviii. 89; causes of it, 91; method of preventing it, 92; on the difference between the English and French methods of, 370; objections to the frequency of, xxx. 461; of the thigh, or the circular, xxxiii. 303; of the arm, at its upper anticulation, 400; superiority of the English practice in, xxxiv. 524; on the mode of doing it at the joint, 525.
AMUSEMENTS, on the utility of public, xiii. 115.
AMYRIS GILEADENSIS, description of the, i. 36.
ANALOGY, on reasoning from, xvi. 115, 423.
ANACAMPSEROS FILAMENTOSA, and arachnoides, separated from the genus portulaca, xxvi. 485.
ANASARCA, on the treatment of, i. 58; efficacy of green broom in, 383; of the liver, singular case of, ii. 170; of the lungs, case of, treated with digitalis, iii. 202; appearances on opening a person who died of, 288; case of, with hydrops pulmonum, vii. 406; pulmonum, case of, xvi. 354; milk-wort serviceable in, xix. 73; efficacy of tobacco in, xxv. 332; supertartrite of potash recommended in, xxvii. 340; case of cure in a man above eighty years old, xxxi. 432.
ANAS MOSCHATA, its peculiar properties, vi. 345.
——— NIGRA, or the scoter found to breed in the Isle of Man, xxv. 374.
ANASTOMOSIS, case of aneurism by, xxviii. 56.
ANATHÆSIA, history of a case of, xxx. 167.
ANATOMICAL Nomenclature, plans for an improved, ii. 479; subject, a singular, 480; preparations, method of preserving, vi. 274; injection, a new species of, xiv. 52; preparation of, in wax described, xxxiv. 186.
ANATOMY, history of discoveries in, ii. 60, 160, 281, 368, 459; iii. 72, 156, 256, 356; iv. 340; importance of a knowledge of, ii. 142; advantage of morbid, iii. 17; advice on the study of, iv. 461; on the physiological application of general, vii. 472; observations on vegetable, 557; general view of comparative, viii. 365, 467; the study of it recommended in education, ix. 477; of expression, account of a work on the, xviii. 8; advantages of comparative, xx. 2; table of natural arrangement of, 44, 45; a threefold division of, 101; on the term morbid, as applied to, 535; on the study of, xxi. 181; its study vindicated from the charge of materialism, xxii. 3; beneficial to other sciences, 4; progress of, 8; of the human body, tabular views of, xxiii. 514; limits to the study of, xxvi. 6; on the importance of surgical, 388; of the leech, 410; advantages of studying morbid, xxvii. 494; definition of, xxviii. 2; of the brain, 12; of plants, 319; papers in the Philosophical Transactions on, 328; studied by the Rajah of Tanjore, xxx. 219; of insects, xxxi. 515; of the nervous system, xxxiii. 228; of the brain in maniacs, 139.
ANCHYLOSIS relieved by warm oil, vi. 71; remarkable case of universal, viii. 284, 395; observations on, xxi. 424.
ANCIENTS, observations on the surgery of the, xxxii. 111, 297.
ANDALUSIA, account of the pestilential disorders of, xxxiv. 498.
ANDERSON, Dr. Alexander, his anatomical engravings, ii. 190.

ANDERSON, Dr. Charles, his case of severe affection of the stomach, xxvii. 167.

————, Dr. James, on vaccination in India, xiii. 283, 381; his correspondence on a plan for exterminating the small-pox, xiv. 409; xv. 525; on the Guinea worm, xvii. 171; on the ichneumon, 172; his letter on the cow-pox, 540; xx. 25.

————, Dr. clove-seed propagated in the Island of St. Vincent, by, xxvii. 78.

————, Mr. his observations and cases of tumour of the brain, xiv. 81; on vaccination, xv. 137.

ANDRE, Mr. on inoculated cases at Petworth, iii. 412.

ANDRE, M. the supposed author of the name tic douloureux, as applied to a disease of the face, xxxi. 180.

ANDREWS', St. state of the University of, xxx. 267, 273; fees for graduation at, 468; observation on, xxxi. 92.

————, Dr. his cases of varicose veins, xvii. 297, 299, 305; of popliteal aneurism, 302; on lunar caustic in strictures of the urethra and œsophagus, xviii. 380; on the accommodations of Madeira, xix. 19; his account of the success of vaccination in that island, xxiii. 85, 93, 104; history of an enlarged leg, xxv. 400.

ANDROSACE coronopifolia, description of that plant, xxvii. 347.

ANEL, Dominic, his method of operating on the fistula lachrymalis, xxvi. 228; his treatment of aneurism, 241.

ANEMOMETER, for measuring the strength of the wind, xii. 383.

ANEMONE, its introduction into medicine, i. 158; botanical description of, xvii. 73; efficacious in diseases of the eye, 74; occasions dysentery in sheep, xxi. 12.

ANEURISM of the aorta, on digitalis in, i. 331; cause of, 400; observation on a case of, ii. 481; on the operation for the, iii. 531; on the causes and treatment of, iv. 367; from a puncture of the humoral artery, vi. 535; history of a fatal case of, vii. 97; on tying the artery in, viii. 1; condition of the artery in, ix. 88; history and causes of, 179; case of caries of the bone combined with, 180; on amputation in, ibid.; method of operating in, 181; anomalous species of, ibid.; of the aorta, case of, xi. 305; general view of its causes, 306, 309; fatal case of, 396; of the aorta, by Dr. Rodman, xii. 235; cases of, 275; on that article in the new Cyclopædia, xiv. 111; practice of the Greeks in, 112; symptoms and effects of, xv. 476; observations on spurious, xvi. 87; remarkable case of, xvii. 5; the tibialis antica, 7; case of femoral, 190; Dr. Andrews's case of popliteal, 302; case of popliteal, in the royal hospital at Deal, 307; account of the operation in, 308; remarks on the case, 310; remarks on Dr. Cayley's case of, 435; on caustic in, 437; on a spurious case of, 471; farther observations on it, in the way of vindication, xviii. 48; on a case of popliteal, 158; case of doubtful, 300; further observations on a suspicious case of, 374; on the utility of a truss for compressing the artery in, 375; on varicose, 376; explanation on Dr. Cayley's case of, 432; case of the high operation in popliteal, 475; enlarged and tuberculated liver mistaken for, 485; appearances on the dissection, 488; case of femoral, successfully healed, 508; on compression in diffused, xix. 234; remarkable flow of blood in a case of, xx. 264, 266; case of a man dying under the operation for popliteal, 430; palpitations of the heart in consequence of, xxi. 337; of the femoral artery, Dr. Hosack's case of, 424; objections to the general treatment of, 537; of the carotid artery, cases of, xxii. 67, 69; of the thoracic aorta, on the, 505; Mr. Abernethy's method of securing the arteries in, xxiii. 242, 253; of the thoracic aorta, on the, xxiv. 27; cases of venous, 285, 302; cases of, xxv. 356; on the modern improvements in the treatment of, xxvi. 33; new method for operating for popliteal, 241; cases of, 242, 244; of the aorta that burst into the pericardium, 251; on the operation for popliteal, xxvii. 29; case of popliteal, in which the femoral artery was tied three times, 177, 179; case of inguinal, 418; case of, by anastomosis, xxviii. 56; of the aorta, case of dysphagia produced by, 151; notices of several important operations in, 248; at the origin of the aorta, xxix. 52; observations on the case, 57; fatal case of one mistaken for an abscess, xxxi. 477; case of the cure of, xxxii. 26; treatment of, by Paulus Ægineta, 304; of the heart, case of the, with perforation in the walls of the auricle, 389; on dividing the inner coat of the artery in, 444; of the heart, pericarditis, and peritonitis hepatica, 448; operations for, xxxiii. 158; an operation on a large inguinal, 167; of the aorta, remarkable case of, 265, 449; case of subclavian terminating favourably, 377; in the right subclavian artery, cases of, 221, 222; of the aorta, with a slight irregularity of pulse, 265; case of popliteal cured by compression of the artery, xxxiv. 116; case of axillary, in which the subclavian artery was tied, 118; cases of, in Sicily, 421; on the general formation of, 141; on the doctrine of the ancients concerning, 142; the opinion of Scarpa on, 143; on the formation of a tumour in, 144; on the surgical treatment of, 149.

ANEURISMAL NEEDLES, on the claim to the invention of two, xxv. 32, 238.

ANGELICA, garden, botanical description of, xii. 364; its aromatic and carminative powers, 365.—*Angelica Sylvestris*, description of the, 365; chemical researches on the, xxviii. 386, 402.

ANGER, remarkable effects of, in a boy, ix. 288; epilepsy following a paroxysm of, xiv. 275.

ANGERSTEIN, Mr. letter from Lord Hardwicke to, ix. 540.

ANGINA LARYNGITIS, two cases of, xxxii. 166; case of, xxxiv. 255.

——— MALIGNA, proposed plan for preventing the, v. 424; observations on the, ix. 249; utility of muriatic acid in, xi. 292; improprieties in the mode of treating, xii. 27; history of an extraordinary species of, in Italy, Switzerland, and Germany, xxiii. 285.

——— PECTORIS, use of the nitrate of silver in, i. 181; symptoms of the, iii. 390; causes, 476; Dr. Cullen's view of, 478; case of, in a lady, iv. 30; observations on it, 32; successful cases of, by Dr. Cappe, 221; Mr. Goodwin's cases of, vi. 320; history of a fatal one, by Dr. Patterson, x. 63, 65; benefit of issues in, xv. 380; remarkable case of, in a negro, xvi. 487; dissection, 489; case of, xvii. 9; the subject of a prize question at Paris, xxiii. 260; on the symptoms usually, but not always, attending, xxxi. 161.

——— POLYPOSA, case of a disease resembling the, vi. 94.

——— TRACHEALIS, fatal case of, viii. 203.

ANGUIS FRAGILIS, description of the, xxi. 6.

ANGUS, Charles, his trial for the murder of Margaret Burns, xxi. 336, 343; observations on the medical evidence, 344, 348, 399, 426, 431; an allusion to his case, xxxii. 327.

ANGUSTURA BARK, beneficial in ulcers, i. 502; description and analysis of, xi. 566; mischief arising from a poisonous species of, xvii. 428; on detecting the spurious, 429; characteristics of the genuine, xx. 21; chemical account of it, with the means of ascertaining the true kind, xxii. 144; action of boiling water on, 146; table of the effects produced by the three kinds of, 149.

ANIMAL, discovery of an extraordinary one in New South Wales, iv. 83; account of a fossil, xv. 391; description of an uncommonly small, xvii. 583; of the tribe of aurochs discovered, xxiv. 87.

——— BEZOARDIC RESIN, history and characters of, ix. 498.

——— BODIES, effects of electricity on dead, i. 200; influence of chemistry on, vii. 261, 460; analogy between vegetables and, ix. 583; means of preserving them from putrefaction, xi. 358; source of oxygen in, xiii. 107; on peculiarities in the functions of, xviii. 36; on the effluvia of dead, xxiv. 422, 424; on the nature of fat in, xxxiv. 435.

——— CHEMISTRY, observations on, vi. 416; on the progress of, xxx. 8, 12.

——— CONCRETIONS, on the numbers and characters of, ix. 490; description of various kinds of, xvi. 3.

——— ECONOMY, on the effects of carbonic gas on, viii. 189; effects of heat on the, xxiii. 307, 311; importance of the nervous system to the, xxxiii. 198.

——— FLESH, employment of potash with, x. 475; converted into adipocire, xxix. 507; xxxi. 45.

——— FLUIDS, galvanic experiments on the, xi. 287; essays on the analysis of, xiv. 263; xv. 280; general view of the composition of, xxx. 9, 233.

——— FOOD, observations on the nutritive qualities of, iii. 65; method of preserving by potash, x. 475; not natural to man, xxi. 510, 512; reasons for not eating, xxvi. 310; vindication of the use of it, xxvii. 47; history of abstinence from, 312.

——— HEAT, observations on, viii. 300; in typhus, xii. 244; on Dr. Crawford's theory, xxi. 67; objections to, 68; experiments to ascertain the real source of, 69, 70; influence of the brain in the generation of, xxvi. 58, 68; on the chemical theory of, xxvii. 9; influence of the brain in generating, xxix. 141; experiments in confirmation of Dr. Black's theory of, xxxi. 339; on the production of, xxix. 441; dependent on respiration, 443; experiments on, xxxi. 339; observations on the causes of, xxxii. 133; on the production of it by respiration, 233; refutation of the opinions contained in the preceding essay, 295; Dr. Davy's experiments on, xxxiii. 113; referred to vital action, 120.

ANIMAL IMPREGNATION, instance of artificial, i. 47; on the theories concerning, ii. 152, 242; on the proximate cause of, vi. 254.

——— JELLY, its efficacy in intermittents, xii. 431; analysis of, xiv. 294; its presence in the tanning principle, 395.

——— KINGDOM, variety of aliments produced by the, iii. 65, 153, 249.

——— LIFE, real nature of, i. 132; on the principle of, ii. 242, 321; xxxiii. 278; analysis of lectures on, iii. 184, 283; observations on the doctrine of, 272; on the functions of, vii. 465; on the principal functions of, xxvii. 8; inquiry into the properties of matter in relation to, xxviii. 330; observations on the principle of, xxxi. 417; xxxii. 56; on the supply necessary to the support of, xxxi. 431.

——— LIGHT, inquiries into, xxv. 409, 424, 512, 518.

——— MATTER, action of heat and caustic on, x. 26.

——— NATURE, on the laws of, iii. 487.

——— SECRETIONS, on the, xxii. 174.

——— SUBSTANCES, liquid obtained in, i. 85; account of a new one, ii. 298; on the decomposition of, xvi. 103; xxv. 508; method of preserving them for several years, xxvii. 75.

ANIMALCULÆ, observations on their effects, x. 139; cancer ascribed to, xvi. 403; contagious diseases supposed to originate with, xxiv. 22, 27; on diseases occasioned by, xxvii. 379.

ANIMALS, instances of the artificial impregnation of, i. 47; on the mechanical motion of, 97; on the respiration of, 170; on the gradation observed in, 405; peculiarity in the arteries of, 490; on experiments on, ii. 80; philosophy of, iii. 388; on the odours exhaled by living, vi. 339, 439; on the classification of, viii. 368; division of vertebral and invertebral, 369; on the thinking faculty of, 467; experiments on, with hydrogenous sulphurated gas, 522; on the contraction of the muscles in, x. 53; cause of motion and sensation in, 253; on the action of opium in, xi. 145; effects of metals on, 287; on preserving the dead bodies of, 358; on the pustulous eruptions of, 431; on the salivary glands in vertebrated, xii. 548; endued with inherent electricity, xiii. 85; observations on amphibious, 370, 430; on the pulsations of, xvii. 93; on the connexion between plants and, xix. 381; table of natural arrangement of the, xx. 44; on cruelty to, xxi. 335; effects of galvanism on living, xxii. 313, 323; on the degree of heat supported by, xxiii. 309; on the torpidity of, 502; xxiv. 8; observations on the light of living, xxv. 409, 512, 518; analogy between plants and, xxvi. 8; on the distribution of the blood in the bodies of, 83; cruelty of experiments on living, xxvii. 7; on the respiration of young, 375; practical rules for the treatment of parturient, xxix. 66; changes produced in the air by the breathing of, 142; on the law of nature which regulates their propagation, 503; fat necessary to the growth of, 508; on the temperature of, 518; on injecting the blood vessels of, xxx. 91; new classification of, 430; of America described, 432; experiments on the principle of life in, 58; on the analogies observed between plants and, 64; observations on the articulation of, 517; on the formation of fat in the intestines of, xxxi. 43; examination of the cæca of, 47; experiments for the preservation of the flesh of, xxxii. 435; cruelty of experiments on living, xxxiii. 401; on the peculiar formation of some, 489; on the organ of meekness in, 501; worms found in the arteries of some, xxxiv. 149; connexion between the extravascular and vascular parts of, 165; on the quantity of air required by, 171.

ANIMATION, suspended, on the use of vomits in, i. 59; immediately after birth, case of, iii. 222; the art of recovering, vi. 85; remarkable case of, 117; on administering fluids in, 118; effects of electricity in, 119; on the restoration of, ix. 291; case of one by hanging, xvi. 529; recovery of, 535; by drowning, xx. 235, 236; beneficial effects of oxygen gas in, xxxii. 86.

ANIME, on the uses of gum, xxii. 360.

ANJUGA, or ground pine, botanical description and medical virtues of, xvii. 466.

ANKERS, Mr. on purulent ophthalmia, xxiii. 155.

ANKLE JOINTS, case of compound fracture of the, xviii. 162.

ANNESLEA SPINOSA, an Indian plant discovered by Lord Valentia, xxv. 549.

ANNESLEY, Mr. on a new species of rubus in Wales, xxix. 411.

ANNULAR SAW, description of an, xxxiii. 189.

ANŒMIA, a species of chlorosis, peculiar to workmen in some coal mines, xvii. 472.

**ANOREXY**, on unusual cases of, xxx. 388.

**ANORREXIA MORIABILIS**, the extensive signification of, xxii. 338.

**ANSELMI**, Dr. of Turin, his remarkable account of a snake, xiii. 287.

**ANSTEY**, Mr. his ode to Dr. Jenner, xi. 40.

**ANTHEMIS PYRETHRUM**, nature and medicinal properties of the, v. 329.

**ANTHERICUM ANNUUM**, description of the, xxvii. 526.

———— **PUGIONIFORME**, ib.

———— **LONGISCAPUM**, found to be the same plant with the asphodeloides of Aiton, xxv. 94.

**ANTHON'S**, Dr. observations on yellow fever, xvii. 126.

**ANTHRACOMETER**, an instrument for ascertaining carbonic acid in the air, described, iv. 179.

**ANTHYLLIS**, or kidney vetch, description and virtues of the, xix. 76.

**ANTICHRIST**, extraordinary opinion concerning, vii. 110.

**ANTIDOTES** to various poisons enumerated, xxxiii. 400, 473.

**ANTIJACOBIN REVIEW**, extracts from the, xvii. 152.

**ANTIPHLOGISTIC THEORY** described, i. 29.

**ANTIMONIAL POWDER**, its effects in the hydrocephalus internus, xvii. 551, 558.

———— **SOLUTION**, form of preparing the, xxxiv. 267; its benefit in cases treated as syphilitic, 289.

**ANTIMONIALS**, recommended in rheumatism, xxx. 365.

**ANTIMONIUM TARTARISATUM**, on the uses of, ii. 34; useful in bilious fever, vi. 311; ineffectual in ophthalmia, xxxi. 17; observations on its use in the same, 189, 355; improved method of preparing, xxxiii. 484.

**ANTIMONY**, on the external use of tartarized, ii. 34; method of obtaining the tincture of, 75; beneficial in cynanche trachealis, 146; on the oxyd of, iii. 580; composition of the glass of, v. 199; tartarized, its use in preventing bilious fever, vi. 311; its effects in angina pectoris, 321; experiments on, to ascertain the composition of James's Powder, 518; method of preparing the glass of, viii. 92; on the distillation of butter of, xi. 497; experiments in preparing tartrite of, 499, 505; on the submuriat of, xiii. 225; on the muriat of, 447; on the uses of, xvi. 428; analysis of the powder of, xxi. 438; injurious in fever, xxii. 41; glass of lead sold in London, instead of the glass of, xxiii. 439; form of preparing the regulus of, xxv. 315; recommended in hooping-cough, xxvii. 102; its benefit in fever, 297; experiments with the tartrite of, xxviii. 126; its use in ophthalmia, xxix. 452; xxx. 46; objections to it, xxxi. 17; vindication of it, 189; its benefit in typhus, xxx. 321; on the acids of, 344; on the oxide of, xxxiv. 492; on the preparation of the tartarized, xxxiii. 338; xxxiv. 493; on the composition of the powder of, xxxiv. 494.

**ANTIRRHINUM**, or yellow toad flax, good for the piles, dropsy, and jaundice, xviii. 171.

**ANTISCORBUTIC**, an excellent, xvi. 263.

———— **SYRUP**, composition of the depurating, xxi. 290, *note*.

**ANTISEPTICS**, classes of, vii. 565.

**ANTISORIC** preparations, observations on, viii. 113.

**ANTI-VACCINARIAN SOCIETY**, remarks on the, xvi. 27, 476; want of candour in the, xix. 426.

**ANTYLLUS**, on the surgical practice of, x. 7.

**ANUS**, child born with an imperforate, ii. 479; method of forming an artificial one in crural hernia, xi. 261; case of a female child with an imperforated, xii. 236; case of malformation of the, xviii. 297; two French cases of imperforate, xix. 421, 2; on the principal diseases of the, xxiv. 415; male child born with an imperforated, 518; case of preternatural, xxv. 449; on closing an artificial, xxviii. 9; cases of prolapsus of the, xxix. 84; case of the intestines protruded from the, xxxi. 161; on the principal diseases of the, xxxiii. 236.

**AORTA**, discovery of the course of the, ii. 459; error of the old anatomists on the, 460; cases of aneurism of the, 481; xi. 305; xii. 235; on the ossification of the, xvi. 452; case of ruptured, xviii. 391; extraordinary instance of aneurism of the, xx. 264; on aneurism of the thoracic, xxii. 505; xxiv. 27; experiments on that of a living dog, xxvi. 83; case of aneurism of the, which burst into the pericardium, 251; dysphagia produced by the aneurism of the, xxviii. 151; case of aneurism of the, origin of the, xxix. 52; observations on the, 57; on a strong pulsation of the, xxxi. 159; singularity of the, in an infant, xxxii. 515; case of obstructed, xxxiii. 506; aneurism of the, with a slight irregularity of the pulse, 265; remarks on the case, 272; farther account of it, 449; account of the dissection of a young woman who died of an aneurism of the, xxxiv. 146.

from noxious air, viii. 575; care to be used in lighting, xiv. 14; how to clear them from flies, xvi. 190; improved mode of heating, xxiii. 152; plan for adopting a graduated temperature in, 262.

APES, dissections of several, ii. 480; account of the inoculation of one, viii. 272; on the analogy between the human species and, xxi. 512.

APHONIA, galvanism beneficial in, viii. 256; use of moxas in, ix. 389; cured by purgatives, xxii. 252; effects of electricity in, xxxi. 39.

APHORISMS, nature of medical, xxviii. 19.

APHRODISIACS, account of pretended, xxviii. 475.

APHTHÆ, symptoms and treatment of, ii. 15; defined, v. 247; ascribed to ulcerations from hot food, xiii. 298; caution to nurses respecting, xx. 94; liverwort recommended in, 271; described as ulcuscles, xxvii. 103.

APHTHOUS ULCERS, in the mouth, remedy for, xiv. 546; benefit of houseleek in, xvi. 76.

APOPLEXIA HYDROCEPHALICA, a case of, with remarks, v. 121, 124; case of, by Mr. Ricards, 341; on mercury in, 345; viii. 62; by Mr. Weaver, xv. 322; observations on the treatment of that case, xvi. 117, 119; defence of it, 335, 338.

APOPLEXY, in children, bleeding and emetics recommended in, i. 392; on the use of phosphorus in, v. 396; statement of a case of, by William Crowfoot, vi. 549; answer to that statement, 552; cases of, by H. Davies, vii. 74; Dr. Langslow, in explanation, on the subject of, 77; observations on his opinions, 89; remarks on Crowfoot's and Langslow's statements, by G. Wilkinson, 138; Dr. Girdlestone's explanation on, 149; Mr. Crowfoot in explanation, 150; James Atkinson's observations on the dispute, 151, 363, 441; Dr. Mossman on the controversy, 205; Mr. Davies, in explanation, on, 259; Dr. Langslow in reply to Dr. Girdlestone, 273; to Mr. Wilkinson, 275; to Mr. Atkinson, 277; Pyrrho on, 328, 515; Mr. Crowfoot in answer to Dr. Mossman, 361; reflexions arising from the controversy, 397; Mr. Wilkinson in vindication, 436; Dr. Langslow's rejoinder, 445; Philo Medicus on, 454; observations on the causes of, by H. H. 455; Mr. Clement's relation of a case of, 513; general review of the controversy, viii. 64; on emetics in, 69; remarks on, 77; observations on, by Galen, 80; on extravasation in, 115; fatal case of epileptic, 209; sketch of the controversy, 379; observations on, illustrated by cases, ix. 320; on the use of emetics in, 327, 411; on the propriety of bleeding in, ibid; caution respecting, 334; on the diversity of opinions concerning, 409; on emetics in, xi. 79; on the varieties of, as requiring different treatment, 348; allied to gout, xiii. 146; danger of bleeding in, xiv. 379; observations on, xvi. 530; cautions to persons liable to, 380; Dr. Spalding's case of, xvii. 423; extraordinary appearance of the blood in, 425; observations on, xxi. 286; inefficacy of galvanism, in, xxii. 321; case of fatal, in which blood was effused into the medulla oblongata, xxiv. 92; from extravasation of blood upon the brain, 95; on the use of tobacco in, xxv. 229; on emetics in, 230; case of recovery from, xxvi. 181; synopsis of the different kinds of, xxvii. 152, 155; history and anatomy of, xxviii. 21; cases of, with observations, 211; dissections of subjects in, 212; state of the brain in, 213; state of the liver in, 214; anatomy of, 215; proximate causes of, 216; treatment of, 218; objections to bleeding in, 371, 375; sometimes originates in an affection of the stomach, xxix. 15; translation of Portal's treatise on the nature and treatment of, xxx. 193, 370; fatal case of, xxxi. 93; its frequency in London, xxxiii. 519; advice to those who may exhibit symptoms of, xxxiv. 112.

APOTHECARIES, history of the proceedings of the, xi. 474; curious particulars of ancient, xii. 429; their numbers originally, 430; caution to them on selling medicines, xxiii. 67; on the practice of, 139; degraded condition of, xxv. 393; hardships peculiar to, xxvi. 385; their practice in overloading patients with medicine, 455; pleasant anecdote of one, 456; questions on the legal claims of, xxvii. 52, 53; formerly partners with physicians, 116; meeting of the London, xxviii. 403; on the profession of, 405; on the utility of, 408; plan for the benefit of, 410; report of the committee of, 425; resolution of the meeting of, 513; observations on the efforts of, xxix. 2, 9; second report of, 78; defects in their education, 118; objections to their scheme, 130; defence of their conduct, 132, 202, 279, 393, 395; address of the committee of, 165; outlines of their plan of reform, 167; list of subscribers to it, 168, 258, 340; attempt to reconcile them and the physicians, 275; complaints of their encroachments, 276; inattention to their apprentices, 290; impolicy of their bill, 301; their condition in former reigns, 310; instances of their being punished for prescribing medicines, 311; address, explanatory of their motives, 346; list of the committee, 349; observations on the education of, 393; on the necessity of an act in their behalf, 395; advice to the pupils of, 445,495; strictures on the bill,456,467; their association a system of medical police, xxx. 3; on the opposition to their bill, 4; amendments in the plan, 6; on the profession of, 48; vindication of the, 178; advice to the committee of, 179; resolutions of the associated body of, 260; on the powers of, 268; of Ireland, 286; amended report of the London committee of, 307; review of their bill, 385; appendix of correspondence to the report of the, 385, 472; remarks on the qualifications of, 467; their proposed bill injurious to other practitioners, 479; introduction of their bill into the House of Commons, 522; on the degradation of their apprentices, xxxi. 99; their reports of diseases, 171, 257, 346, 433, 522; xxxii. 82, 170; suggestions to them for a new bill, xxxi. 229; third report of the committee of, 259; anecdote of an ignorant one, 477; general meeting of, 511; fourth report of the, 512; fifth report of the general committee of, xxxiii. 157; improvements in the hall of the company of, 336; general view of their bill, 422; their bill passed in the House of Lords, xxxiv. 169; regulations for the examination of, 251; extracts from the act for regulating the practice of, 252; abstracts of the act, 344; observations on it, 346.

APPARATUS ALTUS, for the stone, description of the, xi. 5.

APPARATUS, account of one for applying gases to wounds, i. 430; for impregnating liquids, ii. 80; for bleaching, 297; for the relief of odontalgia, iii. 403; description of the resuscitative, vi. 84; for injection in hydrocele, viii. 175; account of a pneumatic, 382; description of the galvanic one of Mr. Pepy's, ix. 390; of a steam, xii. 575; a portable one for purifying the air, xiv. 190; description of Mr. Davy's galvanic, xx. 254; xxi. 527; for determining the quantity of spirit in, xxii. 351; experiments with the voltaic, 174; electrical, xxviii. 265; one for examining the effects of respiration, xxix. 142; for condensing the gases, 507; for ascertaining the quantity of heat, 512; Mr. Children's galvanic, xxx. 426; for the local application of steam, xxxii. 18; to prevent breathing noxious air, 80; for condensing heat in distillation, xxxiv. 114.

APPARITIONS, on the belief of, xxiv. 161.

APPERT, M. his method of preserving animal and vegetable substances, xxvii. 75.

APPETITE, history of a monstrous, iii. 209; case of chronic dysphagia originating from a ravenous, 366; an uncommon instance of voracious, viii. 284.

APPLES, on the acid of, i. 159; how to preserve them from frost, 297; on the fermentation of the juice of, xi. 415.

APPLE TREES, method of preserving them from insects, i. 198, 304.

APPOO, Don Juan, a Cingalese physician, xxvi. 491.

APPRENTICES, on the hardships of medical, xxix. 290, 445, 495; xxxi. 99; xxxiv. 253, 346, 347, 432, 466; advice to them on their studies, 432.

AQUA AMMONIÆ ACETATA, cheap method of preparing the, ii. 290; its efficacy in tic doloureux, xvii. 579.

——— KALI PURI, experiments with it on urinary calculi, xvi. 502.

——— LAUROCERASI, method of preparing, ix. 288; prussiac acid contained in, x. 95.

——— TOPHANA, supposed composition of that poison, xvii. 255.

AQUARIUM SITIENS, a native plant of Andalusia, described, xxvii. 398.

AQUEOUS HUMOUR in the eyes, on the evacuation of the, xvii. 193; observations on, xxxi. 504.

——— INJECTION in hydrocele, its benefits, xxvi. 110.

AQUILARIA OVATA, generic character of, ii. 294.

ARABIA, ravages of the small-pox in, xviii. 192.

ARABIAN SEA, on the luminousness of the, xxv. 41.

——— HORSES, account of the grease in, xvi. 322.

ARABIANS, their knowledge of chemistry, v. 174; cow-pox said to be known to them, xi. 326; superstition of the modern, xxii. 196; the inventors of distillation, 271; on the leprosy of the, xxv. 84.

ARABIC MANUSCRIPTS on the Small Pox, xi. 326.

ARANZI, Jul. Cæs. anatomical discoveries of, ii. 67, 161, 166, 368, 372; iii. 258.

ARBOR INTEGRA ET RUINOSA, HOMINIS, a curious medical work of the sixteenth century, account of, viii. 401.

ARBUTUS ALPINIA, or mountain strawberry, description of the, xv. 72; description of the trailing, 261; its effects on the urinary organs, 262.

ARCE, Franciscde, a Spanish surgeon, account of, ii. 364.

ARCH, in crural hernia, how divided, xx. 259.

ARCHER, Dr. John, on seneka-root in cynanche trachealis, i. 83, 106.

———, Dr. Thomas, his case of difficult parturition, i. 154.

ARCTOLIS GLUTINOSA, a new species of plant, described, xxv. 94.

ARCTOPUS ECHINATUS, medicinal virtues of the, xxii. 261.

ARDEA MAJOR, or common heron, description of the, xxv. 286.

ARDEA GRUS, query concerning the, xxvii. 380.

ARDENT, instance of an endemic cynanche parotidea, on board the, xx. 180.

ARDISIA ELEGANS, a beautiful plant in Pulo Penang, described, xxv. 550.

ARDRUSSET, M. on the effects of ether in strangulated hernia, xxv. 28.

AREOLA, observations on the vaccine, xx. 338; in the cow-pox, defined, xxxiv. 396.

AREOLAR disposition of the body, on the, xvi. 518.

ARETÆUS on bronchotomy, x. 7; his account of the morbus cardiacus, xv. 378; on the elephantiasis, xxxii. 141.

ARGAND'S LAMPS, account of, xxiv. 14.

ARGEMONE MEXICANA, yellow thistle, its medicinal virtues, xxxii. 111.

ARGENTUM NITRATUM, its effects in epilepsy, i. 181; cases of its use in the same disorder, 222; its application to ulcers, 430; Dr. Mudie's case of its success in epilepsy, ii. 70; another by Dr. White, 173; instances of its failure, by Dr. Magennis, iv. 417; on its anti-epileptic power, with cases, v. 435, 503; useful in the bites of serpents, xi. 486; successfully used in hooping-cough, xvi. 16; its efficacy in ulcers, xix, 98; applied to a stricture of the urethra, xxii. 341; a test for detecting arsenic, xxviii. 66, 70, 109, 187, 284; xxx. 237; preparation of the ammoniac, xxviii. 286; on the external use of it in convulsions, xxxi. 148; instance of its success in chorea, xxxiii. 273; its properties as a coalescent and cicatrizer, 465; its effects on the lungs and nervous system, 474.

ARGILLACEOUS EARTH, mode of analysing, vi. 373; how separated from the oxyd of iron, 474.

ARGILLACEOUS IRON ORE, found in America, i. 198.

ARGYLE, the great Duke of, raised cedars of Lebanon from seed, xxvi. 473.

ARGYLESHIRE FENCIBLES, account of the ophthalmia among the, viii. 185.

———, vitrified fort in, xxviii. 240.

ARISTARCHUS on medical controversy, vii. 260, 371.

ARISTOLOCHIA, beneficial in the bites of serpents, iv. 295; recommended in gout, xii. 158; description of the, xiv. 170; caution on its use, 171; A. Clematitis, observations on the properties of the, xxvii. 393; A. Tomentosa, a new species described, xxvi. 485.

ARISTOTLE on the origin of the nerves, iii. 258; his opinion on hydrophobia, ix. 390; ascribes menstruation to lunar influence, xxi. 30.

ARM, observations on the amputation of the, iii. 368; on the operation of performing it at the shoulder joint, xiv. 108; xxxiii. 400, xxxiv. 525; instance of a child born with only one, xv. 250; case of gangrene of the, xxxi. 398.

——— PRESENTATION, on delivery in difficult cases of, vii. 481, 483; remarkable instance of successful operation in, viii. 394.

ARMENIANS, their ready encouragement of vaccination, xii. 451; the syphilis said to have proceeded from them, xiii. 89.

ARMIGER, Mr. his case of dysphagia produced by aneurism of the aorta, xxviii. 151; elected surgeon of the Eastern Dispensary, xxxi. 259.

ARMINGER, Mr. his report on the state of the army in Spain, xxiii. 159, 160.

ARMSTRONG, Charles, on a case of hydrophobia, xx. 323, 520; on the application of caustic in hydrophobia, 323.

———, Dr. John, character of, xv. 19; on the efficacy of ammonia in scrofula, xxviii. 17; on brain fever produced by intoxication, xxx. 77, 388; his case of diseased cervical vertebræ, xxxi. 307; his facts and observations on the puerperal fever, xxxii. 507; on the treatment of nervous diseases, xxxiv. 427.

———, S. L. his cases of vaccine inoculation, xv. 233, 235.

———, William, on the cause of purulent ophthalmia in children, xxii. 364, 368.

ARMY, on the constitution of the medical department of the, xiii. 275; system of arrangement and discipline for the, xiv. 547; defects in the medical management of the, xv. 88; on the fever which appeared in that of Spain, xxiii. 155, 166; report on that of Walcheren, 183, 187; regulations in passing conscripts for the French, 429; Medical Board, dissolution of the, 438; mortality in the American, xxiv. 435; diseases observed in the army employed on the Walcheren expedition, xxv. 1, 12; on the condition of surgeons in the, xxxii. 417.

ARNELL, Dr. his case of disease arising from supposed poison, xxiv. 373.

ARNEMANN, Dr. on amaurosis, i. 24; his system of materia medica, 99; his description of acoustic tubes, iv. 378; vi. 75; account of his instruments for extracting the cataract, x. 65; becomes editor in the foreign department of this Journal, xiv. 192; on the efficacy of sedum acre in epilepsy, 430; on the viola tricolor in venereal ulcers, 431.

ARNICA MONTANA, beneficial in gutta senna, i. 26; and in agues, iv. 85; effects produced by insects on, xxvii. 406; caution in the choice of, 408.

ARNOLD, Dr. Joseph, his observations on typhus, dysentery, and scurvy, on board the Swedish fleet, xxi. 17, 23; on diseases of the troops, xxv. 13.

———, Dr. Thomas, on the causes and prevention of insanity, xvii. 84; table of the species of madness, 85; on his treatment of those disorders, 92.

AROMATIC VINEGAR, on Mr. Henry's claim to the discovery of, ix. 307, 448.

——— WATER, composition of, iii. 93.

AROMATICS, beneficial in gastritis, xvii. 159.

ARRAGONI, Dr. inoculated for the cow-pox, vii. 182.

ARRAGONITE, analysis of that mineral, xxix. 510; proposed to be called in future hydrous carbonate of lime, 511.

ARRAN, geognostic relations of the rocks in the Isle of, xxv. 453; mineralogical observations on the, 454.

ARROW, case of a wound by an, ii. 54.

ARROWHEAD, description of the, xvii. 466.

ARSENIATED hydrogen gas, analysis and properties of, xi. 422.

ARSENIC, beneficial in cancer, i. 309; danger of the external application of, 501; used with cedar ashes in venereal complaints, 504; method of discovering it in sulphur, iii. 269; case of a child poisoned by, iv. 293; use of in cancer, v. 251; vi. 31; vii. 390; taken into the system by the absorbents, 543; composition of the solution of, vi. 34; observations on its application in cancerous affections, 509; on its external effects, x. 575; peculiarity in the bodies of persons poisoned by, xi. 192; not injurious in a metallic state, 287; used internally in rheumatism, 492; preparation of the acid of, 382; quantity that may be given to a horse, xii. 278; benefit of the solution in chronic rheumatism, xiii. 16, 525; quantity of it in tin, xiv. 33; on its alleged efficacy in hydrophobia, 30, 34; xviii. 401; beneficial in agues, xiv. 100; its effects on three cases of eruption, 113, 117; effects of the solution of, in lepra and other diseases, xv. 297; in consumption, 299; in tapeworm, 300; periodical head-ache removed by the solution of, 506; appearances in the body of a person poisoned by, xvi. 95; effects of the solution of, 113; its use in elephantiasis, xvii. 189; benefit of it in psoriasis, 254; aqua tophana composed of, 255; its use in the preparation of indigo for dying, 583; its efficacy in chronic rheumatism, xviii. 250, 277; and in tic doloureux, 251; cases of fungus hæmatodes successfully treated by, 389; instance of its success in eruptions, xix. 207; in rheumatic complaints, 369; in chronic rheumatism, 476; on the internal use of the acid of, xx. 494; useful in a species of porrigo affecting the scalp, xxi. 133; in hydrophobia, 144; in various chronic diseases, 149; the chief ingredient in the Tanjore pill for the bites of serpents, 277, *note*; on the different methods of detecting minute portions of, 421; on the practice of giving it in agues, 441; new method of detecting it, by Mr. Hume, xxii. 87; its use in intermittents, 219; on the general effects of, 337; to be cautiously used in rheumatic complaints, 348; case of death produced by, 427; on the effects of, xxiii. 238; case of a young woman poisoned by, 384; analysis of the contents of the stomach, 385; particular detail of the test for, 448, 450; its inutility in hydrophobia, xxiv. 29; on a case of poison by, 59; an easy test for detecting, 145; an additional test for, 274; medicinal uses of the prepared white, 340; preparation of the solution of, ibid; observations on the preparations of, 403, 405; efficacious in hooping-cough, xxv. 115; periodical spasm cured by the liquor of, 477; its poisonous effects on plants, xxvi. 31; its action on the stomach, 32; proofs of the poison, ibid; its use in ulcers, 365; case of tic doloureux cured by, 494; analogy between the action of putrid matter and the poison of, xxvii. 518; peculiar gas produced by, 519; experiments with, ibid.; case of a young woman poisoned by, xxviii. 63; tests for detecting, 66, 70; analytical account of, 70; composition of the solution of, ibid; vindication of Mr. Hume's claim to the invention of a test for, 109; effects of that poison on animals, 117; its action on an ulcer, 122; appearances of the stomach in poison by, 123; its property in cancer, 141; its efficacy in the bites of serpents, 155, 6; replies of Dr. Roget to Mr. Hume, on his test for detecting, 187, 189, 469; Mr. Hume's farther observations in behalf of his claim, 284; cases of children poisoned by, 345; observations on them, 349; general view of the dispute, xxix. 10; on its medicinal use, 13; case of poison by, 44; specification of tests discovered by Mr. Hume, 46; in what way it acts on the system, 133, 136; process for extracting it from any of the ores, 525; on its application in herpetic affections, xxx. 22; on its introduction into the system by friction, 26; on the use of nitrate of silver in detecting, 237; its benefit in cutaneous complaints, 296; its effects on the stomach, xxxi. 8; on erosions in the stomach from, 178; benefit of, in chorea, 499; on the volatility of the white oxide in water, xxxii. 253; affection of the stomach from, 428; on its use in cancerous complaints, xxxiv. 198; the danger of it from quacks, 199; indiscriminate application of it to tumours, 201; a venereal ulcer said to be cured by, 348; case of a child poisoned by, 441; on the inflammation in the stomach produced by, 443.

ARSENICAL SOLUTION in hooping cough, inquiry concerning, xxv. 115; success of it in that complaint, and tic douloureux, xxvii. 281, 2; on the formulary of its composition, xxviii. 231; xxxiv. 495.

ARSENIOUS ACID, experiments with, xxxii. 249.

ARTEMISIA, or Southern wood, description and virtues of, xix. 355; cauteries produced from, 357; serviceable in hysteria, 358.

ARTERIAL hæmorrhages, application of the tourniquet in, i. 23; blood, on the circulation of the, xviii. 293.

———— SYSTEM, importance of a knowledge of the, ix. 177; remarkable varieties observed in the, xxviii. 440.

ARTERIES, peculiar arrangement in those of slow moving animals, iii. 489; cause of their being empty after death, vi. 379; on wounds of the, ix. 181; on compressing the great ones at the groin, x. 156; on the motion of blood in the, 284; method of securing them in amputation, xiii. 101; divisions of the, xiv. 52; invention of the ligature for the, 103; on the structure of, xv. 464; on the action of, 465; on suppressing hæmorrhage, how divided, 466, 477; on the ligature of them after amputation, xvi. 79; on suppressing hæmorrhage from divided, 87; unusual origin and distribution of the large, xxii. 512; method of securing them, xxiii. 252; on the functions of the, xxiv. 9; of the wrists, on opening the, xxv. 192; observations on the effects produced by the compression of the, 369; on the unusual distribution of the, xxviii. 505; on the difficulty of injecting the, xxx. 92; influence of the nerves on the beating of the, xxxii. 248; the effects of muscular exertion on the pulsation of the, xxxiii. 225; influence of the nerves on the action of the, xxxiv. 54; on the diseases of the, 129; on inflammation of the coats of, 130; on sphacelation of the coats of, 137; morbid appearances in the coats of, 138; preternatural dilation of the, 140; worms found in the, 149; on the cause of the pulsation of the, 163.

ARTERY, peculiarity in the radial, v. 283; aneurism produced by puncture of the humoral, vi. 535; method of tying it, viii. 1; effect of ligature upon the, ix. 88; on the incontraction of an, x. 2; case of wounded brachial, xii. 339; on compressing the humoral, 504; tying and dividing the trunk of the, xiv. 112; on successful case of compressing the femoral, xvi. 14; the obscurator surrounds the sac in crural hernia, 81; on the operation of the ligature in divided, 88; utility of that of the spleen, xvii. 161; the femoral, cured by tying the external iliac, 190; case of wounded ulnar, 437; case of ruptured, 521; on a truss for compressing an, xviii. 375; two methods of securing an, 377; case of wounded ulnar, 433; observations on the same, xix. 504; case of a wounded one, with amputation, xxi. 317, 321; aneurism of the femoral, 424; aneurism of the carotid, xxii. 67, 69; on stopping the blood in the axillary, xxiii. 329, 479; cases of tying the carotid, xxiv. 17; case of obliterated iliac, 524; further account of that case, xxv. 35; appearances on dissection, 40; inguinal aneurism cured by tying the external iliac, xxvii. 419; instrument for compressing the subclavian, 430; case of a wound of the meningeal, 435; instances of its arising in common with the trunk of the epigastric, 441; on the division of the inner coat of an, xxxii. 430; on tying the subclavian, xxxiii. 221; xxxiv. 118; case of aneurism of the gluteal, xxxiii. 508; on the obliteration of the femoral, xxxiv. 116.

ARTHRITIC CONCRETIONS, analysis of, i. 194.

———— MATTER, its effects in producing dolor facci, vi. 164.

ARTHRITIS, efficacy of oil in, v. 390; warm muriatic baths recommended in, 587; vapours of hepatic gas applied in cases of, vi. 92; on the use of naphtha vitriol in the pains of, 378; observations on the cure of, 454; on the benefit of cold application in, ix. 116, 124, 205, 362, 442, 547; x. 377; objections to that practice, xi. 150; vindication of it, 241; cases of, with observations, 346; on apoplexy and, 348; on the use of Peruvian bark in, xii. 142; aristolochia recommended in, 158; benefit of horseradish in, xviii. 273; on the physiognomy of persons afflicted with, xxvii. 39. See *Gout*.

ARTICULAR CAVITIES, new ones formed after dislocation, ii. 482.

ARTICULATIONS, on unnatural, xiv. 475; case of inflexibility of the, xix. 4; relieved by muriate of lime and iron filings, 5, 8; on the formation of foreign bodies in the, 84.

ARUM, or Wake Robin, botanical description of, xvii. 72; its stimulating powers, ibid.; beneficial in rheumatism, 73.

———— CORDIFOLIUM, a plant of Madagascar, account of the heat evolved by the, xx. 499; description of, 500.

ARUNDO, on its flowering, xxvi. 477.

ASAFŒTIDA, recommended in spasms, i. 50; its beneficial effects in pertussis, vii. 506; its excellence as a vermifuge, xvii. 173; natural history of the, xxi. 475; supposed to have been known to the ancients, 478; analysis of, ibid.; its virtues not sufficiently appreciated, 479.

ASARUM EUROPÆUM, or Asarabacca, botanical distinctions of, xv. 268; its sudorific and diuretic virtues, ibid.; an excellent errhine, ibid.

ASCARIDES, benefit of aloes and lime-water in, vii. 257; enormous quantity voided of, xx. 380; in the alimentary canal, description of, xxvii. 512.

ASCITES, on digitalis in, i. 58; v. 34; xii. 116; an extraordinary case of, ii. 469; cases of, by Dr. Harrington, iii. 205; by Mr. Rolfe, v. 406; on dissecting persons who have died in, 580; case of, with the hydrops vaginæ, 412; case of, in a female, ix. 211; table of operations in, 212; case of a successful operation in, x. 498; quantity of water drawn in a case of, xiii. 303; case of, encysted with hydatids, xvi. 79; advantage of tapping at the navel in, xvii. 209; simple method of extracting water in, xx. 262; said to be cured by the vapour-bath, xxiii. 501; success of digitalis in, xxiv. 141; case resembling the, xxv. 164; on the benefit of tobacco in, 332; remarks on a case of, by Mr. Knowles, xxvi. 388; explanation in reply to the remarks, xxvii. 44; operation of tapping through the vagina, in a case of, 434; successful case of, xxxii. 26; case of aneurism followed by, 448. See *Dropsy*.

ASCLEPIAS ASTHMATICA, an East India plant, a substitute for ipecacuanha, xxvi. 30.

———— Syriaca, the property of its flowers to catch flies, xxvii. 388; observations on other species of the same genus, 389, 398.

ASH, virtue of the bark and leaves of the, xi. 435.

——, Dr. account of the Harveian oration by, xxviii. 423.

ASHBOURNE, Derbyshire, influenza at, x. 213.

ASHES, wood, used in preserving flesh, x. 475.

ASHFORD, Dr. his anatomical tables, xxiii. 514; on the influence of air in shut sacs, xxxii. 208.

ASTHROPODIUM PANICULATIUM, a plant of New Holland, described, xxvii. 84.

ASHTON, a lunatic, shocking circumstances attending his execution, xxxii. 469.

ASIA, the cradle of the small-pox, ix. 452; progress of vaccination in, xii. 122, 447.

ASPARAGUS OFFICINALIS, botanical description of the, xiv. 66; substitute used for, 67.

ASPEN TREE, its bark the food of ravens, xiv. 543.

ASPHODEL, botanical description of, xii. 66.

ASPHYXIA, on ascertaining death in, i. 54; new method of preventing, 179; galvanism beneficial in, vii. 245; viii. 256; xiii. 375. AZOTICA, observations on, vii. 511.

ASPIDIUM IRRIGUUM, a new species of corn discovered in Kent, xxv. 93.

ASPIRATORS, description of the, xi. 101.

ASPLENIUM ALTERNIFOLIUM, discovered in the south of Scotland, described, xxv. 372.

ASSALINI, Professor, his observations on the plague, xvii. 506; his visit to England, xxxii. 430; on dividing the inner coat of the artery, 444; on the operation for artificial pupil, xxxiii. 192; description of his surgical instruments, xxxiv. 2; his success in cases of popliteal aneurism, 117.

ASSES frequently die at the foot of Vesuvius, xiii. 109; experiments on the spleen in, xx. 218, 222.

ASSISANDERS, (Smyrnium Olusatarum) botanical description of the, xii. 554; eaten by sailors, 555.

ASSIZES, history of the black, xviii. 89, 90.

ASTHENIA, on nervous diseases from, vii. 246; cases of simple, xiii. 476; definition of disorders coming under that denomination, xviii. 188; on the causes and treatment of, 189; observations on, xix. 324; case of, xxii. 250; on the treatment of, xxx. 181.

ASTHENIC ulcers, cured by caustic, vi. 24; apoplexy, symptoms and causes of the, xxvii. 155.

ASTHENOLOGY, observations on, v. 377.

ASTHMA, case of one who died of spasmodic, i. 331; successful case of, ii. 140; Dr. Unzer's remarks on the cure of, 169, 291; advice to the aged afflicted with, iii. 507; on the diagnosis of, iv. 261; success of digitalis in, iv. 330; use of vegetable alkali in, v. 561; use of oil in spasmodic, vi. 70; Millar's case resembling the, 94; history of periodical return of, viii. 401; distinction between croup and acute, ix. 90; success of lichen islandicus in humid, x. 379; on digitalis in, xii. 115; marsh trefoil, beneficial in, 126; solanum dulcamara efficacious in, 135; an expectorant for, xiv. 65; benefit of the roots of wake-robin in, xvii. 73; efficacy of the common germander in, 467; white horehound good in humoral, 564; scurvy-grass beneficial in pituitous, xviii. 271; proceeds from various diseases, 476; efficacy of iron in, 477; acids or absorbents necessary in, ibid.; general treatment of, 478, 9; on the spasmodic, xix. 325; exciting cause of the humid, xx. 342; description of a particular species of, 554; observations on, xxi. 94; an hereditary disease, 284; cured by vegetable diet and distilled water, xxii. 83; relieved by spider's web, 375; produced by ipecacuanha, xxiv. 61, 233; on the predisposing cause of, 357; on the use of tobacco in, xxv. 230; on bleeding in, xxvi. 154; the smoking of stramonium said to give relief in, xxv. 66, 377; xxvi. 23, 49, 51, 57, 160, 490; xxvii. 436, 524; xxviii. 71; virtues of the asclepias asthmatica in, xxvi. 30; the white poppy a substitute for stramonium in, xxxi. 170; on the frequency of it in hot climates, xxxiii. 366; case of dyspnœa mistaken for, xxxiv. 175; warm temperature necessary in, 384.

ASTRAGALUS SINICUS, description of the, xxv. 188.

——— TRAGACANTHUS, pure mucilage in the, i. 75.

ASTREA, frigate, treatment of diseases on board the, i. 91, 135, 236, 340.

ASTRINGENT PRINCIPLE in plants, on the, i. 157; how ascertained, 268; manner of separating it, v. 362.

ASTRINGENTS, in diarrhœa, the most successful, xxxii. 379.

ASTROMETER for ascertaining the rising and setting of the stars, described, xvii. 582.

ASTRONOMY, account of Mr. Cavendish's papers on, xxix. 407.

ASTRUC, correction of a mistake by him respecting the origin of accoucheurs, xxviii. 101.

ATHENS, vaccination introduced at, xii. 449; meteorological observations at, xxxiv. 171.

ATHLETIC EXERCISES, observations on training practitioners in, xiii. 532.

ATKINS, George, account of his improved hydrometer, xx. 481.

ATKINSON, James, his case of cancer in the cheek, vi. 448; observations on his case, 509; on a case of apoplexy, vii. 151; reply to, 277; viii. 65; his answer to Dr. Mossman, 363; his explanation, 441; on the use of opium in fever, viii. 50; on metallic casts of the ear, x. 359; on the severe dysentery, xi. 508; xii. 17; his case of monstrosity in twins, xii. 13; on the use of the seton after operations on the eyes, xvi. 208; his case of hernia, xvii. 227, 229; remarkable case of tumour in the neck, xxx. 353.

———, Thomas, his case of puerperal peritonitis, xxxiii. 447.

ATLAS, remarkable case of dislocation of the, xxx. 341.

ATMOSPHERE, on the composition of the, i. 27; process for purifying the, 46; its action on vegetables, 161; composition of, 167; on the proportion of oxygen in, at Martinique, ii. 387; of marshes, experiments and observations on, vii. 113; means of purifying it from contagious matters, viii. 529; full of animalcules, x. 139; variety in the temperature of the, xi. 478; its connexion with diseases, xiii. 105; proper state of the, 106; its influence in epidemics, 210; pollution of that of London, xiv. 184; on the pressure of the, 213; its action on vegetables, ibid.; observations on that of marshes as the cause of ague, xxvii. 103, 447; its effects on the pulmonary system, 292; observations on some peculiar phenomena of the, 485; mutual action of the earth and the, ibid.; on the infectious, xviii. 86; on changes in the, xix. 371; influence of a moist, 475, 545; on the medical importance of considering the state of the, xx. 3; general view of the natural history of the, 365; influence of extraneous matters in the, 366; on contagion produced in the, 368; on examining the state of the, xxi. 91; tables of the highest and lowest temperatures of the, 435; xxiii. 256, 257; improved state of knowledge with respect to the, xxii. 15; want of a natural history of the, 16; on the changes produced in it by respiration, 48; on the origin and constitution of the, 177, 180; on variations in the, xxv. 53; table exhibiting the influence of the moon on the, 85, 86, 150; on the utility of observing the changes of the, 144; on the warm and cold temperature of the, 149; on the periodical alterations of the, xxvii. 16; its effects on germination, 17; on the syphilitic, 138; on the state of the, 264; on the different strata of the, xxviii. 419; quantity of dew produced by a heated, xxxiii. 233; effect of the sun-beams on, 236; its variations injurious to consumptive patients, 341; on that of hospitals, xxxiv. 394, 467.

AUBERN, Dr. his zeal for vaccination, x. 474; xii. 451.

AUBERT, Dr. on the English medical character, v. 502, *note*; his report on vaccination, xiv. 400.

AUDITORY ORGANS, method of applying galvanism to the, ix. 133, x. 61.

AUGITE, on the situation of the, xxvi. 305.

AUGENIUS, Horatius, a medical writer of the sixteenth century, notice of, xxvi. 409.

AUGURAPORY, the title of an alchemical MS. ascribed to Zoroaster, xiv. 91.

AUGUSTIN, Dr. on the medical use of galvanism, vii. 242.

AUGUSTUS, his singular treatment in rheumatism, i. 386.

AUGSBURGH, peculiar mode of brewing at, xx i. 516.

AULD'S, Dr. case of acute bilious fever treated by nitric acid, xix. 106, 109.

AURELIAN on bronchotomy, x. 7; his opinion on the malus cardiacus, xv. 372; on hydrophobia, 411, *note*.

AURICLE, aneurism with perforation of the, xxxii. 389.

AURIE, M.V. his chemical experiments on the saccharine matter of Indian corn, xx. 482.

AUROCHS, discovery of an animal of the tribe of, xxiv. 67.

AURUM PARADOXUM, analysis of the, ix. 473.

AUSONIUS, his supposed acquaintance with the internal use of mercury, vii. 404.

AUSTIN, Mr. account of his apparatus for condensing gases, xxix. 507.

AUSTRIA, progress of vaccination in, viii. 192.

AUSTRIAN GOVERNMENT, prize questions proposed by the, xxiii. 352.

AUTHORS, on the injustice committed by them in new editions of their works, xxi. 412.

AUTUMN, observations on the diseases of the, iv. 490; on the prevalence of typhus in, vii. 54; remarks on fevers in, 320; fever in Ireland, described, viii. 213; observations on, ix. 213; in America, history of the complaints in, x. 365; treatment of, 378.

AVA ROOT, on the intoxicating and injurious effects of the, xxix. 108.

AVENS ROOT, its efficacy in ague, i. 187; xvi. 867, 557; used in brewing, xxvi. 345.

AVERNO, on the poisonous quality of the lake of, xiii. 109.

AVEYRON, account of the savage of, viii. 377.

AVOCETTA RECURVIROSTRA, found on the sea-shore but rarely, xxv. 286.

AXBRIDGE, Mr. on inflammation of the abdominal viscera, xxxii. 89.

AXILLA, on compressing the great arteries of the, x. 156; case of aneurism of the, xxxiv. 118; that of the groin, on tying the artery at the, xxiii. 329, 479.

AYRE, Dr. on the use of venesection in diabetes mellitus, xxxii. 128.

AYRER, Dr. on aneurisms, iv. 367.

AZALEA INDICA, one thousand pounds said to have been offered for the, xxviii. 341.

AZOTANE, a new detonating compound, discovery of, xxx. 424.

AZOTE, its relation to other bodies, i. 45; the acidification of, 171; on the preparation of oxydated, iii. 91; analysis of, iv. 185; experiments on the gaseous oxyde of, xvii. 51, 55; compounded of an unknown inflammable and oxygen gas, xxx. 84.

AZOTIC GAS, phosphorus not luminous in, i. 299; on the action of phosphorus in, vii. 374.

### B

BABAD, Dr. on bleeding in intermittents, xxxii. 345.

BABINGTON, Dr. appointed physician in ordinary to Guy's Hospital, vii. 384; case of exposure to the vapour of burning charcoal, xxii. 154; case of hydrophobia, 157; on a change produced in olive oil by passing through the human body, xxxi. 45; case of fat forming in the intestines, 46.

BACHE, Mr. his case of asthma treated with alkalies, ii. 140.

BACK, a remedy for pains in the lower parts of the, iii. 410.

BACON, remarks on his art of prolonging life, ii. 496; on empirics, xiv. 440; on the science of medicine, xx. 41; obligations of philosophy to, xxi. 2; axioms of, xxvi. 504; his elegant comparison on natural philosophy, xxvii. 2; on the composition of the human body, xxix. 1.

BACOT, J. his account of the effect of belladonna in a nervous affection, xxiv. 383.

BADDELY, Mr. on the influenza in Shropshire, x. 214.

BADGER, John, his singular case of eruptive disease, viii. 106; observations on that case, 200; account of a case of tinea capitis, 554.

BADHAM, Dr. his observations on the pneumonic diseases of the poor, xiv. 83; on inflammatory affections of the mucous membrane of the bronchiæ, xx. 78.

BAETA, Dr. his comparative view of medical theories, v. 388, 577.

BAGDAD, progress of vaccination at, ix. 451; xii. 123; xxxi. 520.

BAGLIVI on the diseases of Rome, xvii. 122.

BAILDON, Dr. his account of a Chinese remedy for pains in the breast, xviii. 176; on the action of digitalis, xx. 14.

BAILEY, H. W. his case of abscess of the brain, xxiii. 376; case of re-union of the middle finger, xxxiv. 248.

————, John, on the use of digitalis, iii. 127; his case of exfoliation, v. 497; his case of uterine hæmorrhage, xxiii. 493; on the eau medicinale, xxv. 114.

BAILLIE, Dr. his farewell lecture, i. 303; on the structure of schirrhus, vii. 85; on a case of disease of the bowels, 104; on the cause of apoplexy, 142; on the causes of tympanites, 235; explanatory plates to his morbid anatomy, viii. 564; his communication on a case of ecchymosis of the labia, xi. 42; on tumours of the brain, xiv. 81; on cancer, 468; on cancerous hydatids, xv. 395; on digitalis in consumption, xvi. 233; remark on his use of the phrase "morbid anatomy," xxi. 89; his case of hydrocephalus, xxxi. 68, 69; on a strong pulsation of the aorta in the epigastric region, 159; his donation for commemorating John Hunter, 344; his observations on a case of aneurism of the aorta, xxxiii. 265; remarks on his opinions concerning the brain, xxxiv. 309; his speech at the Medical Society, 523.

————, Hamilton, his case of suppression of urine, xxvi. 161.

BAILY, Mr. on the eclipse of the sun, predicted by Thales, xxv. 453.

BAINES, Mr. on the influenza at Thirsk, x. 396.

BAIRD, Dr. on diseases of seamen, xxiv. 14; singular case of carditis by, xxv. 396; his case of gun shot wound, xxvii. 265.

BAKER, Sir George, his opinion of cardamum as an antispasmodic, xviii. 273.

————, Mr. (of Uxbridge,) fatal case of, xii. 502; observations on the case, xiii. 144, 274; remarks on the case, by Dr. Kinglake, 360; xiv. 223.

BAKEWELL, Mr. his lecture on the theory of the earth, xxviii. 75; on stratified rocks, 157.

————, Robert, on the composition of artificial soda water, xxxiv. 292.

BALARUC, analysis of the water of, vi. 186.

BALDINGER, Professor, sale of his library, xiv. 192.

BALFOUR, Dr. Francis, on the effects of sol. lunar influence in fevers, xxvii. 398; confirmation of his opinion, xxviii. 169.

————, W. his case of rupture of the lungs in parturition, xxvi. 161; on the re-union of separated parts of the body, xxxii. 410; his observations on the pathology and cure of rheumatism, xxxiv. 63; observations in confirmation of his opinion, 196.

BALL, remarkable case of the extraction of one, iii. 353; description of that which killed Lord Nelson, xv. 72; on accidents ascribed to the wind of a, xxvii. 323; xxviii. 142; on the cause of death from the wind of a, xxix. 63; observations on the different hypotheses, to account for its fatal effects, xxx. 246.

BALLARD, John, on a particular species of ulcer, iv. 408.

BALL-FORCEPS, description of an improved, ix. 2.

BALLHORN, Dr. his clinical observations on consumptive persons, xv. 30.

BALLIN, Mr. his history of an extraordinary abstinence, xv. 510.

BALLINGHALL, George, his case of hydrophobia, xxxiii. 226.

BALLS, new instrument for extracting, xv. 455; on the action of them in gun-shot wounds, xxx. 379.

BALM OF GILEAD, description of the, i. 33.

BALMIS, Dr. his voyage round the world to circulate vaccine inoculation, xvii. 12; xviii. 37.

BALSAM OF JUDEA, account of the, i. 35.

——— OF SULPHUR, composition of, xiii. 502.

BALTIMORE, in North America, medical college founded at, xx. 385.

BAMBERG, on the treatment of diseases in the hospital at, ix. 268.

BAMPFIELD, R. W. his account of diseases on board the Belliqueux, xxviii. 269; on bleeding in hydrophobia, 458; on night blindness, xxxiii. 63.

BANCROFT, Dr. on the yellow fever, xxvii. 36.

BANDAGE for a dislocated patella, iv. 286; observations on the plaster, ix. 113; virtue of, in diarrhœa and dysentery, x. 281; description of an improved one for ruptures, xi. 304; composition of Scultet's, xix. 82; observations on the plaster, xx. 255; on the paper, 256; used by Galen, xxxii. 301.

BANKS, Sir Joseph, on vaccination, v. 108; on a disease in Spanish sheep, xii. 458; his zeal for medical reform, xvi. 347; on the discoveries of Mr. Knight, xvii. 196; on the introduction of exotic plants, xviii. 386; letter to, on abuses in medicine, xix. 462; on naturalizing foreign plants, xx. 20; account of the journal kept by, xxv. 409; on materialism, xxxi. 249; on gout medicines, xxxii. 164; on the poisonous effects of meadow saffron, 216; his case of gout, 495; a single hoofed boar in the possession of, xxxiii. 445.

BARAT, Stephen, president of the physicians at Paris, his liberal conduct, ii. 158.

BARBADOES, success of vaccination at, x. 424; observations on the elephantiasis of, xv. 561; xx. 375; on the climate and diseases of, xx. 240; remarkable disorder in, xxiii. 168; affected by the volcanic eruption in St. Vincent's, xxviii. 162; on the structure of, 239; extraordinary phenomenon at, 251.

BARBARY, observations on the coast of, x. 381; on a peculiar kind of plague in, xxii. 193.

BARBERRY, botanical description of, xiv. 69.

BARBERS, legal disputes between them and the surgeons in France, ii. 158; formerly united in England with the surgeons, xxix. 307.

BARCLAY, Dr. on a peculiar structure of the membrane of the urethra, xiv. 469; on ophthalmia, xviii. 15; his improvement of anatomical language, xix. 483, 488; on the caudal vertebræ of the sea snake, xxi. 262; on the muscular motions, xxii. 5.

BARDSLEY, Dr. on the epidemic catarrhal fever at Manchester, ix. 522; his account of the influenza, x. 210; his case of poison from a vegetable fungus, xii. 384, 512; on cases of supposed rabies canina, 387; case of hydrophobia, with remarks, xiii. 155; his medical reports of cases and experiments, xviii. 275; his account of hydrophobia, xx. 9; on the effects of salivation, xxx. 41.

BAREGES, its situation and waters recommended to consumptive patients, xi. 284.

BARILLA, proved not to contain iodine, xxxi. 346.

BARK, substitutes for it in intermittents, i. 187; given in epilepsy, 223; benefit of the Angustura in ulcers, 502; preference given to the yellow Peruvian, ii. 6; its use in ague, 238; on its use after parturition, iii. 442; experiments and observations on the different kinds of, v. 33; efficacious in obstinate ulcers, 217; beneficial in tertian intermittents, 227; new preparation of the Peruvian, vii. 473; to be cautiously used in scarlet fever, ix. 160; its use in the autumnal fever, 217; its inefficacy in scarlatina, 249; injurious in catarrhal fever, 379; the cinchona recommended in puerperal fever, x. 12; general observations on, 13; experiments on the broad-leaved willow, 374; on its use in diseases of the warm climates, xi. 204; the febrifuge principle of, 215; experiments and observations to shew that it contains gelatine, 266; virtues of the willow, 443, 444; analysis of the, 564; virtues of the Angustura, 566; efficacy of the Peruvian in gout, xii. 142; pleurisy treated with the extract of, 156; virtues of the elm, 230; observations on the willow, 372; superiority of it to the cinchona, 373; on the febrifuge substance in, 430; virtues of that of the Barbary, xiv. 69; efficacy of the cinchona in rheumatism, 273; in yellow fever, 531; utility of that of the Mezereon, 544; of the service tree, 572; medicinal virtue of the oak, xv. 69; benefit of the Peruvian in scarlatina, 493; root of water avens, a substitute for the, xvi. 557; on a poisonous species of Angustura, xvii. 428; glue substituted for the cinchona, xviii. 34; on its use in measles, xix. 144; and in hooping-cough, 145; its use in diabetes, 181; on the effects of the cinchona in gout and rheumatism, 427; on the sophistication of, xx. 21; chemical account of the Angustura, xxii. 144; serviceable in warm climates, 231; its use in ophthalmia, xxiv. 222; on alterations in the names of, xxv. 438; account of the trees which furnish the Peruvian, xxvi. 12; general view of the several kinds of, 325; analysis of, 331; medical properties and uses of, 333; on treating gout with the, 382; influence of cinchonin on, xxvii. 295; observations on various kinds of, 301; improper in typhus, xxx. 322.

BARKER, Dr. on the febrifuge virtue of lime and magnesia, i. 274.

———, John, on the grease in Arabian horses, xvi. 320, 322.

BARLETTA, a Neapolitan surgeon of the 16th century, account of, ii. 279.

BARLOW, Dr. on carbonate of iron in cancer, xvii. 225; his case of dysphagia supervening an inflammation of the lungs, xix. 248; on the origin of calculi, xxi. 148.

———, John, on friction with opium, ii. 107.

———, James, on the delivery of the placenta, iv. 320; on premature labour, v. 40; on the Cæsarean operation, 46; observations on his cases, 253; on shoulder presentation in laborious parturition, viii. 213; on a case of premature delivery, ix. 403; his account of the influenza, x. 209; on the operation for the stone, xi. 1; mode of curing the tinea capitis, xiv. 496; his reply to Mr. Goodlad, xxiv. 162; his case of small-pox subsequent to vaccination, xxv. 390; observations in reply to, 490; address of the editors to, xxvi. 88; his case of lithotomy, xxviii. 496; correction respecting him, by Dr. Merriman, xxix. 289; his case of morbid tumour of the nose, 296.

BARNES, J. his case of inoculated cow-pox, xiv. 351.

BARNSIDE, Dr. his account of the influenza at Sligo, x. 528.

BARNSTAPLE, Devon, influenza at, x. 195; description of a new fossil found at, xvi. 191.

BAROMETER, observations on the uses and construction of the, xxv. 52, 149; observations on Skiddaw with the, xxvii. 170.

BARRACUDA, on the poison of the, xx. 463.

BARROW, John, on the operations of Mr. Adams at Greenwich Hospital, xxxi. 382.

BARRY, Dr. John, on vaccine inoculation, iii. 503; on the hot bath in debility, iv. 146; his letter to Dr. Pearson on the cow-pox, 425; remarks on his opinion, v. 23; on a case of vaccination, vi. 423.

BARTHE, Professor, on a wound in the abdomen and thorax, x. 250.

BARTHOLOMEW'S HOSPITAL, resolution of the medical society of, i. 312; lectures at the medical theatre of, xiii. 95; xiv. 285; xv. 94; xvi. 285; xx. 288; xxii. 351; remarkable case of elephantiasis in, xxv. 84.

BARTISCH, on the disorders of the eye, ii. 368.

BARTLETT, Michael, his case of hydrocephalus internus, xi. 401; on opium in intermittent fever, xii. 3; on the virtues of nicotiana in hernia, 401; and in colica pictonum, 403; his observations on marks in children, xv. 249; case of a child born with one arm, 250.

———, Dr. on the history of medicine in America, xxvi. 215.

BARTLEY, O. W. his case of incontinence of urine, ii. 288; on the evil of quackery, iii. 433; iv. 230; case of extracting the placenta, 232; on artificial musk, 234; on the hooping-cough, v. 137; on the digitalis in phthisis, 259; on the influenza, x. 308; on delivering the placenta, xi. 456; on a case of imperforate anus, xix. 420.

———, Mr. on the baneful effects of sulphuric acid upon children, xxxiv. 436.

BARTON, Dr. on the effects of datura stramonium in mania and epilepsy, viii. 426; on the Spigilia Marilandica, 428; on vermifuge medicines, 429; on the bite of serpents, xi. 334; on the torpidity of animals, xxiii. 502; xxiv. 187; observations on his opinion, 188; on the fascinating power of the snake, 393; his description of the opossum, xxvi. 422; on certain vegetable muslicapæ, xxvii. 388; his Flora Virginica, xxviii. 517; xxix. 10; botanical works of, xxx. 347; his description of a singular bird, xxxiv. 169.

BARTONIA DECAPETALA, a new genus of plants described, xxviii. 342.

BARYTES, application of the carbonate of, i. 301; muriate of it recommended in spasmodic asthma, ii. 291; preparation of the muriate of, 297; on the use of the muriate of, in ulcers, 469; method of exhibiting the, iii. 170; new method of preparing the muriated, 581; recommended in diseases of the eyes, 585; its effects on the circulation of the blood, v. 303; analysis of, vi. 374; on preparing the muriated without kali, 467; another method of preparing, xi. 382; solution of the muriate of, in muriatic acid, xii. 61; the acetite of a test for vinegar, xxv. 85; experiments with, xxviii. 123; use of the carbonate of, in consumption, xxx. 486; analysis of, xxxiii. 34; remarks on Mr. Hume's paper on, 35.

BARZELOTTI, Professor, his observations on muscular contraction, xxi. 457.

BASALTES, chemical analysis of the, ix. 475.

BASILIC POWDER, composition of the, xv. 432.

BASSE, M. his method of preparing muriatic ether, x. 94.

BASSORA, in Persia, vaccination introduced there, xii. 123.

BATAVIA, prize question of the economical society of, i. 296; on the endemic of, xi. 201; on the prevalence of gravelly complaints at the city of, xvi. 164.

BATCHELDER, Dr. his case of disease of the heart, xxxii. 307.

BATEMAN, Dr. his case of encysted tumour in the brain, xiv. 80; on the larvæ of two insects discharged from the human body, xxv. 447; on the progress of vaccination, xxvi. 389; on the poison of mercury, xxx. 342; his synopsis of cutaneous diseases, 412; on his description of elephantiasis, xxxiii. 107; history of tubercular eruption, 410.

BATES's TINCTURE, composition of, xxvi. 191.

BATH, cold, advantages of the, iv. 399, 402; beneficial in rheumatism, ix. 549; its use in puerperal fever doubted, x. 18; recommended in gout, xii. 159; historical account of the practice of, xiii. 565; its use after fever, xix. 109, 111; a preventive of fevers, xxii. 230; xxiii. 118; regulations concerning the, xxvi. 397.

BATH, vapour, description and use of the, vii. 289; viii. 54; benefits produced by the, ix. 175; facts and observations on the, 484; observations on the, xiii. 522; description of Mr. Cochrane's, xxiv. 34; its use illustrated in a case of hydrocephalus, xxv. 317; queries respecting the, xxvii. 95; account of a medicated, xxviii. 242.

——, the warm, advantages of, in laborious parturition, i. 288; recommended in nervous fever, ii. 466; efficacious in nervous debility, iv. 146; serviceable in gout, v. 587; its use in puerperal fever questioned, x. 17; recommended in bilious cases, xi. 519; to be used in burns, xxi. 387; beneficial in fever, xxiii. 118; on the general use of xxvi. 398; its benefit in tetanus, xxix. 101.

——, utility of the shower, xxxiii. 273.

——, convenient method of constructing a steam, xxiv. 162.

BATH (City of), curious experiments in the hospital iii. 278; cases in the dispensary at, vi. 195, 388, 491; vii. 14, 189, 402, 553; viii. 15, 207, 208, 364; influenza at, x. 105, 226; of the Jennerian society formed at, xiii. 187; xiv. 348; report of the same, xv. 309; table of patients in the hospital at, 566; cautions by the physicians and surgeons at, xxi. 526.

—— WATERS, observations on the medicinal powers of the, iv. 457; x. 183; on the virtue of, xii. 405; their use in diseases of the hip-joint, xv. 565; quantity of iron in the, xvi. 383; chemical experiments on the, 435.

BATHAM, Dr. on swallowing cherry-stones, xxv. 475.

BATHING, on the management of it in fevers, xiv. 87; good effects of it, 89; useful in agues, 101; directions for the practice of it, 179; utility of it in yellow fever, xv. 18; in summer, conducive to health, xvi. 281; in hydrophobia, observations on, xvii. 185, 223; xviii. 400; in the Thames, on, xx. 91; on the machines for, ibid; general observations on, xxvi. 396; on the use and abuse of, xxx. 224.

BATHS, account of remarkable ones used in Russia, xiii. 76; frequented by the ancients for luxury and health, 464; institution of vapour, xxi. 176; proposal for sea-water ones in the metropolis, xxiv. 359; on establishing public, xxvii. 342; proceedings of an institution for that purpose, 423; on the utility of, xxviii. 26; description of warm ones at Paris, xxx. 223; account of some established at Bristol, xxxi. 61.

BATS, various species of in the East Indies, xxx. 429; enormous ones called flying foxes, xxxiii. 339.

BATTERY, description of Mr. Davy's grand galvanic, xx. 254; improvements in the, xxi. 527; account of Mr. Children's large galvanic, xxx. 426; xxxiv. 165.

BATTLE, on certain accidents that occur in, xxvii. 323.

BATTLEY, Richard, on an improved method of making vegetable extracts, xxxiv. 293.

BATTY, Dr. resigns the editorship of the Journal, xx. 387.

BAUDELOCQUE, M. on the Cæsarian operation, i. 391; on the amputation of the uterus, 392.

BAUER, Ferdinand, his botanical researches in New Holland, xxx. 524.

BAUHINUS, Gaspar, his invention of anatomical terms, ii. 68; his anatomical discoveries, iii. 75; acquainted with the ligamentum secretum, 258.

BAUME, M. on calcareous muriate of lime, i. 194.

BAXTER, Richard, his case of excision of the uterus, xxv. 210.

BAYEN, Pierre, memoirs of him and his works, i. 196.

BAYLE, Dr. on the white induration of organs, xvi. 470.

BAYLEY, Dr. on a scirrhous pancreas as the cause of the jaundice, vi. 218.

———, Mr. of Manchester, his philanthropy, ix. 307.

———, Rowland, on inflexibility of the articulations, xix. 4.

BAYNTON, Thomas, his method of treating old ulcers, ii. 92, 197; success of his adhesive plasters, v. 217; benefits of his bandage, ix. 115; his plaster applied in tinea capitis, xii. 9; good effects of his adhesive straps, xx. 110; method of treating diseases of the spine, xxxi. 245; advantages of his adhesive method in ulcers, xxxiii. 466.

BAYREUTH, analysis of the black chalk of, i. 506.

BAYSILLANCE, Dr. his case of tinea, x. 542.

BEAUCHAMP, M. on a disease of the heart, i. 298.

BEAUMONT, Mr. his account of the influenza at Melbourne, x. 202.

BEATTY, Mr. his account of the death of Lord Nelson, xv. 72.

BEAUTY, method of the Turkish ladies for improving their, i. 35.

BEAVER, Dr. his case of cynanche trachealis, xxvi. 377.

BEAUVOIS, M. his description of a new genus of insects, viii. 282.

BECCHER, John Joachim, account of his chemical theory, i. 63.

BECKMANN, Professor, his method of staining wood, i. 201; on the virtues of ergot in uterine hæmorrhage, xxxii. 97.

BECLARD, M. his instances of monstrosity, xxxii. 434; on the breathing of the fœtus, 521.

BECQUET, M. on the vitiation of the iris, xxii. 468; efficacy of belladonna in, 478.

BED, made of oat-bran, recommended for infants, ii. 394; directions for constructing one to convey sick and wounded persons, xx. 142.

BEDDOES, Dr. on nitrous acid in venereal complaints, i. 100; correction respecting Dr. Scherer, 142; on digitalis in phthisis, 292, 384; on pulmonary consumption, 410; his reply to Dr. Crichton, ii. 200; on the advantage of living with cows, 415; on the pneumatic institution, 488; his opinions controverted, iii. 274; on the use of the acids and the pneumatic institution, 486; prospectus of lectures on animal nature, 487; his communications on the anti-venereal power of acids, v. 71; reply to, by Mr. Blair, 501; his queries on the influence of parents on their progeny, vi. 24; his plan for trying the acids, 165; on the delay of his popular essays, 362; his great exertions, vii. 8; on the calx muriata as a remedy for scrofula, 58; on vaccination, viii. 7; case of phthisis cured by digitalis, ix. 264; gratitude expressed to, 266; on the influenza, x. 47, 73, 103, 193, 289, 302, 385, 517; on a medical institution for the poor, 571; his report of the preventive medical institution, xii. 479; on the solution of arsenic in consumption, xv. 299; his letter to Dr. Jenner, xvi. 43; on fever, as connected with inflammation, xix. 168; his letter to Sir Joseph Banks on the abuses of medicine, 462; cases of hydrophobia, xx. 195, 198; account of cases and dissections, 407, 428, 540; his death, xxi. 86; vindicated, 89; character of, 90; note from him, 91; censured with respect to his advice to medical students, 161; account of his posthumous papers, 176; memoirs of, 183; proposed a medical school in London, xxiv. 500; correction in the memoirs of, xxv. 91; memoirs of his life and writings, 172, 259, 343; on lessening the number of physicians, 314; his visionary notions, xxvi. 391.

BEDFORD, on the death of the Duke of, vii. 279.

BEECH, botanical description of the, xvi. 74.
BEEF, experiments on the preservation of, xxxii. 436; on that used in the navy, xxiii. 235.
BEER, on the antiseptic property of, iii. 90; improper in gout, viii. 355; improved by the herb Bennet, xxvi. 345; on the fermentation of, xxxiii. 91.
——, Dr. on cold water in amaurosis, iv. 179; his case of hydatids in the kidneys, v. 303; his queries on ophthalmia, xviii. 493; on epidemical ophthalmia, xxiv. 317.
BEES, their sting produces erysipelas, iv. 301; buck-wheat recommended for, xv. 67; poisonous effects of sumach on, xvii. 487; their attachment to thyme, 566; the attachment of an idiot to, xxvii. 313.
BEET ROOT, method of extracting sugar from, ii. 188; varieties in the, 189; on Achard's experiments with, iii. 8; a substitute for coffee, 271; experiments to obtain sugar from the, iv. 249, 250; method of obtaining sugar and brandy from the, 284; experiments on the sugar of the, 366; cheap method of obtaining sugar from, xvii. 197; the yellow, a substitute for coffee, xxii. 525; loaf of sugar from the red, xxv. 363.
BEGONIA, beautiful species of the, xxviii. 164.
BELCOMBE, Dr. on acute rheumatism, xxxi. 269.
BELFAST, influenza at, x. 301.
BELGUERE, surgeon to Frederick the Great, an enemy to amputation, iii. 37.
BELL, Mr. of Pocklington, his account of the influenza, x. 517.
———, Benjamin, his observations on tympanites, vii. 234; examination of his opinions on the venereal poison, ix. 571; his operation for the crural hernia, xi. 120; his instructions for operating at the shoulder-joint, xiv. 109; his opinions on stricture controverted, xxi. 373.
———, Charles, his system of dissections, i. 404; review of the same, v. 373, 579; vi. 80; his anatomy of the brain, viii. 473; his proposal for a museum at Edinburgh, xiii. 191; character of his work on the anatomy of expression, xviii. 8; objection to his wire bougies, xxi. 313; engravings from his anatomical collection, xxviii. 241; on the muscles of the ureters, xxx. 170; elected surgeon of Middlesex Hospital, xxxi. 433.
———, John, on turpentine in burns and scalds, iii. 206, 296; his observations on scrofula, iv. 57; case of elephantiasis, 58.
BELL, John, (of Edinburgh) his principles of surgery, ix. 84, 177, 276; on compressing the arteries at the groin, 501; observations on his principles of surgery, x. 154; his case of scald, xiv. 399; on his discourse of wounds, xix. 565; on his treatise of tumours, xx. 242; his letters on professional character and education, xxiv. 343; on professional prejudices, xxvii. 325.
BELLADONNA, its utility in gutta serena, i. 26; its benefit in mania, ii. 72; its use in dolor facei, 466; its effect previous to the operation on the cataract, iii. 367; observations on the medical uses of the, iv. 5; successful in hydrophobia, vi. 93; its use in the extraction of cataract, 352; a remedy for scarlet fever, 368; botanical description of the, xii. 133; its leaves beneficial in cancer, 134; its influence in inflammation of the iris, xvi. 55; its effects on the iris, xxii. 478; experiments with the, xxiii. 455; formula of preparing, xxiv. 340; singular effect of it in an uncommon nervous affection, 383; produces delirium in animals, xxvi. 252; efficacious in hooping-cough, 510; analysis of the, xxviii. 205; insoluble in alcohol, 208; its influence in cataract, 258; efficacious in dilating the pupil of the eye, xxx. 511; new analysis of, xxxi. 519; one hundred and eighty persons poisoned by, xxxii. 390; efficacious in tic douloureux, 433.
BELLAMY, G. on catarrhal fever, iv. 290; his case of fracture of the cranium, v. 255; on a morbific elongation of the tongue, vi. 353, 435, 522; case of diseased bladder, xvi. 141; xviii. 511; observations on that case, xvi. 251; strictures on it, 253; xvii. 34; his answer, 39; farther remarks on the case, 258, 527; his case of calculus in the urethra, xviii. 319; his account of a lusus naturæ, ibid.; reply to his account of diseased bladder, xix. 13; his case of lacerated urethra, xxi. 209; observations on that case, 367, 487.
BELLAMY'S, Dr. observations on the hooping-cough, viii. 41; on the virtues of the Zanthoxylon, 453.
BELLINGHAM, R. on hepatic and pneumonic inflammation, xxx. 190; on the efficacy of bleeding, 191; on a case of hydrophobia, xxxiii. 12; observations on that case, 178.
BELLIQUEUX, diseases on board the, xxviii. 269.
BELLOT, M. on hydrophobia, ii. 468; case of ascites, 469.
BELLOT, Abraham, his case of incarcerated femoral hernia, xviii. 45; case of suppression of urine, xix. 227; observations on his case of hernia, 529; defence of his practice, xx. 259; reply to, 508; case of strictured intestine, xxii. 392.
BELLS, effects produced by the ringing of, viii. 403.
BELLY-ACHE, on the dry, xiii. 486.
BENAMOR, Dr. the last practiser of magnetism; his death, xxxii. 257.
BENEVOLENCE, on the external organ of, xxxiii. 501.
BENGAL, progress of vaccination in, xiii. 257, 336, 569; xx. 23; on the unhealthiness of, xxi. 75; influence of the moon on the climate of, xxvii. 401.
BENNET, the herb useful in brewing, xxvi. 345.
———, H. G. his account of the Island of Teneriffe, xxviii. 236.
———, Rev. Mr. his experiments on electricity, vii. 478.
BENNETT'S, Mr. extraordinary case of abscess, vi. 294.
BENNION, Thomas, his account of the fever at Gibraltar, xiv. 136.
BENON, M. on venereal diseases, ii. 469.
BENTHAM, General, his method of preserving water sweet in long voyages, vii. 477.
BENZOIC ACID, analysis of the, i. 160; on the separation of the, v. 366; in the urine of horses, viii. 476; not a constant principle of urine, xix. 130; on the crystallization of, xxvii. 128.
BERENGAR DE CARPI, his anatomical discoveries, ii. 279, 281; first observes the cœcum, iii. 74; examined the uropoetic vessels, 76; on the brain, 257; on the origin of the nerves, 259, 260.
BERGEN, in Norway, vaccination introduced there, xvi. 478.
BERGER, Dr. his case of necrosis of the tibia, xiv. 300; reflections on the case, 303.
BERGIUS on the application of opium to the skin, vii. 123.
BERGMAN, Professor, on ascertaining the purity of cotton, ii. 83.
BERKELEY, Earl, his letter to Dr. Jenner on the cow-pox, vi. 105; his evidence in favour of vaccination, viii. 20.
BERKSHIRE, resolutions of medical gentlemen in, xxxiii. 337.
BERLIN, prize question of the Medical College at, xiv. 381; state of the university of, xxvii. 385.
BERLIN DISPENSATORY, account of the, iii. 93; transactions of the society of natural history at, 284.
BERNARD, Charles, his case of leucorrhœa, xv. 425.
———, John, his case of compression of the brain, xxxii. 8.
———, Mr. description of his instrument for the cure of ovarian dropsy, viii. 387.
———, Sir Thomas, on the fever institution, xii. 163.
BERNE, in Switzerland, hospitals at, xxv. 407.
BERNOUILLY, M. on preparing acetate of potash, xxv. 518.
BEROE FULGENS, a luminous sea animal, described, xxv. 414.
BERRY, Mr. his case of cure of the bite of a snake, xxvii. 432.
———, Dr. observations on cases of hydrophobia, xxviii. 353.
BERTHOLLET, M. his discovery of a new acid, i. 85; his observations on eudiometry, vi. 376; his experiments on muriatic acid, 475; on the fulminating mercury, viii. 190; on the comparative effects of heat and light, ix. 85; on the effects of air and water in meat, xxv. 252; on chemical phenomena, xxix. 515; process for making calomel, 363.
BERTIN, M. his method of raising water, xvii. 197.
BERTRAND, M. on the use of hepar sulphuris in tettery eruptions, xxxii. 165; on the virtues of charcoal, 248; on vaccination and hooping-cough, 256.
BERYL, new earth found in the Siberian, iii. 587; iv. 82; another in the Saxon, ibid.; analysis of the, vi. 473; chemical analysis of the, ix. 475; analysis of the new earth discovered in the, xi. 285.
BERZELIUS, Professor, on the alcohol of sulphur, xxix. 509; xxx. 79, 80, 81; on the composition of animal fluids, xxx. 9; his analysis of bile, 12; on sulphuret of carbon, 81; on the composition of azote, 85; general view of the animal fluids, 233; on the serum of the blood; xxxii. 222; on the elements of the blood, xxxiii. 353.
BESANCON, prize question of the Medical Society at, xxv. 456.
BESNARD, M. composition of his antisyphilitic tincture, xxviii. 473.
BEST, Henry, his case of uterine rupture, xiii. 234; his thesis on vaccination, xiv. 408.
BESTUCHEFF, method of preparing the nervous tincture of, vi. 474.
BETHLEM HOSPITAL, observations on a view of, xxxii. 124.
BETHUNE, J. on the use of mustard cataplasm, xiii. 553.
BETONY, its virtues as an errhine, xvii. 563; peculiar property of the, xxvi. 31.
BEVAN, E. his case of vaccine inoculation, v. 455.
BEVERIDGE, Mr. his case of uterine cancer, viii. 431.

BEZOARDS, on the number and characteristics of, ix. 498.
BHANG, an article smoked in Ceylon, description of, xxix. 106.
BIANCHI, on artificial impregnation, i. 47; on hepatic affections, xxiii. 231.
BIBB, W. W. on the modus operandi of medicines, viii. 90.
BIBLIOGRAPHIA MEDICINÆ BRITANNICÆ, prospectus of a, xvii. 395.
BICHAT, Xavier, his physiological researches on life and death, v. 476, 563; on the cause of arteries being empty after death, vi. 371; on the membranes, 449; on the application of general anatomy to physiology and medicine, vii. 472; his death and character, viii. 382; on the opinions of, xxii. 258, 333; his experiments on the brain, xxxii. 57.
BIDEFORD, Devonshire, account of the influenza at, x. 103.
BIDLAKE, Rev. Mr. the influenza in his school at Plymouth, x. 144.
BIENVENU, M. on the theory of electricity, xvi. 190.
BIERKEN, M. his case of enlarged tongue, xxxii. 169.
BIGGIN, Mr. his experiments on bark, v. 37.
BILE, on a substance precipitated from the, i. 330; the incentive and stimulus of many functions, viii. 239; the deficiency of it in yellow fever, ix. 30; on complaints of the, xv. 194; physiological observations on the, xvi. 199; quality of the hepatic, 200; on the cystic, 202; on the changes and uses of the, 297; supposed to be a secretion from human blood, xviii. 426; on determining the capacity of, xxi. 69; putrefies slowly, xxii. 184; on vitiated, xxiii. 514; on its use in promoting digestion, xxiv. 247; an excretory fluid, xxvii. 280; remarks on its use in the system, xxviii. 98; analysis of the, xxx. 12; extravasation of it into the cavity of the abdomen, xxxii. 152.
BILIARY CALCULI, ether and turpentine good against, i. 394; treated with vitriolic ether, 499; observations on, xv. 3.
BILIARY MATTER, account of a peculiar, xxx. 10.
BILIOUS COLIC, efficacy of alkalis in the, viii. 293; warm bath recommended in, xi. 519.
——— DISEASES, treatment of the, viii. 241; observations on, xxiii. 513.
——— EXPECTORATION, case of hepatitis followed by, xiv. 469.
——— FEVER, causes and cure of, i. 105; on the cause of remitting, 246; at Birmingham, described, iii. 94; in North America, 568; on the prevention of, vi. 310; observations on, ix. 372; case of acute, xix. 106; in the Mediterranean, xxxi. 489.
——— FLUXES, virtue of barberries in, xiv. 69.
——— VOMITING, saline mixture with absorbents recommended in, xvi. 381.
BILL, Mr. on the treatment of tinea capitis, xiii. 8.
BIND WEED, botanical description of, xii. 129.
BINNS, Dr. his observation on vision, xiv. 152; on the scarlatina anginosa, xv. 489; xxvii. 470; xxxii. 484.
BIONDO, Michael Angelo, an Italian surgeon, account of, ii. 279.
BIOT, M. forms water from hydrogen and oxygen, by compression, xiii. 575.
BIRCH, on obtaining sugar from the black and white, iv. 248; botanical description and uses of the, xii. 34; description of the dwarf, 36.
———, John, his evidence on Dr. Jenner's petition, viii. 142; on the medical application of electricity, ix. 286; his opposition to vaccination, xiii. 468; on alleged failures of the cow-pox, xiv. 376; answer to, xv. 304.
BIRDS, on the digestive power of nocturnal, ii. 79; researches concerning the voice of, vi. 279; on the odours exhaled by, 440; on the iris in, xvii. 412; on the migration of, xxiii. 502; xxv. 88; discovery of the claws and feathers of an enormous one, xxvii. 79; on the structure of the eye in, 257; question concerning migratory, 380; description of an organ in the eyes of, xxix. 481.
BIRD'S-CHERRY, antimonial virtue of the bark of, xvi. 76.
BIRKBECK, Dr. on oil of turpentine in tænia, xxv. 168; use of a new solution of ferrum tartarisatum, xxviii. 244.
BIRMINGHAM, on the bilious fever at, iii. 94.
BIRTH, account of a monstrous, xviii. 345.
BIRTHS, proportion of deaths in child-bed to, xxv. 213.
BIRTHWORT, description of a climbing, xiv. 170.
BISCHOFF, Dr. on the medical application of galvanism, vii. 529.
BISHOP, T. case of suspended animation, vi. 116; on advice respecting leeches, xv. 198.
BISHOPP, T. his case of optical illusion, xiv. 117; case of rheumatism producing phlegm on, 118; remarks on the preceding case, 323; case of fracture of the sternum, xviii. 415.

BISHOP'S CASTLE, influenza at, x. 105.

BISMUTH, its efficacy in spasmodic affections, i. 50, 503; and in spasms of thes tomach, xv. 563; recommended in cramp, xviii. 281.

BISSET, Dr. on the virtue of hellebore as a vermifuge, xvii. 465.

BISTER, observations on it as found in the distillation of wood, xxviii. 235.

BISTOIRE CACHE, description of an improved one, by Mr. Carlisle, iii. 193; another, by Mr. Watt, 195, 297; on its advantages, xi. 14; its superiority to the gorget, xxviii. 498.

BISTORT, botanical description and uses of the, xv. 66.

BITCH, the artificial impregnation of a, i. 47.

BITTER EXTRACTS, method of preserving, iii. 581; a useful one obtained from the leaves and roots of the tulip, iv. 376; on the vegetable principle of, xxxiv. 165.

BITUMENS, chemical analysis of the elastic, ix. 474; analogy of various substances with, xxviii. 235; discovery of a new one, xxxii. 81.

BITUMINOUS WOOD, acid found in, vii. 381.

BIZET, J. B. on the motion of the sap in plants, xiv. 209.

BLACK, description of a woman whose skin was partly, xxix. 508.

———, case of a man whose skin became, xxxiii. 73.

———, oak-apples give out a fine, xv. 70.

——— ASSIZES, at Exeter, Oxford, and London, accounts of the, xviii. 89; xx. 370.

——— CATARACT, on the existence of, xxxiii. 310.

——— MATTER in the glands of the lungs, observations on, xxix. 506.

——— VOMIT, account of the, xiii. 378; xvii. 124.

——— BRONCHIAL GLANDS, on the colouring matter of, xxxi. 128.

———, Dr. Joseph, account of his chemical discoveries, i. 169; his discoveries known to the ancients, vi. 409; account of a posthumous treatise of, viii. 383; recommended carbonic acid in calculous complaints, xxiii. 477; on his theory of animal heat, xxix. 150; life of, xxxiv. 36.

———, Dr. (of Newry,) account of the influenza, x. 295; his case of angina pectoris, xv. 380.

———, John, his translation of Humboldt's Essays on New Spain, xxv. 239.

BLACKATON, Devonshire, extraordinary mortality at, xxviii. 23; xxix. 14.

BLACKBERRY, effectual in gravel, xvi. 263.

BLACKBURNE, Dr. his theory of light, iii. 501; his improved method of administering spermacetti, 515; on the cure of the scarlet fever, x. 457.

BLACKLEY, Dr. his remedy for dropsy, xiii. 288.

BLACKMORE, Sir Richard, opposed inoculation for the small-pox, xvii. 236.

BLACKSMITHS, on the deafness peculiar to, vii. 423.

BLADDER, case of extraction of the stone from the, i. 120; on the diseased and contracted urinary, ii. 303; account of a mortification of the, iii. 456; ossification in the, 587; case of diseased, with an enlarged prostate gland, iv. 390; observations on diseases of the, v. 285; on injuries done to the, vi. 1; analysis of stones found in, 528; new mode of extracting stones from the, vii. 429; derangement of it relieved by the vapour-bath, x. 61; alleged cure for, 82; on wounds in the, xi. 3; operation of cutting the, 6; on diseases of the, 61; hemlock, a remedy in an inveterate disease of the, 342; on the puncture of the urinary, xiii. 10; child contained in the urinary, xiv. 519; case of laceration of the, xv. 153; case of leucorrhœa mistaken for ulceration of the, 425; case of diseased, with fistula, xvi. 141; xviii. 511; observations on the case, xvi. 251, 253; on the use of the gall, 201; remarks on Dr. Bellamy's case, by Mr. Thomas, xvii. 34; answer of Dr. Bellamy to his opponents, 39; practical observations on diseases of the, 386; case of an overcharged, xviii. 570; Mr. Dawson, in reply, on a case of diseased, xix. 13; state of it in a case of ischuria, xx. 118; on detecting madder in the, 215; case of obstruction in the neck of the, 225; calculi found in that of a dog, xxi. 369; objection to the introducing of the caustic bougie into the, 377; on the swimming one of fishes, xxiii. 453; on the air bladder, 454; calculus taken after death from the, xxiv. 185; case of diseased, 463; case of rupture of the gall, xxv. 185; case of suppression of urine, from inflammation of the neck of the, xxvi. 161; on the muscular coat of the, xxvii. 381; case of worms in the, xxviii. 154; on discovering the stone in the, 498; advice on passing the catheter into the, xxix. 234; on the retention of urine in the, 271; on venæsection in inflammatory affections of the, 271, 454; effects of the muscles of the ureters in the irritation of the, xxx. 170; case of diseased prostate, xxxi. 225; observations on diseases of the, 241; on catarrh of the, 243; case of abdominal inflammation in the, 371; retention of the urine treated by puncturing the, xxxii. 43; varicose veins in the, 155; on fungous excrescencies of the, 156; cancer of the, 157; on worms in the, 159; fatal case of an organic affection in the, 169; cure for incontinence of urine in, 521.

BLAGDEN, Sir Charles, on short-sightedness, xxix. 426.

———, Mr. his case of ecchymosis of the tibia, xi. 42.

BLAGDON, Mr. case of abscess of the liver with gall-stones, xxxi. 157.

BLAINE, Mr. on the cure of hydrophobia, xx. 10.

———, Mr. on the symptoms of rabies in dogs, xxxi. 231.

BLAIR, Thomas, his report of the Sussex Jennerian Institution, xiv. 347.

———, William, his essays on the venereal disease, i. 409; on the origin of the venereal disease, ii. 352; on living with cows as a remedy for consumption, iii. 15; his essays on the venereal disease, 190, 275; on a supposed case of cancer, iv. 569; explanatory letter on that case, v. 192; his reply to Dr. Beddoes, 501; on the operation of œsophagotomy, vi. 97; on hydatids in cancer, 193; on charitable institutions for the sick poor, 363; on still-born children, vii. 284; on the management of ruptures, x. 375; his case of an extraneous substance in the vagina, 491; enquiry into the origin of the venereal disease, xiii. 89; his case of small-pox after vaccination, xiv. 21; his anatomical articles in the new Cyclopædia, 110; on Rowley's pretended ox-faced boy, xv. 445; on vaccine inoculation, 529; account of the vaccine contest, xvi. 475; his case of obstruction in the œsophagus, xvii. 20; his hints to Parliament on vaccination, xix. 556; his letter to Mr. Birch, 559; his animadversions on the reviewer of his book, xx. 60; his queries to Mr. Davies, xxi. 438; answer to, 478; his oration on the origin of syphilis, xxv. 364.

BLAKE, Dr. on the structure and formation of the teeth, viii. 558; on the influenza at Taunton, x. 116.

———, Andrew, his description of an improved tourniquet, xii. 336; on the hooping-cough, xxvii. 102.

BLANC, experiments and phenomena observed on Mount, xxxiii. 248.

BLAND, Dr. on retained placenta, iii. 464.

BLANE, Sir Gilbert, on the benefit of nitrous fumigation, iii. 94; letter of Dr. Johnstone to, on a case of voracious appetite, 209; fatal case of hæmorrhage of the liver, iv. 65; communication on the plague, v. 538; on the Egyptian blindness, vi. 357; his evidence in favour of the Jennerian discovery, vii. 489; recommends floating lazarettoes, xiii. 273; on the nature and properties of yellow fever, xviii. 468; xx. 31; his pension doubled, xx. 474; on muscular contraction, xxi. 459; his observations on the wind of a ball, xxvii. 324; on the Walcheren fever, xxx. 15; facts and observations respecting intermittent fevers, by, 159; on the prevalence, mortality, and treatment of different diseases, xxxi. 501; on a case of cynanche laryngea, xxxii. 151; on infection, xxxiv. 245.

BLAQUIERE, Edward, on the diseases and practice at Tripoli, xxxi. 478; on the sirocco, xxxiv. 356.

BLEACHING, improved liquor for, ii. 187; an improved apparatus for, 287; with oxygenated muriatic acid alone, 297.

BLEAR-EYE, on the treatment of the, xxv. 85.

BLEEDING, on stopping it by the tourniquet, i. 23; improper in puerperal fever, 388; recommended for apoplectic children, 392; case of a person who lost his life by improper, with remarks on that case, ii. 29; recommended in spasmodic asthma, 169; in hydrophobia, 174; useful in hot climates, 178; ill effects of, in putrid and bilious fever, 179; cautions respecting the use of, 233; observations on a case of improper, 265; Dr. Vaughan in explanation of that case, 405; recommended in hydrocephalus, 327; ill consequences from unskilful, 346; efficacious in gout, 378; by leeches, remarks on, iii. 39; improper in croup, 56; general indiscretion in, 59; improper in injuries of the head, 60; in hydrocephalus, 119; observations on, 226; defended, 240, 328; beneficial in preventing puerperal convulsion, 451; objections to it in fever, 538; in phlegmatia dolens, v. 94; in hydrocephalus, how to be used, 128; improper in cases of simple fracture, vi. 401; on its use in measles, vii. 303; proper in apoplexy, 333; not difficult to stop in amputation of the thigh, viii. 3; recommended in phthisis, 85; improper in erysipelas, ix. 15; on its use in yellow fever, 37; said to be necessary in apoplexy, 321; sometimes exhilarating, 337; vindicated in apoplexy, 413; in catarrh, 422; beneficial in yellow fever, 581; injurious in puerperal fever, x. 13; not to be employed in hot climates, 58; in American epidemics, xi. 312; dangerous in apoplexy, 349; in fever, observations on, xiii. 277; powerful in the cure of acute diseases, 473; its use in the early stage of cynanche trachealis, 555; in dysentery, 562; on staunching it in amputation, xiv. 105; success of it in hydrophobia, 122; danger of it in general cases, 183; vindication of the practice of, 306; improper in scarlatina maligna, 371; its danger in apoplexy, 379; advantageous in remittent fever, xv. 191; case of ulceration following, 348; in diseases of the abdominal viscera, 482; beneficial in inflammation of the bowels, 543; necessary in inflammation of the iris, xvi. 57; of the nose, on stopping the, 191; on the various uses of, 198; in pulmonic inflammation, 482; its use in ophthalmia, xvii. 385, 480; good effects of it in the cure of an hæmorrhage of the lungs, xviii. 330; its efficacy in a wound of the lungs, 333; in rigidity of the os externum, 337; in difficult parturition, 339; in hydrophobia, 402; to be cautiously used in measles, xix. 141; fever induced by the bites of venomous insects, cured by, 220, 222; remarkable disposition to, in some families, xx. 69; recommended in pains of childbearing, 74; in pneumonia, 92; in rheumatism, 249; on the use and abuse of, 321; on its importance in acute organic affections, 586; on its utility in purpura, xxi. 146; success of it in difficult parturition, 179; highly necessary in measles, 365; its benefits in fevers, xxii. 228, 459; influence of cold at the scrotum in restraining that of the nose, 327; of the urethra, from the application of a bougie, 341; extraordinary instance of, xxiii. 175; in fever of the brain, 517; efficacy of it in palsy, xxiv. 375; in hernia, 513; on the danger of using improper lancets in, xxv. 31; cases shewing its utility in scarlatina, 102, 106, 407; caution recommended in that complaint, 298; in fever, xxvi. 108; effectual in hæmorrhœa petechialis, 153; indispensible in puerperal fever, 270; family subject to fatal, 422; diabetes treated by, 497; on the advantages of copious, xxvii. 89, 189, 285, 472; criterion for determining its propriety, 103; how far proper in puerperal fever, 183; in croup, 208; case of diabetes treated by, 332; puerperal convulsions cured by, 345; on the side affected, or on the opposite, an agitated question at Leyden, 488; on its utility in imperfect crises, slow convalescence, and lingering ailments, xxviii. 131; relieves inflammation of the trunk, 376; on its use in hydrophobia, 352, 438; xxix. 12; successful in typhus, xxviii. 476; remarkable history of, 492; efficacy in hydrophobia, xxix. 12, 23; observations on the practice of, 35; recommended by Boerhaave, 37; further remarks on the practice, 42, 105; on its efficacy in retention of the urine, 271; case of hydrophobia cured by, 285; on its propriety in inflammatory affections of the bladder, 454; a cure in tropical fever, xxx. 76; in gastritis and hydrophobia, 97; in hepatic and pulmonic inflammation, 191; advantage of it in tropical climates, 229; used with effect in fever in the Mediterranean, 247; successful in croup, xxxi. 81; objections to it in hydrophobia, 122; on Mr. Borrett's proposal of it, 281; fatal effects of, 471; the sovereign remedy in remittents, 489; injurious in croup, xxxii. 54; its use in diabetes mellitus, 128; unsuccessful in hydrophobia, 219; for obstinate chancres recommended, 232; bad effects of it in poison from opium, 271; recommended in intermittents, 345; account of an hereditary disposition to, xxxiii. 9; unsuccessful in hydrophobia, 12; xxxiv. 426; arguments in favour of, xxxiii. 178; caution in the use of it, 226; its efficacy in aneurism, 375; on periodical, xxxiv. 110; advantageous in fever, 249.

BLEGBOROUGH, Henry, on chronic croup, xv. 507.

—————, Ralph, his description of the vapour-bath, vii. 289; viii. 54; on cold water in typhus, 158; on the nature and cure of gout, 349; case of paralysis, ix. 175; facts and observations on the vapour-bath, 484; on the benefit of steam in gout, xii. 62; observations on his paper, 216; on animal heat in typhus, 244; reply to, 341; his vindication on gout against Dr. Kinglake, 375; letters to, 378; on a posthumous work of Dr. Garnett, 379.

BLENHEIM-STREET, statutes of the Medical Society of, xxvi. 504.

BLENNORHŒA, on the inoculation of, xii. 519.

BLENNORHAGY, observations on the causes of the venereal, ix. 573.

BLIND PERSONS, on the methods of writing and calculating of, viii. 572.

BLINDNESS, corrosive sublimate useful in a case of, iv. 184; produced by an effusion of blood, v. 68; cured by electricity, 70; on the Egyptian, vi. 357; viii. 555; cases of obstinate, viii. 548; case of the cure of, ix. 198; progress of a case of, xiv. 15; causes of it in sheep, xxvii. 219; method of treating it in the East Indies, xxx. 221; case of periodical day, 509; on night, xxxiii. 63.—See *Cataract* and *Ophthalmia*.

BLISS, John, his observations on the Kilburn water, vi. 240; defence of his analysis, 332; reply to, 513.

BLISTERING FLY, description of one newly discovered, xxiv. 430.

BLISTERS, successfully applied in chorea, i. 22; in croup, 148; in dysentery, 465; in spasmodic asthma, ii. 291; beneficial in hydrocephalus, 328; iii. 119; in phlegmasia dolens, v. 94; in suppression of urine, vi. 526; in delirium, 571; new formula for, vii. 81; recommended in diseases of the knee, viii. 471; ix. 374; in puerperal fever, viii. 513; x. 17; in the influenza, viii. 526; serviceable in yellow fever, ix. 102; in scarlet fever, 160; in apoplexy, 412; serviceable in the diseases of hot climates, x. 39; used in the American epidemics, xi. 314; recommended in cutaneous diseases, xiv. 371; on the opening of those caused by burns, xviii. 410; useful in checking mortification, 443; in glandular swellings, xix. 363; in pneumonia, xx. 245; in inflammatory cases, xxi. 83; in hydrocephalus internus, xxiv. 212; easy mode of applying, xxvii. 452.

BLIZARD, Thomas, his case of axillary aneurism, xxxiv. 118.

————, Sir William, his case of trismus, xxviii. 155; his Hunterian oration, xxxiii. 244.

BLOCH, Dr. his account of an uncommon worm, xxviii. 413.

BLOOD, its circulation promoted by electricity, i. 55; potass a constituent part of, 300; state of oxygen in the, 389; method of attenuating viscous, 503; history of the discovery of its circulation, ii. 282, 368, 459; on that of a fœtus, 371; the pabulum of life, iii. 60; on its circulation in particular animals, 376; on the oxygenation of the, 547; iron particles in the, iv. 186; blindness produced by an effusion of, v. 68; effect of muriated barytes on the, 303; quantity of carbon in the, vi. 192; on the discovery of the circulation of the, 411; examination of the colouring part of the, 469; its impregnation with oxygen, 496; on the vitality of the, 512; apoplexy caused by its extravasation on the brain, vii. 77, 142, 335, 454, 518; on the circulation of it in the fœtus, viii. 95; on the coagulation of the, 279; on the colour of venous, 501; on the oxydation of the, 502; on its changes in the lungs, 504; quantity of it taken into the lungs by respiration, ix. 75; effects of galvanism on, 291; its fibrine sensible to galvanic irritation, ibid.; on an issue of the arterial, x. 4; analogy between the motion of water and that of the, 284; on carbon in the, xi. 47; changes produced by respiration on the, 85; experiments on that of an ox, 287; effects of animal poisons on the, 484; on the discovery of the circulation of the, xiv. 74; peculiar discharge of in females, xv. 186; chyle found in the, xvi. 199; observations on venous, xvii. 93, 162; description of the, xviii. 576; physiological view of the circulation of the, xix. 191; effects of digitalis in excessive action of the, 420, 422; on the action of the air and the, 547; experiments on its circulation, xx. 175; on the gelatine of the, xxii. 74; xxiv. 10; case of vomiting of, xxii. 183; cases of extravasation of it on the brain, xxiv. 89, 95; the serum of it like milk, xxv. 150; observations on the serum of, 151; cause of the red colour of the, 281; observations on white serum in the, 300; coagulated lymph in serum of the, 341; on its circulation in parts inflamed, xxvi. 354; sugar in that of diabetes, 462; on the constituent parts of the, xxvii. 73, 168; comparison of different fluids with the serum of the, xxviii. 63; experiments on the serum of the, 67; on its quantity in animals, 323; chemical researches on the, 386; analysis of the serum of the, 391; experiments on the coagulation of, 393; on the colouring matter of, 394; effects of acids on, 395; effects of alkalies on the, 397; method of obtaining artificial, 470; chemical observations on, xxx. 9; on the temperature of the, xxxi. 340; on its circulation in the fœtus, xxxii. 168; new analysis of the, 222; on the extrication of caloric during the coagulation of the, 223; on its subsidence in inflammation, 270; on the action of certain substances injected into the, 343; on the capacity for heat in the, xxxiii. 114; on the comparative temperatures of venous and arterial, 116; case of exudation of the, 246; on different states of the, 276; on the vitality of the, 279; elements of the, 353; on Mr. Hunter's doctrine concerning the, xxxiv. 29; effects produced by extravasated, 49.—*Spitting of*, see *Hæmoptysis*.

BLOOD-ROOT, or SANGUINARIA CANADENSIS, virtues of the, xxv. 166; good in pulmonary complaints, 167.

BLOOD VESSELS, experiments with injections into the, iii. 587; method of injecting the, v. 375; on the compression of the large, x. 154; diseases connected with, 466; observations on the crural, xi. 54; action of opium on the, 146; action of the mind on the, xxii. 464; on injecting different gases into the, xxvi. 252; on compressing them in diseases, xxviii. 368; on the art of injecting, xxx. 89; xxxi. 459.

BLOOMSBURY DISPENSARY, vaccine inoculation introduced in the, viii. 566.

BLOSSOMS, how to preserve them from frosts, i. 297.

BLOUNT, J. on vaccine inoculation, vi. 114.

———, Dr. on the influenza at Hereford, x. 126.

———, J. S. on a section of the symphysis pubis, xxiii. 498.

———, J. S. his cases of gutta serena and cancerous affection, xxviii. 28; inflammation of the spinal marrow, 29.

BLOWPIPE, a new portable one, by Dr. Wollaston, described, xvii. 99; another, by Melograni, 196.

BLUE, substance in iron, analysed, viii. 479; oak-bark and copperas make a durable colour, xv. 70; on the preparation of Prussian, xvi. 105; a fine one from the centaurea, xix. 539.

BLUE COLOUR of the skin, on the, xxxiii. 50.

——— FEVER, description of the, v. 586.

——— BOY, remarkable descriptions of, vi. 498; xv. 381.

——— GIRL, case of a, xiv. 471.

——— DISEASE, history of a case of, x. 575.

——— POCKS, observations on those said to have appeared after vaccination, xiii. 48.

BLUHM, Dr. of Riga, restores Kotzebue to health, vii. 94.

BLUMENBACH, M. on the capacity of negroes, i. 404; his account of the platypus, iv. 81; his anatomical remarks on the Ornithorhynchus Paradoxus, vi. 73; his observations on the iris, xvii. 404; on the cataract, xxviii. 259; on vegetables found in some species of minerals, xxix. 430; his institutions of physiology, xxxiii. 279.

BLUNT, Mr. on epilepsy, xxxiii. 428.

———, Charles, his meteorological observations, xxxi. 175, 263, 351, 439, 527; xxxii. 87, 175, 263, 351, 439, 527; xxxiii. 79, 164, 255, 343, 430, 518; xxxiv. 86, 174, 262, 350, 439, 527.

BLYTH, S. C. on the use of enemata, iii. 229; on the practice of Hippocrates, iv. 151.

BOARDING SCHOOLS, advice to the keepers of, x. 458.

BOAT, description of a life, iii. 286; account of another invented on new principles, xxi. 173.

BOBBA, Dr. on the cause of rickets, vi. 191.

BOCKER, Dr. his account of a pseudo syphilitic disease in Norway, xxii. 432.

BODIES, on the odours exhaled by human, i. 243; on the combustion of inflammable, 304; importance of opening those of the dead, iii. 17; on the effluvia of odoriferous, 89; on the essential parts of compound, ibid.; influence of chemistry on animal, vii. 261, 460; on opening dead, ix. 73; on the chemical knowledge of mineral, 472; apparatus for removing extraneous ones, x. 479; human ones converted to mummies, xi. 192; means of preserving them from putrefaction, 358; xvi. 95; peculiarities in animal, xviii. 36; on the effluvia of dead, xxiv. 422.

BODKIN, one extracted from the bladder of a woman, xi. 17.

BODSON, M. his case of inflammatory catarrhal head-ach, xxxiii. 426.

BODY, on odours exhaled from the human, i. 243; converted into fat, iii. 10; influence of the moon on the human, viii. 401; on the economy and structure of the, ix. 476; effects of metals on the, x. 575; on the combustion of the human, xii. 87; on transformations of the organs of the human, xvi. 301, 451, 517; xviii. 36; dissection of a morbid, xix. 160; on the muscular motions of the human, xxii. 5; effects of the solar heat on the, xxiii. 193; relation between the time of the day and various functions of the, xxxiii. 225.

BOECKMANN, Professor, on the illumination of rotten wood, v. 583; on the influence of light on phosphorus, vi. 374; on the action of phosphorus in gas, vii. 374; on the putrefaction of flesh in gas, ix. 219.

BOERHAAVE, on the medical theory of, i. 487; character of, 491; on his doctrine of inflammation, iii. 146; on apoplexy, vii. 153; on chalybeates, viii. 174; his industry, 418; predicted a specific against the small-pox, xi. 325; on the nature of Glauber's spirit of nitre, 410; on the virtue of dog's grass, 530; on purges in gout, xii. 155; relieved by the juice of buck-bean, 128; on the virtues of nightshade, 135; on the efficacy of currants, 225; case of a dread of water in acute fever, xv. 412; on the hydrophobia, xvii. 185; his high opinion of the medicinal property of iron, 474; on the virtues of dandelion, xix. 167; recommended the burdock in pleurisies, 295; his treatment of ulcers, 262; on the fluidity of water, xxix. 18; recommended bleeding in hydrophobia, 37.

BOILING, experiments on the effects of, xxxiv. 42.

BOILS, produced by fixed air, xiii. 36.

BOLETUS, description of two species of, xxix. 522.

BOMBAY, on the dysentery at, i. 349, 352; report of the Medical Board at, on cow-pox, ix. 534; on the introduction of vaccination at, xxix. 156.

BOND, Mr. on the influenza, x. 226; his case of re-inoculation, xiii. 38; on dislocation of the spine, xxxi. 271.

BONES, experiments on burnt, i. 29; phosphoric acid and lime in human, 154, 396; observations on those of Europeans, 406; historical view of discoveries of the, ii. 160; alterations produced by disease in the, 183, 380, 464; effects of gout on the, 378; articular cavities formed in dislocated, 482; separation of those of the metacarpus in paralysis, iii. 253; discovery of enormous ones in America, iv. 186; on the structure of the, 273; on amputating the extremities of the cylindrical, 406; on those of the pelvis previous to delivery, v. 50; preternatural swelling and growth of the, 491; on the mollification of the, vi. 191; on fractures of the cylindrical, 201; observations on the union of, in simple fracture, 397; observations on the marrow of, 470; on diseases arising from various causes in the, vii. 415; references to the, viii. 279; on inflammation of the, 301; fatal effects of bruises of the, 302; on removing dead portions of, 303; on the growth of the, 370; case of caries of the, ix. 179; observations on caries, x. 4; on extracting splinters from the cranium, 370; on amputating the extremities of the, 557; goose-grass tinges them red, xi. 534; case of fracture of the occipital, xii. 15; effects of madder on the, 29; magnesia in animal, 58; method of preparing broth from, 350; case of caries of the os frontis, 393; on the veins of, 551; on dividing them in amputation, xiii. 100; concretions of the nature of, 385; case of rapid growth of the, 393; analysis of one extracted from the orbit of the eye, xiv. 471; caries of the, with syphilitic ulceration, xvi. 78; on their conversion into membranes, 451; on the anchylosis of the, 452; fatal case of decay of the, xvii. 311; on diseases of the, xix. 79; prize question on the fracture of the, xx. 286; on the use of the medulla of, ibid.; solution of continuity in the parietal, xxiii. 352; on the exfoliation of the, 456; on the commercial uses of, xxv. 98; remarkable fossil, xxvii. 24; removal of the metacarpal, 333; fracture of the occipital, xxviii. 63; on caries of the, xxix. 177; on the actual cautery in destroying the caries of spongious, 181; of the spine, analysis of the, xxxi. 498; how affected in venereal complaints, xxxii. 230; small pieces of, discharged from the breast, xxxiii. 8; on magnesia in human, 159; in that of the thigh, 469; on the methods of producing re-union in fractured, 508.

BONE-SET, a remarkable plant from North America described, xxvii. 428.

BONE-SETTERS, empirical and dangerous practices of, xv. 553.

BONONIAN PHOSPHORUS, peculiarities in the, ii. 388.

BONPLAND, account of his travels in South America, xiv. 187.

BOOKER, Dr. his sermon on the cow-pox recommended, viii. 171; his letter on the cow-pox, xi. 433.

BOOKS, observations and cautions on reading medical, xii. 506; disingenuity often practised in new editions of, xxi. 412; on the multiplicity of medical, xxiv. 2; proper ones for medical students, xxix. 253.

BOORDE, Andrew, his remedy for the tooth-ache, ii. 271; anecdotes of him, xxvii. 116.

BOOTH, Miss, secondary small-pox in, xxvi. 83, 236.

BORACIC ACID, carbon the basis of, ii. 248.

BORACITES, new analysis of, ix. 92.

BORAT OF MERCURY, powers and uses of the, viii. 118.

BORAX, on the unknown acidifiable basis of, v. 199; recommended for chapped nipples, xxxi. 258.

BORAGE, experiments on the distilled water of, x. 95; description and virtues of the common, xii. 126.

BORDA, Dr. on the virtues of the lauro-cerasus, xiv. 572.

BORGUIRA, or rattle-snake, description of the, xxiv. 392; fascinating power of the, 393.

BORICHIUS, Professor, died under the operation of cutting for the stone, xi. 19; his account of the cure of cancer, xvii. 208.

BORINGDON, Lord, his bill to prevent inoculation for small-pox, xxx. 85, 171.

BORRETT, G. his case of strangulated hernia, v. 113; his case of amputation of the thigh, ix. 499; on compressing the artery of the groin, x. 155; his case of hydrophobia, xxi. 268; on the analogy between gastritis and hydrophobia, xxix. 451; Dr. Kinglake's explanation respecting, xxx. 96; Dr. Adams on the same subject, 201; on gastritis and hydrophobia, xxxi. 1; on the digestion of the stomach after death, 3; Mr. Abel on his claims, 6, 281.

BOSCH, Dr. on the enlargement of the ovaria, viii. 444; on a remedy for colliquative diarrhœas, x. 477; on manna in the flowers of the pontic dwarf rose-bay, xxii. 261.

BOSQUILLON, Dr. on a case of hydrophobia, iv. 55; his singular hypothesis on hydrophobia, ix. 390; refuted by facts,, xxviii. 250.

BOSSEN, disappearance of the Isle of, xxiii. 263.

BOSTOCK, Dr. his case of epilepsy cured by argentum nitratum, i. 222; account of diseases in the Liverpool Dispensary, v. 117; his observations on urine, ix. 349; on the analysis of animal fluids, xiv. 263; xv. 280; analysis of a stearoid tumour, xv. 276; his two cases of diabetes, 569; his remarks on Mr. Ellis's theory of respiration, xix. 547; reply to, xx. 181; on mineral poison undetected in the stomach, xxi. 148; his evidence in the case of Miss Burns, 340; on the gelatine of the blood, xxii. 74; on animal chemistry, xxiv. 10; on the nomenclature of the New London Pharmacopœia, xxv. 431; his analysis of the cactus coccinillifer, xxvi. 342; on the serum of the blood, xxviii. 67; on diabetes insipidus, xxx. 167; on the bark of the cocoloba uvifera, 168; on the bones of the spine, xxxi. 498; on the urine and serum of blood, xxxiii. 63.

BOSTON, North America, medical school established at, xxvi. 247.

BOSWORTH, Newton, on the accidents of human life, xxix. 492.

BOSWELL, Montgomery, his case of vaccination, v. 341.

BOTALLI, his treatment of gun-shot wounds, ii. 57; pretends to the discovery of the foramen ovale and arterial canal, 372.

BOTANIC GARDEN at New York, account of, xx. 18; xxvi. 88; catalogue of the plants in, xxvi. 162; history of the establishment of, 164; at Berlin, xxvii. 388.

BOTANICAL LOTTERY, account of Dr. Thornton's, xxvi. 345.

———— POCKET-BOOKS, on the arrangement of, ii. 491.

———— REPORTS, Monthly, xxiv. 438, 528; xxv. 92, 187, 370, 442; xxvi. 170, 256, 425, 512; xxvii. 82, 258, 347, 524; xxviii. 163, 340; xxix. 260, 433; xxx. 524.

———— SYRUP, composition of the, i. 158.

BOTANICUS AMERICANUS, account of that work, xxvii. 260.

BOTANY, on the application of chemistry to the study of, i. 72, 157, 263, 377, 495; iii. 370; v. 361, 467; view of the various systems of, i. 204; on the philosophy of, ii. 397; proposal for the improvement of, 482; on the discovery of the sexual system of, vi. 415; anatomy of plants, vii. 557; necessary in medical education, viii. 278; advantages of the study of, xi. 368; on the ardent pursuit of, xiii. 5; ignorance of it disgraceful, xx. 12; researches in, 16; improvements in the medical distribution of, 138; on the practical study of, xxii. 108; on the study of, xxvi. 291; notes relating to the history of, 470; cultivated in France, xxvii. 22; defence of the natural method in, xxviii. 75; that science much attended to by the Royal Society, 318; discoveries in, xxx. 251; system of Welsh, xxxiii. 409; new mode of teaching, xxxiv. 258.—See *Plants.*

———— BAY, on the yellow gum of, vi. 429; vaccination introduced at, xiii. 346; xv. 523; lost there, xxvi. 518; account of the resinous liquid gum of, xxix. 474.

BOTENTUIT, M. on a case of paralysis, iii. 253.

BOTTLE, method of preparing a luminous, xv. 94; method of preparing phosphorous, xxiv. 86; on the caoutchouc, xxxiv. 55.

BOUCHER, Mr. his observations on elm trees, v. 492; account of an organic disease of the heart, xxii. 137.

BOUDET, M. his method of preparing phosphoric ether, viii. 569.

BOUFFEY, Dr. on the influence of the air in diseases, iv. 449.

BOUFFLERS, case of the Duchess of, xiv. 176.

BOUGIES, history of the invention of, i. 58; improvements in the construction of, ii. 85; cautions in the use of caustic, 303; on the benefit of, iii. 289; on the application of, v. 514; on the use of caustic, 573; examination of the metallic, vii. 238; objected to, in removing strictures, ix. 5; benefit of caustic, 190; advice respecting the management of, xi. 477, 558; on the advantages of the flexible metallic, xii. 276; case of stricture removed by caustic, xiv. 474; farther remarks on the use of flexible metallic, xvii. 460; construction of the same, xviii. 243; benefit of tobacco, 350; observations on the caustic, 383; description of a newly invented one, xx. 315; objections to the caustic, xxi. 163; advice in the application of, 313; on the effect of Mr. Home's, 377; recommendation of those made of horn, xxii. 387; case of hæmorrhage of the urethra from the application of one, 341; comparative merit of the caustic and common, 517; on the introduction of the, xxv. 269; useful in procidentia ani, xxvii. 269; construction of sedative soluble, xxxi. 516.

BOUILLON-LAGRANGE, his account of a liquid styrax, i. 163; on camphor and camphoric acid, iii. 89; his method of preparing syrup of quicksilver, vi. 15.

BOULLAM, in Africa, pestilential fever imported from, v. 184; account of, xvii. 128.

BOULLAY, M. his process for preparing sulphuric ether, xxii 174, 362.

BOULOGNE, vaccination introduced at, iv. 470.

BOURBON, state of vaccination in, xxx. 506; ravages of the small-pox in the isle of, xxxiv. 168.

BOURDEAUX, programme of the prize proposed by the Medical Society at, viii. 280.

BOURGEOIS, Louisa, her instructions in midwifery, xxviii. 101.

BOURQUET, M. on a case of paralysis, iii. 253.

BOURNE, Dr. on uva ursi in pulmonary consumption, xiv. 463; his reply to the British Critic, xvii 288; character of his work, xviii. 9; his pathological remarks on a case of abstinence, xx. 527.

BOUTTATZ, Dr. the Fothergillian medal awarded to, v. 314; account of an extraordinary tumour of the eye, vi. 289; his cases of catalepsy and epilepsy, 425; address to, 461; employed to extend vaccination throughout Russia, ix. 584

BOUTFLOWER, J. J. on opiate frictions, vi. 433.

BOUVIER, M. on extracting the oil of juniper, iii. 90.

BOUVION, Dr. on an epidemical disorder in Germany, iii. 77.

BOVEY COAL, oil extracted from, xxviii. 235.

BOWEL, on securing a wounded, xxvii. 64; on recovery from a mortified, 131; on a strangulated, 134.

BOWELS, on disorders in the, i. 208; attended with flatulence and barbarygnis, ibid.; astringents discountenanced in, ibid.; observations on complaints in the, 352; observations on a singular disease in the, vii. 104; on the treatment of inflammation of the, viii. 161; use of alkalis and absorbents in disorders of the, 289; on the causes of diseases in the, 363; how to be treated, 364; on the treatment of inflammation in the, 472; account of a particular affection of the, ix. 183; constipation of them removed by external friction, xv. 325; cases of inflammation of the, 542; frequency of disorders in the, xvi. 283, 312; disorders in them ascribed to the eating of fruit, xviii. 372; xxii. 284; insanity caused by disease in the, xix. 94; on complaints of the, xxii. 348; on the treatment of the, 434; on their state in ulcers, xxix. 200.

BOWEN, Professor, his case of preternatural labour, xxxiii. 514.

————, Mr. his case of vaccine inoculation, xiii. 245.

BOX TREE, botanical description and uses of the, xii. 37.

BOY, one mistaken for a girl, ii. 300; description of a blue, vi. 498; one found in a savage state, viii. 378; remarkable instance of passion in one, ix. 288; fœtus found in the abdomen of a, xxii. 166; xxiv. 7; xxxii. 81; xxxiv. 317; premature puberty in a, xxiv. 7, 389; one with a large head, xxxiv. 44.

BOYER, M. his lectures on the bones, xix. 79; observations on the campaign in Russia, xxxiii. 300.

BOYLE, Alexander, his case of wounded diaphragm, xxvii. 333; on the fevers of Sicily, xxviii. 221.

———— Hon. Robert, his chemical discoveries, vi. 414; on the colour of the centaurea, xix. 539; on the necessity of air for combustion and respiration, xxxii. 321; his ointment for leprosy, 356.

BOYS, Dr. his defence of the medical officers of the navy, xvii. 250; his letter on the practice of midwifery, xix. 178.

BOZZINI, Dr. his invention to inspect the interior of wounds, xvi. 576; xviii. 39; trial of his instrument, 387.

BRACHIAL ARTERY, case of wounded, xii. 339.

BRACONNOT, Henry, his analysis of gamboge, xxv. 320; on the fleshy part of mushrooms, xxix. 520; on the fat in animal bodies, xxxiv. 435.

BRADFORD, Yorkshire, influenza at, x. 387.

BRADLEY, Dr. James, on strangulated hernia, xxiv. 112, 193; his case of venous aneurism, 285; remarks on hæmatocele, with cases, 289; on an epidemic puerperal fever, with cases, xxv. 193; observations on puerperal fever, xxvii. 183.

——————, Dr. Thos. his account of publications and experiments on the cow-pox, i. 1; on yeast in fever, v. 145; his evidence on the Jennerian discovery, vii. 491; letter from Dr. Pearson to, viii. 156; character of his Medical Dictionary, ix. 285; his case of sudden death, xii. 391; his observations on hydrophobia, xx. 543; on worms in the human body, xxix. 334.

——————, Mr. his case of abscess in the groin, v. 219; case of tertian intermittent, 226; case of tenesmus, or locked-jaw, 321; on the reduction of hernia, xvi. 95; his observations on a case of incarcerated hernia, xix. 529; reply to, xx. 259; his defence and farther explanation, 508.

——————, J. A. his case of burn cured by the stimulant plan, xxix. 196.

BRADYPUS URSINUS, account of the, xxv. 99.

BRAGGE, Nicholas, on the vaccine discovery, viii. 154.

BRAIN, observations on monsters born without any, i. 52; on congestions of the, 396; effects of gout on the, ii. 377; case of fatal concussion of the, 407; notions of the old anatomists on the, 460; hernia in that of a fœtus, 480; on the Edinburgh treatment of concussion of the, iii. 33; cases of concussion of, 34; observations on the puriform destruction of the, 70; on the knowledge of the structure of the, 256; on the physiology of the, iv. 90, 334; on the fever of the, 300; electric fluid in the, 335; on epilepsies from the, 380; on the nerves of the, 552; action of the heart on the, 563; its influence on the lungs, 565; on its effects in producing death, 566; injury to the brain by Dalby's Carminative, v. 39; on its state in hydrocephalus, 127; on its slow progress to perfection, 172; on epilepsy as originating in the, vi. 377; case of violent concussion of the, 505; case of extravasation of blood upon the, vii. 77, 142; cavities containing a serous fluid in the, 147; apoplexy produced by compression of the, 206; doubts respecting the effusion of blood to the, 335, 363, 454, 518; viii. 78; on the anatomy of the, viii. 467, 473; apparatus for preventing injury to the, 482; case of injury by fracture, ix. 272; how affected in apoplexy, 321; effects of opium on the, xi. 147; case of singular affection of the, from an internal cause, xii. 109; another from an external cause, 196; case of sudden death from a morbid affection of the, 391; appearances on dissection, 392; whether chorea arises from dropsy of the, xiii. 409; case of incysted tumour in the, xiv. 80; case of diseased, 247; case of collapsed state of the, 289; on the bony covering of the, 298; account of Dr. Gall's new doctrine of the, 327; cases of contusion of the, 486; on the bony covering of the, 529; on the internal dropsy of the, xv. 121, 380; state of it in chorea, 127; effects of bronchocele on the, xvi. 208; sympathy between the stomach and, 278; on ossification of the, 452; on tubercles in the, 470; on transformations of the medulla of the, 522; on the anatomy of the, xvii. 539; case resembling a fever of the, xviii. 152; case of fever of the, 153; chorea symptomatic of pressure on the, 223; on changes in the, 224; observations on a demonstration of its state in sudden and violent death, 308; case of wounded, 359; its condition in fever, xix. 173; xx. 35; on Gall and Spurzheim's doctrine respecting the configuration of the, xxi. 149; animal motions derived from action of the, 203; on congestions of the, 299; on compression of the, 323; observations on that of maniacs, &c. 339; its state in insane persons, 504; case of tumour in the, xxii. 161; observations on pressure of the, 165; state of it in epilepsy, 485; case of suppuration of the, xxiii. 89, 192; case of abscess of the, 376; appearances on dissection, 377; on bleeding in inflammatory affections of the, 518; case wherein mercurial excitement was transmitted to the, xxiv. 52; case in which the medulla oblongata bore considerable pressure, 57; examination on dissection, 58; case of extravasation of blood upon the, 89; examination after death, 90; case of blood effused into the substance of the, 92; appearances on dissection, ibid.; another case of extravasation of blood upon the, 95; appearances on dissection, 96; translation of diseased action from the surface of the body to the, 181; observations on it in fatal cases of the Walcheren fever, xxv. 11; effects of stramonium on the, 385, 507; its influence on the action of the heart, xxvi. 58; xxvii. 12; on concussion of the, xxvi. 131; in hydrocephalus, on the, xxvii. 40; dissection of it in maniacs, 513; history of an affection of it from injury, 521; xxviii. 86; anatomical plates of the, xxviii. 12; on a disorder of the functions of the, 119, fatal case of hydatids in the substance of the, 154; account of a hernia of the,

186; on its state in apoplexy, 213; its connexion with the liver, 215; effects of titillation on the, 226; on the lymph in the ventricles of the, 472; case of incysted tumour of the, 502; all fever attributed to the, 505; uncertainty in the knowledge of the, xxix. 49; inquiry concerning concussion of the, 108; influence of it in the generation of animal heat, 141; observations on that paper, 441; case of concussion of the, 460; observations on that of man and other animals, xxx. 55; treated with alcohol, 56; desiccation of the, 57; on the fatty matter of the, 58; examination of that which is insoluble in alcohol, 63; putrefaction of the, 65; of the nerves in the, 67; on a fever of the, produced by intoxication, 77, 338; observations on fever of the, 516; case of diseased testicle, accompanied by disease of the lungs and, 164; method of separating the complicated parts of the, xxxi. 249; comparison between that of the human and of the brute species, 253; beneficial effects of mercury in affections of the, 497; case of disease of the, occasioned by external violence, 506; case of compression of the, xxxii. 8; its connexion with animal life, 56; different injuries in it, upon sensation, 162; case of recovery from wounded, 184; case of protrusion of the, 525; observations on torpor of the, xxxiii. 51; on the morbid anatomy of the, 54, 143; case of abscess of the, 60; on the temperature of the, 119; child born without, 152; on its state in epileptics, 182; on Dr. Gall and Spurzheim's doctrine of the, 228, 485; effects of cold in the, 246; not sensative, 452; pressure retards its functions, 453; on the functions of separate parts of the, 492; how those properties are to be distinguished, 495; on the functions of the, xxxiv. 43; child born without, 104; communication between the two lateral ventricles of the, 309; case of diseased, 519.

BRAMINS, on their supposed knowledge of the cow-pox, xiii. 344; superstition of the, 570; not susceptible to the guinea-worm, and why, xvii. 172; their encouragement of vaccination, xxx. 316.

BRANCA, father and son, in the 15th century practised the art of renovating noses, ii. 156.

BRANDE, Everard, on a poisonous species of agaric, iii. 41.

———, William Thomas, his experiments on guaiacum, xv. 294; on detecting the colouring particles of madder in the urine, xx. 215; his experiments on calculi, xxii. 49; on the effects of magnesia in preventing the formation of uric acid, xxiv. 203; xxviii. 37; on the constituent parts of the blood, xxvii. 73, 168; account of a vegetable wax from Brazil, ibid.; his chemical researches on the blood, xxviii. 386; remarks on alcohol, xxix. 173; additional facts on magnesia in urinary calculus, xxx. 249, 324; syllabus of his lectures, 348; on the state of alcohol in fermented liquors, 392; on a change produced in oil by passing through the body, xxxi. 46, 47.

BRANDIS, Dr. his theory of dentition, iv. 77; on the puerperal fever, viii. 514.

BRANDRETH, Dr. on vaccine inoculation, viii. 546.

BRANDY, how obtained from beetroot, iv. 284; beneficial after violent exercise, xvi. 383; its power in counteracting the effects of alcohol, xix. 499.

BRAUNSCHWEIG, an old surgeon of the 16th century, his practice, ii. 55; account of, 278.

BRANSON, J. on the vaccine inoculation, v. 160.

BRATHWAITE, J. A. on oxygenated muriatic acid in scarlet fever, xi. 289.

BRAUN, M. his account of an alchemical manuscript, xiv. 95.

BRAZIL, on the constitution of the females of, iv. 321; vaccination introduced in, xvi. 173; account of a vegetable wax from, xxvii. 73; on the salt of, 76.

BREAD, substitute for it in poultices, iii. 164; on the art of making, xii. 479; substitutes for wheaten, xvi. 74; xvii. 73; xix. 77; xx. 269; on the composition and nutritive qualities of, xxii. 403; improvement in the art of making, 404; unknown in China, xxxiii. 248

BREAST, case of an inflamed, v. 239; case of schirrhous tumour in the, vii. 49; on the cancerous, 82, 163; abscess of the, occasioned by a fractured rib, x. 414; case of cancer of the, 448; on the treatment of schirrous tumours and cancer in the, xii. 75; night-shade good in indurations of the, 136; case of cancer in the, xiii. 364; history of a tumour in the, xiv. 82; observations on the cancerous, xv. 407; benefit of carbonate of iron in tumours of the, xvii. 201, 325; a Chinese remedy for complaints in the, xviii. 176; virtue of elecampane in disorders of the, xix. 455; effects of water on the schirrhous, xxii. 79; case of cancerous hydatids in the, 355; on removing schirrhous tumours of the, xxxi. 450; on cancer in the, xxxiv. 150.

BREASTS, singularity in a woman's, iii. 402; case of cancer in both, iv. 296; Dr. Nisbett's explanatory account of the case, 476; v. 76; reply to, 188; in puerperal fever, how to relieve, viii. 512.

BREATH, causes of bad, xiv. 477.

BREATHING, case of periodical difficulty of, viii. 401; observations on the organs of, xiii. 371; of gaseous oxide of azote, on the effects of, xvii. 52.

BREE, Dr. on the use of digitalis in consumption, ii. 314, 430; observations in reply to, iii. 121, 133; on disordered respiration, iv. 260; on the influenza, x. 125; on asthma and disordered respiration, xviii. 476, xx. 6; on the cause of purpura and its distinction from scurvy, xxi. 321; his animadversions on Dr. Rees, xxiv. 357; on smoaking tobacco, xxv. 231; on the effects of smoaking stramonium, xxvi. 51; on painful affections of the side, xxviii. 63; on a case of splenitis, xxx. 169.

BREFELD, Mr. on the use of oils in arthritic complaints, v. 490; use of cold water in dysentery, ibid.

BRENAN, James, on pulmonary polypi, viii. 360.

———, Dr. on the cure of puerperal fever by spirits of turpentine, xxxii. 403; observations on his remedy, xxxiii. 179; xxxiv. 183.

BRENTFORD, fossils found in the neighbourhood of, xxix. 507.

BRERA, Professor, on the use of medicated frictions, i. 71; on the Brunonian system, 200; resigns the chair at Pavia, 402; history of a case of hemiplegia, 412; on an epidemic among cats, ibid.; on the internal use of phosphorus, v. 396.

BRESLAU, success of vaccination at, ix. 130; xiv. 234; regulations for inoculation at, xvii. 58.

BREVEN, Mount, in Savoy, electrical phenomena on, xxxiii. 248.

BREWER, Mr. his account of the influenza in Monmouthshire, x. 522.

BREWERIES, their effects on the health, xvi. 101.

BREWSTER, Dr. his method of finding the rising and setting of the stars, xvii. 582; on the properties of the lever, xxv. 181; on new properties of light, xxix. 426; on the action of agate on light, xxx. 427.

BRICKELL, Dr. his theory of puerperal fever, i. 381; on alkalis in cases of poison, xiv. 478.

BRIDGE-END, Glamorganshire, influenza at, x. 230.

BRIDGEWATER, influenza at, x. 228.

BRIDGNORTH, influenza at, x. 220.

BRIDLE of the tongue, fatal effects of cutting the, xiv. 97; observations on the, 256.

BRIEUDE, M. on odorous exhalations, i. 243.

BRIGGS, Dr. Henry, on a case of tetanus cured by purgatives, xxi. 418.

———, James, on diseases of the eyes, xvi. 562; xvii. 79.

BRIGHT, Edward, of Maldon, his enormous size, xxv. 351.

BRINE PITS, description of the, xxviii. 74.

BRINGHURST, Joseph, on the efficacy of alkalis in bilious colic, viii. 293.

BRISTOL, account of the pneumatic institution at, ii. 480, 488; cases in the dispensary at, v. 317, 411, 509; vi. 4, 112, 196, 363, 389, 491; vii. 14, 304; on the atmosphere of, 12; plan of trying certain medicines at the pneumatic institution of, 165; diseases in the dispensary at the Hot-wells, vii. 301; on the influenza at, x. 80, 97, 102; vaccination at, xvii. 336; account of the pneumatic institution at, xxi. 188; meteorological observatory at, 435; xxiii. 255; xxv. 142; mortality at, xxii. 239; on the population of, xxviii. 308; account of the Madeira house at, xxxii. 61.

BRITAIN, etymology of the name, vii. 240; state of the practice of physic in, xvi. 279; rise and progress of the medical art in, xvii. 394; progress of medical knowledge in, xviii. 1; on the climate of, xx. 6; on the medical topography of, xxi. 3, 91; xxvii. 17; xxviii. 92; population of, xxviii. 307; annual mortality in the different counties of, xxx. 140.

BRITISH CRITIC, vindication of Dr. Bourne's book on uva ursi, against the, xvii. 288.

——— OIL, composition of, xiii. 502.

BRITONS, how they coloured their bodies, xviii. 173.

BROADBELT, Dr. on bronchocele, xxvi. 207.

BRODIE, Mr. on the spleen, xx. 213; his lecture on muscular motion, xxv. 180; on the effects of vegetable poisons, 361; his experiments on the influence of the brain, xxvi. 58; his experiments on vegetable poisons, 129; observations on his experiments, 278; review of his experiments, xxvii. 3; on the cruelty of them, 7; farther experiments on poisons, 427; his anatomical observations on the bladder, xxviii. 6, 14; further experiments on the action of poisons, 115; observations on the same, 179; influence of the brain on animal heat, xxix. 141; on the experiments of, 441; case of dissection of a consumptive person, xxxi. 156; influence of the nervous system on muscular motion, 168; influence of the nerves on the stomach, 338, 486; his refutation of Dr. Hale on animal heat, xxxii. 295; observations on his experiments, xxxiii. 241; on diseases affecting the joints, 508; on the experiments of, xxxiv. 56.

BRODUM, William, brings an action against the publisher for a libel, xiii. 192; on which the article respecting him was cancelled, and a new one inserted, 258; elegant letter written by, xix. 428.

BRODY, P. W. his cases in surgery, xii. 514.

BROERLAND, M. on the virtues of the bark of bird-cherry, xvi. 76.

BROMFIELD, Mr. his death occasioned by bronchocele, v. 38; his case of a stone in a woman's bladder, xi. 18; anecdote of, xxxi. 453.

BROMPTON, meteorological observations at, xii. 381, 480, 571; xiii. 185, 385, 480.

BRONCHIAL GLANDS, on the black colouring matter of the, xxxi. 128.

——— POLYPI, cases of, with remarks, xx. 471.

BRONCHITIS, symptoms of, xxiv. 21.

BRONCHOCELE, on the use of burnt sponge in, iv. 412; v. 38, 355; xii. 9; xiii. 13; on the causes of, v. 38, 175, 355; setons recommended in, viii. 495; on its connexion with scrofula, xii. 9; women more liable to it than men, xvi. 207; its effects on the brain, 208; on an external remedy for, xxvii. 114.

BRONCHOTOMY, on a case of, iii. 228; observations on the practice of, x. 6.

BRONGNIART, M. his dissection of apes, ii. 480; on rendering odorous effluvia visible, iii. 89; affinities of oxygen and carbon, 374; on describing mineral substances, 375; on the inhabitants of the Alps, ibid.

BROOKES, Joshua, his models of the organs of sense, xx. 3; on white serum of the blood, 151; his remarks on fossil remains, xxv. 97; statutes of his medical society, xxvi. 504; his account of seven new species of shells, xxix. 429; account of his museum, xxxi. 259; his discovery of an antiseptic process, 521.

BROOKLIME, botanical description of, xi. 371; antiscorbutic qualities of, 372.

BROOM, the green, efficacious in consumption, i. 382; and in dropsy, 383; botanical description of the, xix. 73; beneficial in dropsy, 74.

BROTH, method of preparing it from bones, xii. 350.

BROUGHTON, S. D. cases of hydrophobia in the East Indies, xix. 492.

BROUSSAIS, Dr. his case of tapeworms, xxxiii. 7.

BROUSSONNET, Dr. process for colouring morocco skins, i. 201; on the use of scarifying and cupping, x. 425.

BROWN, roots of the water-lily used in dying a dark, xvii. 69.

———, B. on the success of bleeding in difficult parturition, xxi. 179.

———, Charles, on suspended animation, i. 59; his case of apoplexy, 331; case of obstruction in the ureters, 448; on the importance of anatomical knowledge, ii. 142; on hydrocephalus internus, 258, 327; on a fatal concussion of the brain, 407; his observations on the Zoonomia, 276; on an hospital for ulcerated legs, iii. 135; reply to him on Zoonomia, 139; on the use of yeast in typhus, 533; his idea of a sedative unfounded, xi. 145.

———, Dr. David, on the yellow fever, xiv. 389; answer to, xv. 40; his reply, 560.

———, Dr. John, character of, by Dr. Rush, iii. 186; remarks on the practice of, iv. 269; parallel between Asclepiades and, v. 373; on fever and rheumatism, 388, 577; controversy between him and Cullen, vii. 368; his theory of fever, 388; melancholy fate of, viii. 353; treatise on his system, by Dr. Pfaff, 563; ridiculed the idea of sedatives, xi. 145; confession of, xiii. 568; on his philosophy, xv. 91; did not understand his own system, xvi. 182; his strange assertion on consumptive persons, xix. 265; vindication of, 304; errors of his theory, xxvi. 36.

———, Dr. Joseph, on the toothache, iii. 403; on the use of yeast in typhus, 533; on lime-water as a remedy for ascarides, vii. 256; on his cure for fistula in ano, xxvii. 75; on opium in ophthalmia, xxxiv. 10.

———, Dr. Samuel, his case of tetanus from a wound in the heel, xix. 423.

———, Dr. W. C. his case of constipation, xxi. 480; on the condition of navy surgeons, xxxii. 416.

———, Thomas, on vaccination, xxiv. 163.

———, Mr. of Sunderland, on the influenza at that place, x. 118.

———, Mr. his extensive knowledge of the proteaceæ, xxiv. 440; on the

Asclepiadeæ, xxvi. 257; on peculiarities in the structure of seeds, xxix. 509; his researches in New Holland, xxx. 524.

————, Mr. his instrument for compressing the subclavian artery, xxvii. 430; his cases of small-pox after vaccination, xxix. 231.

BROWNE, John, surgeon to Charles II. his curious account of the royal touch for the evil, xvii. 477.

————, (Plymouth,) his case of wounded artery, xxi. 317.

————, Mr. account of his travels in Africa, i. 402; on an endemial disease in Egypt, v. 4; anecdotes of, xxxiv. 214.

BRUANT, M. on the ophthalmia of Egypt, vii. 212.

BRUCE, James, his description of the balm of Gilead, i. 33, 35; his account of the leprosy, ix. 565.

BRUFE, Mr. description of his instrument for drawing teeth, i. 87.

BRUGERIUS, M. on venereal complaints in Italy, xxxi. 411.

BRUGGS, Professor, gives offence to the National Institute, and why, i. 195.

BRUGMANS, Dr. on the causes of hospital gangrene, xxxiv. 394, 467.

BRUGNATELLI, M. on the combustion of phosphorus, i. 85; his discourse on light, 195; on the detonating power of phosphorus, ibid.; on calculous affections, ii. 75; discovers a new resinous substance, 77; freezes mercury, ibid.; on the inflammation of ether, 78; on limewater, 79; on the property of azote, 185; method of preparing solid gold, ibid.; on the preparation of concentrated lemon juice, iii. 171; his new arrangement of mineral waters, iv. 377; discovers the conversion of tartarous acid into the saccharic acid, vi. 475; his experiments on animal fluids, xi. 287; on treating paper with nitric acid, 288; on galvanism, xiii. 373.

BRUISES, on the general treatment of, xiii. 385; effects of sal ammoniac and vinegar in, 508.

BRUNINGHAUSEN, Professor, his case of convulsion cured by alkalis, v. 472.

BRUNONIAN SYSTEM, its influence on the practice of medicine, i. 41; leading peculiarities of the, 124; vindication of the, 130; overrated by its admirers, 192; splendid work on, 200; not altogether new, 487; on the principles of, 489; founded on observation, ii. 449; remarks on the, iii. 47; on the practice of the, iv. 269; apology for the, 273; criticism on the, 367; deception of the, v. 131; observations on, 255; imposition to recommend it, vii. 93; curious instance of the practice of, 407; account of Pfaff's treatise on, viii. 563; its increase deprecated, ix. 344; embraced in Italy, and why, 434; on its introduction at Edinburgh, x. 181; justification of it, xiii. 569; characteristics of the, xvi. 182; objections to, xviii. 464; on the decline of the, xxvi. 36; disguised by Darwin, xxviii. 324.

BRUSH, description of a metallic, vi. 91.

BRUSSELS, prize questions of the Medical Society of, xiv. 382; xxvi. 345; mortality at, xxxiv. 349.

BRUTES, the small-pox common to men and, viii. 271.

BRUTUS, on the spectre said to have appeared to, xxiv. 161.

BRUYN, on the medical secret of de, xxvii. 118.

BRYANT, Thomas, his fatal case of hooping-cough, xxxiii. 358.

BRYCE, James, on the inoculation for the cow-pox, viii. 373; his cases of variolous inoculation after vaccination, xxvii. 164; efficacy of the dry crust in vaccine inoculation, xiii. 49; xxx. 508; observations on the fœtal liver, xxxiii. 224.

BRYDONE, Patrick, his account of the sirocco, xxxiv. 352, 356.

BRYER, S. T. on a case of bronchotomy, iii. 228.

BRYONY, botanical description of, xi. 447; xiv. 69.

BUBOES, living snails applied beneficially to exulcerated, vi. 93; on pestilential, 260; on the stench of, 443; on the nature and management of venereal, xii. 279; efficacy of sulphat of soda in, xiii. 38; on the treatment of, with mercury, 396.

BUBONARY FORM, why the plague assumes a, x. 465.

BUBONOCELE, probability of curing the, ix. 161; Mr. Carlisle first suggested its cure, 292; Mr. Cooper on the radical cure of the, 367; observations of the editors on the question, 392; Mr. Carlisle in explanation on, 396; case of incarcerated, xi. 263; case of one that required a second operation, xxxiii. 411.

BUCHAN, Dr. on the danger of drinking cold liquors, xvi. 383; his aphorisms of health and life, xxviii. 13.

BUCHANAN, Dr. on an Indian recipe for the tape-worm, xvii. 189; his case of encysted tumour of the brain, xxviii. 502.

BUCHHOLZ, Dr. his method of purifying acid of tartar, iv. 181; easy mode of obtaining emetic tartar, viii. 92 ; on the preparation of the mild sublimate of mercury, ix. 81 ; on the chemical analysis of opium, 398 ; x. 42 ; on the metal of cobalt, 94 ; method of preparing arsenic acid, xi. 382 ; on the distillation of opium, xii. 42 ; his new method of preparing emetic tartar, 189 ; experiments on mercurius dulcis, 574 ; on the preparation of naphtha aceti, xiv. 383 ; his experiments on camphoric acid, xxvii. 127 ; analysis of a new bitumen, xxxii. 81.

BUCHNER, Mr. on vaccination in Norway, xvi. 478.

BUCK, Dr. his case of a foreign substance in the lungs, xxxii. 526.

BUCKBEAN, its juice beneficial in gout and rheumatism, xii. 127.

BUCKNELL, S. his case of periodical catalepsy, xxviii. 171 ; observations on that case, 378.

BUCKNILL, William, his case of poison by arsenic, xxix. 44.

BUCKTHORN, its cathartic powers, xii. 221.

BUCKWHEAT, botanical description of, xv. 67.

BUDDING, new and expeditious mode of, xxv. 253.

BUFFON, M. his observations on squinting, xix. 312 ; on the resemblance between man and the ape, xxi. 511 ; character of, xxvii. 25.

BUGS, the bites of them mistaken for an eruptive disease, xv. 520.

BULBS OF PLANTS, observations on, viii. 475.

BULLET-FORCEPS, description of an improved, vii. 554.

BULLOCK'S MUSEUM, rare articles of natural history in, xxv. 100 ; account of curiosities in, xxvi. 16 ; particular description of, xxvii. 341 ; necessity of a catalogue of the, 380 ; companion to, 516.

BULLOCK, Mr. his account of a fossil turtle, xxix. 511.

BULLUS, Dr. on bleeding in laborious parturition, xx. 77.

BUNIVA, Dr. on animal life, iii. 272 ; his experiments on blood vessels, 587 ; on the epizootic disease of cats, iv. 373.

BUNOSUS, case of, apprehended spurious, xiii. 399.

BURDACH, Dr. his parallel of Brown and Asclepiades, v. 373.

BURDER, Dr. on syphilis, xxxiv. 348.

BURDOCK, botanical description of the, xii. 231 ; diuretic qualities of the, xix. 259.

BURKE, Dr. on the yellow fever, ix. 37.

———, William, on amphibious animals, xiii. 370 ; on the respiration of fishes, 371.

BURNESTER, Mr. his case of a man who died under mild punishment, xviii. 176.

BURNE, John, his case of injury of the head, xxvii. 521 ; further particulars of that case, xxviii. 86.

BURNET GRASS, useful in dysenteries and hæmorrhages, xxvii. 52.

BURNETT, Dr. on the bilious remittents in the Mediterranean fleet, xxxi. 489 ; on consumption, xxxiv. 91.

———, Gilbert, tried for exposing children in the small-pox, xxxiv. 83.

BURNS, cold water recommended in, iii. 191 ; benefit of oil of turpentine in, 206, 296 ; E. Kentish on the use of the same, 282 ; iv. 454 ; produced by lightning, healed with alkalies, iv. 371 ; success of stimulant applications in, v. 55 ; not a new practice, 236 ; best method of applying turpentine in, 237 ; on cold applications in, 238 ; benefits of cold water in, viii. 443 ; ivy a remedy for, xii. 226 ; successful application of turpentine in, xiii. 14 ; case of the benefit of the same, xiv. 34 ; Dr. Kentish on the superiority of his practice in, 396 ; his method recommended, xv. 146 ; objections to it, 342 ; defended, 458 ; explanation in justification of it, xvi. 8 ; Dr. Kinglake on the cool treatment of, 17 ; virtue of houseleek in, 76 ; reply to Dr. Kinglake on the cold regimen in, 115 ; answer of Dr. Kinglake, 210, 460 ; successful cases of the stimulant treatment of, 313 ; Dr. Evans on the cooling treatment of, 461 ; cases of the benefit of that mode, 462, 465, 526 ; attempt to reconcile the two systems, xvii. 1 ; benefit of cerussa acetata in, 21 ; the terebinthinate practice not new, 131 ; benefits of the cooling plan in, 132, 320 ; observations on the variety of the treatment of, 321 ; on the virtue of cold in, 440 ; curious case, in which both practices failed, xviii. 67 ; observations on that case, 159, 160 ; additional remarks and cases in favour of the cooling treatment, 207 ; union of the hot and cold plans recommended, 238 ; history of a case treated on Dr. Kentish's plan, 304 ; on the relative merits of both plans, 361 ; no reliance upon any particular method of treatment, 405 ; case in which turpentine was successful, 409 ; remarks on the case, 413 ; on the aqueous and terebinthinate treatment of, xix. 17 ; Dr. Wood's claim to priority of discovery of the stimulant plan, 229 ; Dr. Kentish in reply to Dr. Wood, 306, 417 ; Dr. Wood in explanation and defence of his claim, 518; observations on that dispute, xx. 53; neither of the practices new, 54; Dr. Kentish in reply, 56; superiority of the terebinthinate practice in, 491; on the use of stimulants in, xxi. 387; efficacy of alcohol in, xxiv. 99; case of deformity from, xxvi. 158; benefit of the stimulant plan in, 499; oil of turpentine found to be unsuccessful in America, xxviii. 424; on the use of cotton in, xxix. 137; case of extensive, 196; on contraction after, xxxiii. 65.

BURNS, Allan, observations on his work on crural hernia, xvi. 119; on the structure of the parts in crural hernia, 172; vindication of his practice, xvii. 23; on diseases of the heart, xxii. 505; xxiv. 27; on the digestion of the stomach after death, xxiii. 429; Dr. Adams in answer to, 399; on the fungus hœmatodes, xxiv. 11; his principles of midwifery, 33; case of suppuration of the liver, xxv. 164; on the anatomy of the head and neck, xxviii. 11.

———, John, on the anatomy of the gravid uterus, iii. 478; on the formation of the human ovum, xv. 271; reply to, on cancer, xvi. 99; observations on cow-pox, xvii. 471; on inflammation, xviii. 22; on the uterine hæmorrhage, 281; on the forceps, xxvi. 112; on the efficacy of spirits of turpentine in puerperal fever, xxxii. 409.

———, Robert, on the talents and fate of, v. 98.

———, Margaret, remarkable case of, xxi. 336; observations on the case and trial, 399; controversy concerning, 426; xxii. 118; farther observations on that case, xxxii. 327.

BURRELL, Dr. his case of scirrhus in the intestines, xxx. 515.

BURROUGHS, G. F. on an inflammatory fever in Portugal, xxxii. 267.

———, G. on the Eau Medicinale in gout, xxvi. 499.

———, B. T. on curved spine, xxix. 415.

———, Mr. his account of the influenza, x. 224.

BURROW, Mr. his case of hernia cerebri, xxviii. 59.

BURROWS, G. M. his correspondence as chairman of the committee of Apothecaries, xxx. 387, 392, 472, 477; letter to, 479.

BURSÆ MUCOSÆ, description of the, iii. 386; account of a disease of one of the, x. 69.

BURSERIUS, John Baptist, memoirs of him, and his writings, ii. 334.

BURT, Dr. on a new blistering fly, xxiv. 430.

BURTON, Dr. on bleeding in hydrophobia, xiv. 122; observations on his case, xv. 413.

BUSH, Mr. his case of dropsy, x. 498; on the treatment of hernia umbilicalis, xx. 312; remarks on it, 405; his case of a knife lodged in the back, xxviii. 63; his description of a cradle for fractured legs, xxxiii. 169.

BUSSORA, success of vaccination at, ix. 450.

BUST, mechanism of the speaking, xvii. 199.

BUTLER, Mr. his case of inoculation at Woolwich, xii. 531.

BUTTER, Dr. his cases of chin-cough, xxxi. 59.

BUTTER-CUPS, botanical description of, xvii. 373; blistering properties of, 374.

BUTTERWORT, its juice a remedy against lice, xi. 439; the cause of the rot in sheep, 440.

BUXTON, Dr. on a regulated temperature in consumption, xxiii. 150; his cases of pulmonary consumption, xxvii. 159; xxix. 376, 391; remarks on his practice, xxx. 49; on his method of treating consumption, xxxiii. 202; on the effects of temperature, in reply to Dr. Sutton, 365; answer to, xxxiv. 89; his rejoinder, 368.

BYSSUS, botanical description of the white cobweb, xx. 351; used in Ireland as a vulnerary, 352.

BYTHELL, Mr. on the use of the elastic gum catheter, xxxi. 170.

